# Photomodulation of bacterial growth and biofilm formation using carbohydrate-based surfactants†
†Electronic supplementary information (ESI) available: Experimental procedures, supplementary tables, figures and spectra. See DOI: 10.1039/c6sc03020c



**DOI:** 10.1039/c6sc03020c

**Published:** 2016-08-17

**Authors:** Yingxue Hu, Wenyue Zou, Villy Julita, Rajesh Ramanathan, Rico F. Tabor, Reece Nixon-Luke, Gary Bryant, Vipul Bansal, Brendan L. Wilkinson

**Affiliations:** a School of Chemistry , Monash University , Victoria 3800 , Australia; b Ian Potter NanoBioSensing Facility , NanoBiotechnology Research Laboratory , School of Science , RMIT University , Victoria 3000 , Australia . Email: vipul.bansal@rmit.edu.au; c Centre for Molecular and Nanoscale Physics , School of Science , RMIT University , Victoria 3000 , Australia; d School of Science and Technology , The University of New England , New South Wales 2351 , Australia . Email: Brendan.wilkinson@une.edu.au

## Abstract

Naturally occurring and synthetic carbohydrate amphiphiles have emerged as a promising class of antimicrobial and antiadhesive agents that act through a number of dynamic and often poorly understood mechanisms. In this paper, we provide the first report on the application of azobenzene *trans*–*cis* photoisomerization for effecting spatial and temporal control over bacterial growth and biofilm formation using carbohydrate-based surfactants. Photocontrollable surface tension studies and small angle neutron scattering (SANS) revealed the diverse geometries and dimensions of self-assemblies (micelles) made possible through variation of the head group and UV-visible light irradiation. Using these light-addressable amphiphiles, we demonstrate optical control over the antibacterial activity and formation of biofilms against multi-drug resistant (MDR) *Pseudomonas aeruginosa*, methicillin-resistant *Staphylococcus aureus* (MRSA) and Gram-negative *Escherichia coli*. To probe the mechanism of bioactivity further, we evaluated the impact of *trans*–*cis* photoisomerization in these surfactants on bacterial motility and revealed photomodulated enhancement in swarming motility in *P. aeruginosa*. These light-responsive amphiphiles should attract significant interest as a new class of antibacterial agents and as investigational tools for probing the complex mechanisms underpinning bacterial adhesion and biofilm formation.

## Abstract 2

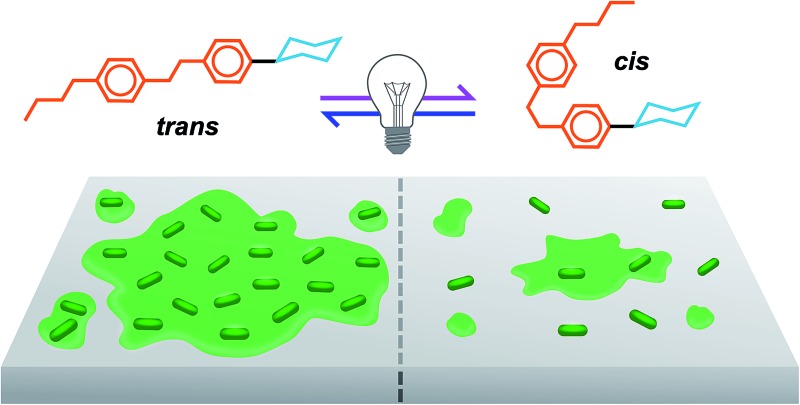
The photocontrollable antibacterial and biofilm modulatory activity of a panel of light responsive carbohydrate-based surfactants is reported.

## Introduction

Antimicrobial drug resistance represents a global health emergency and there is now an urgent and unmet need for new clinical agents and preventative strategies that possess unconventional modes of action.^1^ This problem is exacerbated by the ability of many pathogenic bacteria to form matrix-enclosed communities that are immobilized on biotic and abiotic surfaces, or biofilms, which confer enhanced resistance toward host immune responses and antibiotic treatments.^2^ Naturally occurring biosurfactants are a structurally diverse class of amphiphilic compound with promising anti-microbial and anti-biofilm activity. In particular, microbial glycolipids and their synthetic analogues have attracted considerable interest as selective antibacterial agents and as mechanistic tools for expanding our understanding of bacterial physiology and biofilm formation.^3,4^ In many cases, this bioactivity can be attributed to the ability of these molecules to lower interfacial tension, thereby mediating bacterial motility, cellular and protein adhesion, signalling and communication, pH regulation, nutrient uptake, and degradation of harmful metabolites.^3–6^ These multifaceted responses are dependent on the bacterial strain and surfactant used, and our current understanding is limited to a handful of well-characterized systems. Access to chemical tools with tuneable properties that could selectively influence bacterial growth and biofilm formation offers new opportunities to develop novel therapies for biofilm-associated diseases, and to study the mechanisms governing bacterial adhesion and biofilm formation.

In recent years there has been considerable interest in light as an external stimulus for effecting spatial and temporal control over the conformational dynamics of biomolecules^7^ and the biological activity of small molecules.^8^ Toward this goal, the well-studied azobenzene chromophore has been actively pursued, owing to the facile and reproducible *trans*–*cis* photoisomerization, and the ease of molecular synthesis for enabling new light-addressable materials with tailored photoswitching wavelengths and potential *in vivo* applications.^9,10^ In parallel with these developments, azobenzene photochromism has been widely reported as a means for modulating carbohydrate multivalency and lectin binding,^11^ and for controlling supramolecular self-assembly^12^ and host–guest interactions.^13^ We have previously demonstrated the photocontrollable self-assembly and interfacial activity of carbohydrate-based surfactants and fluorosurfactants.^14^ Throughout the course of these studies, we demonstrated the ability to modulate the interfacial activity and aggregation properties of these amphiphiles through changes in head group geometry, size and polarity, as well as the isomeric state of the tail group.^15^ However, the impact of azobenzene *trans*–*cis* photoisomerization within carbohydrate-based surfactants on the ability of bacteria to survive and to form active biofilms remains to be verified.

**Fig. 1:**
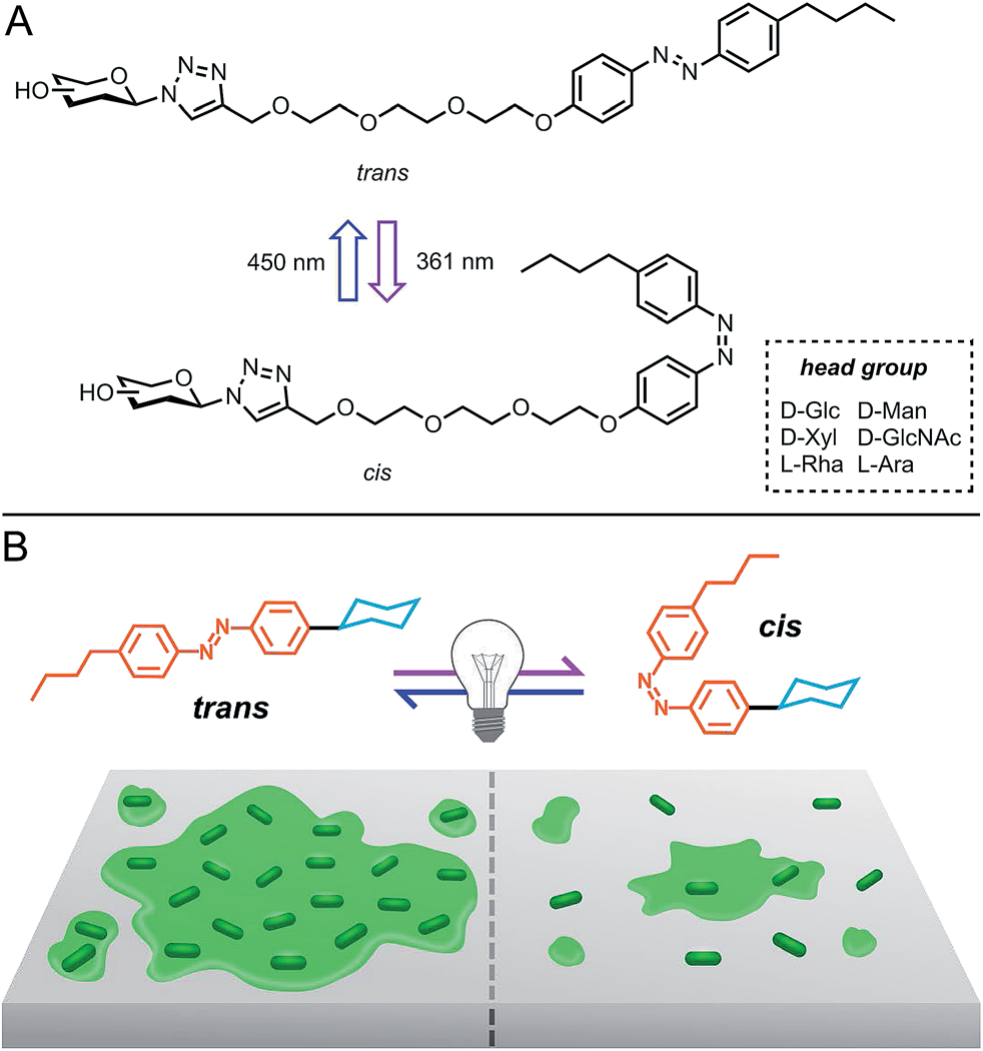
(A) Photoswitchable carbohydrate-based surfactants and (B) cartoon representation of the photomodulation of biofilm growth.

**Fig. 2:**
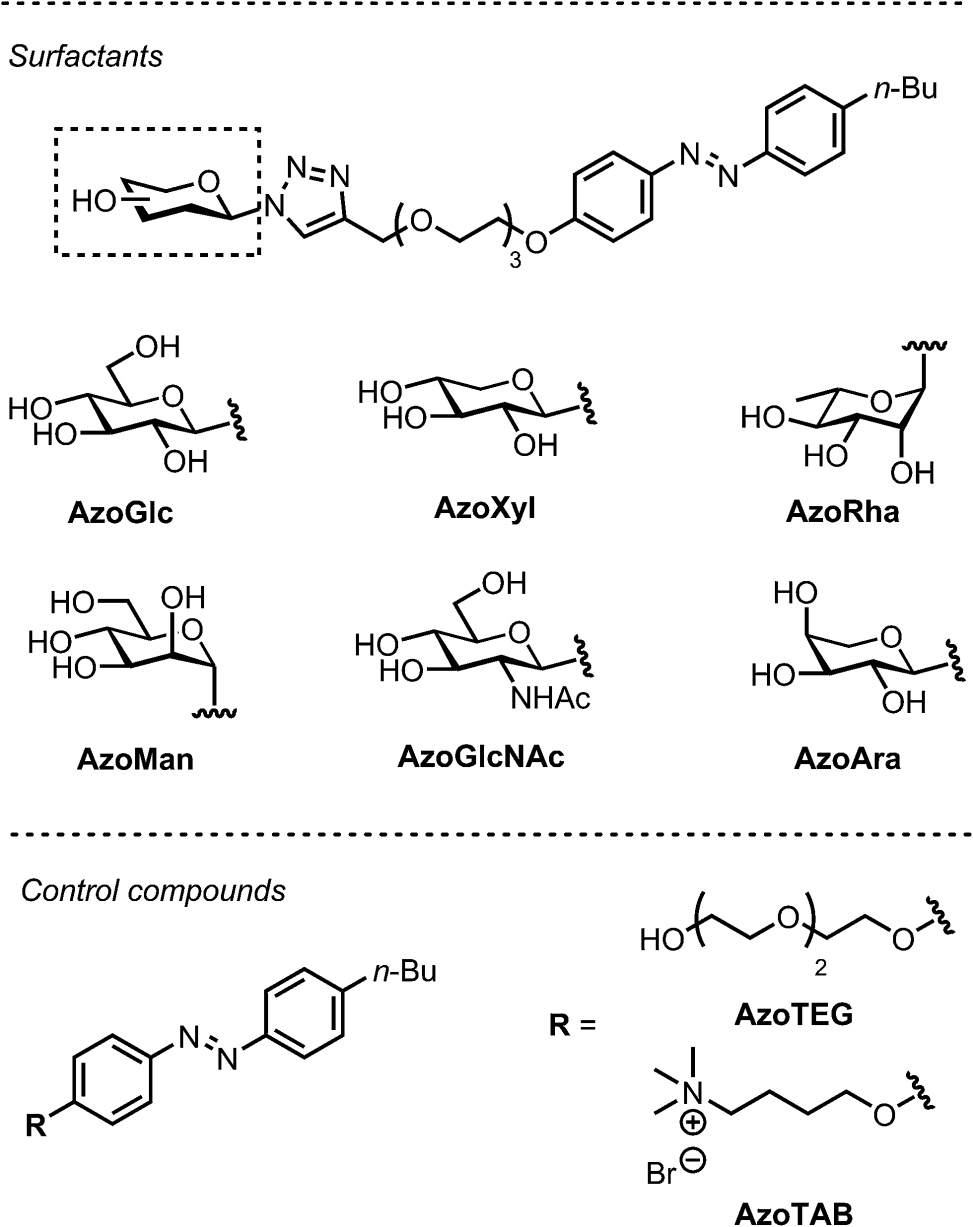
Photoswitchable carbohydrate-based surfactants and control compounds used in this study.

Herein, we report the photocontrollable antibacterial and biofilm modulatory activity of a panel of carbohydrate-based surfactants (Fig. 1). Surfactants incorporated variable monosaccharide head groups, including d-glucose (**AzoGlc**),^14^ d-xylose (**AzoXyl**), l-rhamnose (**AzoRha**), d-mannose (**AzoMan**), *N*-acetyl glucosamine (**AzoGlcNAc**), and l-arabinopyranose (**AzoAra**), which were tethered to a hydrophobic *n*-butylazobenzene tail group (Fig. 2). Monosaccharide head groups were selected based the natural occurrence and intriguing biological activity of glycolipids expressing these structures, and the important roles carbohydrates play in bacterial adhesion processes. Through reversible photocontrol over self-assembly and interfacial activity, these surfactants were assessed as modulators of bacterial growth and biofilm formation against methicillin resistant *Staphylococcus aureus* ATCC1698 (MRSA), *Escherichia coli* DH5α and a multi-drug resistant strain of *Pseudomonas aeruginosa* MDR283/1-6.

## Results and discussion

### Surfactant synthesis and characterisation

Our investigation commenced with the modular synthesis and physical characterisation of a panel of photoswitchable carbohydrate-based surfactants (see ESI for details†). To this end, known glycosyl azides were united with a *n*-butylazobenzene tail fragment using the well-established Cu(i)-catalysed azide-alkyne cycloaddition reaction (CuAAc).^14,16^ Surfactants were isolated in acceptable yields over two steps following purification by reversed phase, preparative HPLC. In addition to non-ionic carbohydrate photosurfactants, we also targeted a non-surface active fragment lacking the sugar head group (**AzoTEG**) and a known cationic photosurfactant (**AzoTAB**)^17^ as control compounds.

Having acquired surfactants, we assessed the photocontrollable surface activity and self-assembly properties using pendant drop tensiometry and small angle neutron scattering (SANS), respectively. To affect *trans*–*cis* photoisomerization, compounds were irradiated at 361 nm for 15 min and the critical micellar concentration (CMC) measured using pendant drop tensiometry, although one minute was found to be sufficient to achieve near complete *trans*–*cis* photoisomerization. Photoisomerization was also observed using UV-vis spectroscopy as determined by a diminished intensity of the peak at 350 nm corresponding to the π–π* transition and an increase in intensity of the peak at 440 nm corresponding to the n–π* transition. The ratio of *cis* and *trans* isomers in either photostationary state (PSS) was estimated by integrating selected signals in the ^1^H NMR spectrum, before and after UV photoirradiation (ESI, Fig. S1†). In the *trans* dominated PSS, <10% of molecules existed in the *cis* isomeric form. Following UV irradiation, approximately 75% of molecules existed in the *cis* isomeric form (*cis* dominated PSS).^18,19^ To investigate potential photodegradation of surfactants during photoswitching, the ^1^H NMR spectra of the *trans* isomer was recorded both prior to UV photoirradiation, and following visible light irradiation (430 nm) of the photo-excited *cis* isomer (ESI, Fig. S2†).

Since our antibacterial assays occurred in dark-adapted conditions at 37 °C, we were interested in monitoring the thermal relaxation of the *cis* isomer to the more stable *trans* isomer in bacterial growth media over a 24 h time period. The *cis* isomers showed a half-life of over 10 hours in water as well as in two bacterial growth media at 37 °C (ESI, Table S1 and Fig. S4–S7†). Considering the typical bacterial doubling time of 0.5–2 h, this would offer ample opportunity for *cis* isomers to exert a biological response. An increase in the CMC was accompanied by *trans*–*cis* photoisomerization, which was attributed to the increased polarity and altered molecular geometry of the *cis* isomer relative to the more hydrophobic and planar *trans* isomer (Table 1).^20^ However, in some cases the magnitude of this change was significant, in particular that of **AzoGlcNAc** incorporating an *N*-acetyl glucosamine head group. In contrast, virtually no difference in the CMC was observed between the *trans* (0.33 mM) and *cis* (0.34 mM) dominated PSS of **AzoXyl**, further highlighting the effect of subtle head group configuration and polarity on the interfacial activity of this class of photosurfactants.

**Table 1:** Photocontrollable surface and aggregation properties of carbohydrate-based photosurfactants^*a*^

Surfactant	E/C^*b*^	*N*_agg_	*A*_hg_	CMC (CMC_UV_)
**AzoGlc** ^17^	E	440	0.69	0.31 (0.59)
**AzoXyl**	C	NA	NA	0.33 (0.34)
**AzoRha**	C	622	0.79	0.11 (0.42)
**AzoMan**	E	141	0.84	0.13 (0.49)
**AzoGlcNAc**	E	178	1.02	0.11 (1.22)
**AzoAra**	E	89	1.12	0.33 (0.88)

^*a*^Aggregation number (*N*_agg_), area per head group (*A*_hg_) [nm^2^] and critical micelle concentration (CMC) in ambient and UV-irradiated conditions [mM].

^*b*^E = ellipsoid, C = cylindrical. For **AzoXyl**, long worm-like micelles were obtained with a length of >100 nm.

### Characterisation of self-assemblies

**Fig. 3:**
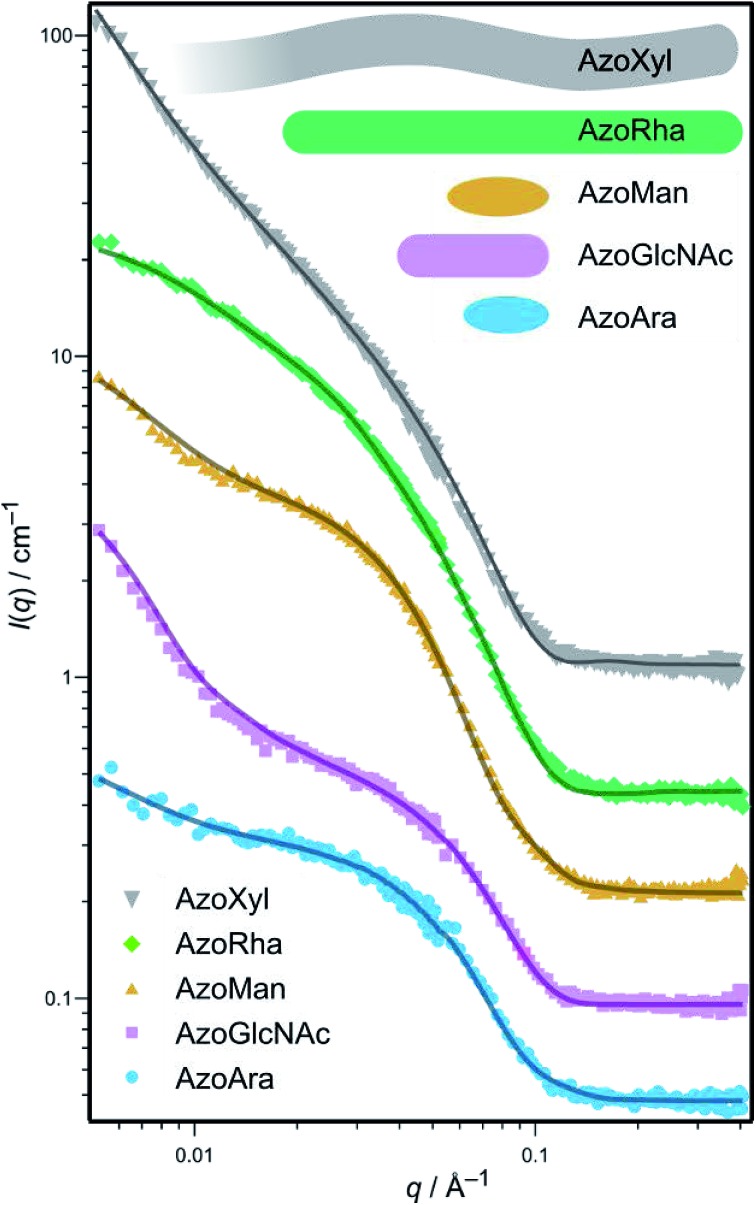
Small angle neutron scattering (SANS) spectrum of carbohydrate-based surfactants in D_2_O at 4 mM concentration (symbols) and theoretical fits (solid lines) for selected surfactant micelles. Inset top: meridional sections of the fitted geometries from SANS of the surfactants. The SANS spectra for **AzoGlc** has been reported previously.^17^

Small angle neutron scattering (SANS) is a powerful experimental technique that is frequently employed to deduce the morphology and size of nanostructures and self-assemblies in solution.^14,21^ SANS measurements were performed to determine the post-CMC aggregation structures (micelles) formed by the photoswitchable surfactants, which provided information on the number and topology of carbohydrates exposed on the micelle surface. We postulated that this information could be used to probe the potential relationship between the aggregation properties of these amphiphiles and the ability to modulate bacterial growth and biofilm formation. For each surfactant, spectra were acquired in D_2_O in the *trans*-dominated PSS at a concentration well above the CMC. As revealed from the SANS data (Table 1 and Fig. 3), a wide range of aggregate morphologies and sizes are made possible through variation of the carbohydrate head group, which is in agreement with our previous studies.^14,16^ Indeed, micelles ranged from small ellipsoids, as in **AzoMan** and **AzoAra**, to extremely long, flexible cylindrical (worm-like) micelles arising from **AzoXyl** (length > 100 nm, persistence length 36 nm). This extraordinary variety of self-assembled structures indicates that the aggregation properties of carbohydrate amphiphiles are extremely sensitive to the head-group employed, offering considerable geometric information on preferred packing and interfacial curvature.

### Photocontrollable antibacterial studies

We next evaluated the photocontrollable antibacterial activity of photoswitchable surfactants, non-surface active tail fragment **AzoTEG** and the cationic surfactant **AzoTAB** against Gram-negative *E. coli* DH5α, Gram-negative *P. aeruginosa*, and Gram-positive *S. aureus*. Compounds were screened for antibacterial activity using an OD_600_ bacterial growth assay (see ESI for experimental procedure and data†).^22^ To obtain *cis* photo-isomers for antibacterial studies, the native compounds were irradiated with UV light for 5 min prior to incubation with bacteria. Following a dark-adapted 24 h incubation period, the optical density of the wells was measured at 600 nm. **AzoTEG** displayed moderate inhibitory potency against *S. aureus* (MIC 125 μg mL^–1^), and limited-to-no inhibition of *P. aeruginosa* (MIC > 1000 μg mL^–1^) and *E. coli* (MIC > 2000 μg mL^–1^) in the *trans* PSS. Following photoisomerization to the *cis* isomer, a further increase in inhibitory potency against *S. aureus* was observed (31 μg mL^–1^), while no change in activity against *E. coli* and *P. aeruginosa* was noted. The enhanced antibacterial activity of the *cis* isomer of this compound corroborates well with a previous study on photoswitchable quinolone antibiotics.^8*c*^ In contrast to the non-surface active fragment, the cationic photosurfactant **AzoTAB** displayed potent antibacterial activity in the *trans* PSS against *E. coli* (MIC 12.5 μg mL^–1^), *S. aureus* (MIC 1.6 μg mL^–1^), and *P. aeruginosa* (MIC 31.2 μg mL^–1^). While no photomodulation of this activity was observed against *E. coli* and *S. aureus*, the *cis* PSS of **AzoTAB** became less toxic against *P. aeruginosa* (MIC 125 μg mL^–1^). The high toxicity of the cationic surfactant against *E. coli* and *S. aureus* indicates a non-selective mode of action through cell wall damage and lysis,^23^ whereas its lower toxicity against *P. aeruginosa* allows its activity to be photomodulated, possibly *via* reduction in the interfacial activity due to the enrichment of the *cis* isomer.^17^

**Fig. 4:**
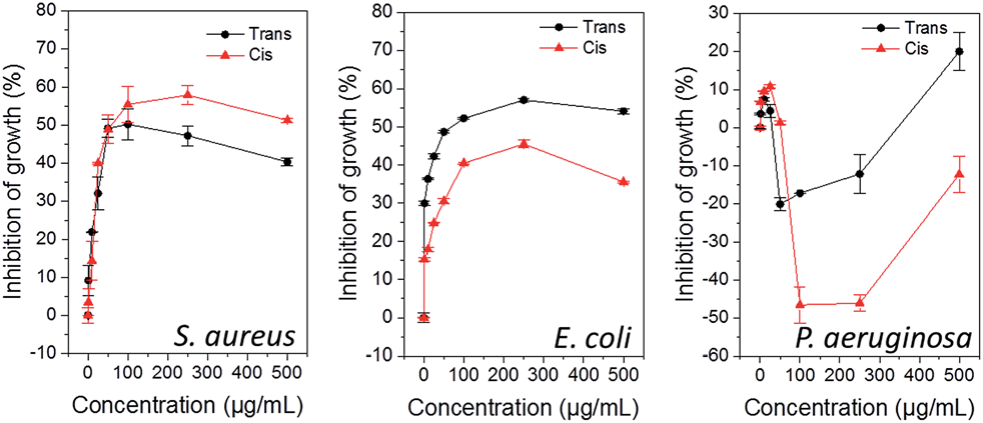
Photocontrollable influence of *cis*- and *trans*-dominated photostationary states of **AzoMan** on bacterial activity after 24 h.

The carbohydrate-based surfactants exhibited bacteria- and photoisomer-specific, dose-dependent inhibition of growth against both *S. aureus* (ESI, Fig. S7†) and *E. coli* (ESI, Fig. S8†), whereas they generally promoted growth of *P. aeruginosa* (ESI, Fig. S9†). Representative data for **AzoMan** is shown in Fig. 4. While at lower concentrations, surfactants showed nearly similar activities in the *trans*- and *cis*-dominated PSS against all three bacteria, the bactericidal activity could be modulated following photoisomerization at concentrations approaching and above the CMC. In the case of *S. aureus*, the photoexcited *cis* form was generally more toxic, with the exception of **AzoGlcNAc**, which was more toxic in its native *trans* form. Conversely, these compounds were typically more potent in their native *trans* state against *E. coli*, with the exception of **AzoRha** and **AzoGlcNAc** which showed higher antibacterial activity after photoisomerization. Surprisingly, all carbohydrate-based surfactants did not display significant toxicity against *P. aeruginosa* (with the exception of **AzoAra**) and generally promoted bacterial growth, especially in the *cis* photostate. Interestingly, the l-rhamnose based surfactant **AzoRha** was most influential in promoting growth of this bacterium. Whilst it is difficult to rationalize the heterogeneous responses observed with these amphiphiles against Gram-negative and Gram-positive bacteria, particularly those of **AzoRha** and **AzoGlcNAc**, it is interesting to note that these surfactants incorporate l-rhamnose and *N*-acetyl glucosamine as monosaccharide head groups, respectively. These carbohydrates have been known to play important roles in biofilm growth, adhesion and virulence in Gram-negative and Gram-positive species. For example, *N*-acetyl glucosamine is a major constituent of the cell wall and the extracellular matrix in both Gram-positive and Gram-negative bacteria and plays important roles in virulence and biofilm formation in both groups of bacteria.^24^ Gram-negative bacteria, in particular *Pseudomonas* spp., produce surface-active rhamnolipids as virulence factors that regulate swarming motility, intercellular signalling and biofilm formation.^4,25^ As such, the significance of these carbohydrate head groups in bacterial evolution might be responsible for their selective mode of interaction with these bacteria. Whilst the cationic surfactant **AzoTAB** seems to possess a non-selective mode of antibacterial action toward these bacteria, the type of head group and the isomeric state of the tail group in carbohydrate-based surfactants are both important determinants for imparting selective growth/toxicity against different bacteria.

### Photocontrollable biofilm studies

Having established the bacteria-specific influence of these surfactants on cell viability, we next sought to evaluate the effect of *trans*–*cis* photoisomerization on their ability to influence bacterial biofilm formation. Biofilms are typically formed by adherence of sessile bacteria onto a surface followed by the production of a complex extracellular matrix, thus allowing these bacterial communities to become more resistant to host immune responses and antibiotic therapies. Not only are the mechanisms of biofilm formation poorly understood, the ability to inhibit biofilm formation from pathogenic bacteria is extremely challenging.^26^ The intriguing effect of amphiphilic carbohydrates on biofilm growth of *P. aeruginosa* has been recently demonstrated and was shown to be heavily dependent on the length and relative configuration of the oligosaccharide head group (d-*gluco* or d-*galacto*).^6^ However, the influence of different photoisomers of amphiphilic carbohydrates on the biofilm forming ability of bacteria has never been reported. To understand the influence of the *trans* and *cis* isomers of these surfactants on the biofilm forming ability of bacteria, these compounds were exposed to a Gram-positive methicillin-resistant *S. aureus* (MRSA – ATCC1698) and a multi-drug resistant clinical isolate of Gram-negative *P. aeruginosa* (MDR283/1-6), both of which are known to cause problematic biofilm-associated infections.^2,27^

**Fig. 5:**
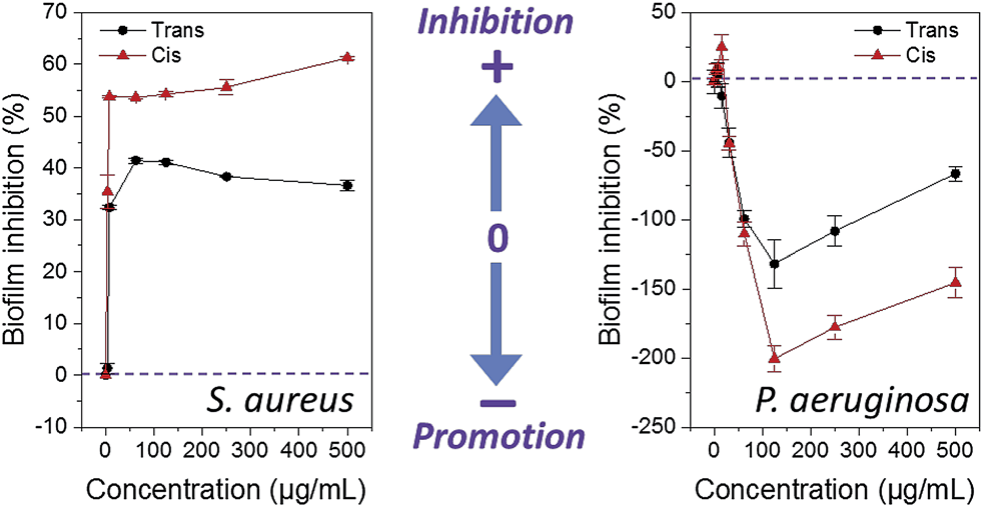
Influence of *cis*- and *trans*-dominated photostationary states of **AzoMan** on biofilm formation of *S. aureus* and *P. aeruginosa*.

Compounds were screened for biofilm inhibition activity using a microtiter dish biofilm formation assay that involved recording the OD_550_ of crystal violet-treated bacterial biofilms.^28^ Similar to the antibacterial studies, low concentrations of surfactants showed similar effects in both *cis* and *trans*-dominated PSS, whereas the difference in activity between the photoisomeric states became more pronounced at higher concentrations (representative data for compound **AzoMan** is shown in Fig. 5; additional data for surfactants are shown in ESI Fig. S10 and S11†). In line with the antibacterial data, these surfactants generally compromised the biofilm forming ability of Gram-positive *S. aureus* at all concentrations tested, and conversely promoted biofilm formation of Gram-negative MDR *P. aeruginosa*, particularly on exposure to higher concentrations of these compounds. In the case of *S. aureus*, **AzoXyl**, **AzoMan**, and **AzoGlcNAc** could inhibit bacterial biofilms more efficiently after photoexcitation, whereas the native *trans* states of **AzoGlc**, **AzoRha** and **AzoAra** remained more potent biofilm inhibitors. Similar to the antibacterial results, **AzoRha** revealed an interesting trend, such that lower concentrations of this compound in both the *cis* and *trans* PSS inhibited *S. aureus* biofilm formation, whereas higher concentrations of the *cis* isomer started promoting biofilm formation. Another notable observation was that while **AzoGlcNAc** displayed selective antibacterial activity against *S. aureus* in the *trans* form, the *cis*-dominated photostationary state was more effective at inhibiting biofilm growth of this bacterium, suggesting a mechanism that was independent of cell death.^29^

### Bacterial motility studies

Bacterial motility is an important physiological process that enables bacteria to seek new environments and nutrients, and as such, plays important roles in the pathogenesis as well as biofilm growth, dispersal, structure and function.^30^ Many bacterial species produce biosurfactants in response to environmental and internal cues, which results in a decrease in interfacial tension that enhances cellular motility and along surfaces and in solution. To help better understand the intriguing biofilm modulatory activity of the surfactants, we decided to probe whether these compounds were affecting bacterial motility in solution and swarming motility on a semi-solid surface. We measured the dynamics and rates of swimming motility using differential dynamic microscopy (DDM) in the presence of representative **AzoRha** and **AzoMan**. DDM is a technique that uses optical microscopy to conduct an analysis similar to that used in dynamic light scattering. It has been shown that the technique is ideal for the measurement of dynamics and rates of motility of cells in bulk solution (‘swimming’).^31^ To this end, *P. aeruginosa* MDR283/1-6 was chosen as the model organism as it shows sufficiently fast rates of swimming required for the timescales measured using DDM. Using this technique, these photosurfactants did not appear to significantly affect the rate of swimming in solution up to concentrations of 500 μg mL^–1^, in both the *cis*- and *trans*-dominated PSS (ESI, Fig. S12†). Based on this observation, it is therefore unlikely that these compounds are effecting biofilm growth through changes in the swimming motility of this bacterial species.

In the case of *P. aeruginosa*, compounds were shown to weakly inhibit biofilm growth at low concentrations and strongly promote biofilm growth at higher concentrations (Fig. 5). The photoexcited *cis* isomers were typically more or at least equally effective over their native forms in promoting biofilms of *P. aeruginosa.* Given the important roles that rhamnolipid biosurfactants play in promoting swarming motility, we decided to investigate whether the *cis* and *trans* forms of the surfactants were affecting swarming motility of *P. aeruginosa* MDR283/1-6 *via* photocontrollable changes in the interfacial activity. Swarming motility of *P. aeruginosa* was assessed in 0.5% agar media in the presence of the *trans* and *cis* isomers these compounds, along with controls using a previously described swarming assay (see ESI for details†).^32^

**Fig. 6:**
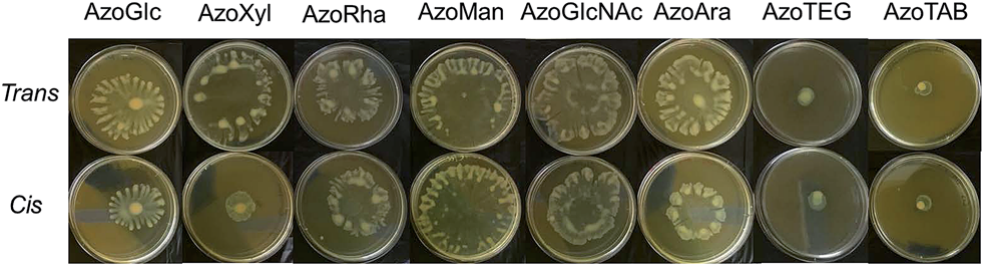
Photograph of agar swarming motility assay demonstrating the influence of carbohydrate-based surfactants and control compounds in the *cis*- and *trans*-dominated PSS on the swarming motility of *P. aeruginosa* MDR283/1-6. Cells were inoculated at the centre of the agar media containing compound (100 μg mL^–1^) and incubated at 30 °C for 24 h.

In the native *trans* form, surfactants displayed a dose-dependent enhancement in swarming motility (Fig. 6). The response was insensitive to the head group employed, with all compounds being more-or-less equally effective at promoting swarming motility. However, in the *cis* photoisomeric state, the surfactants were generally less effective at promoting swarming motility, which may be a direct consequence of the lowered interfacial activity of the *cis* isomer relative to the *trans* isomer (ESI, Fig. S13†). Interestingly, in contrast to the surfactants bearing a non-ionic carbohydrate head group, which generally promoted swarming motility, the non-surface active tail fragment **AzoTEG** and the cationic surfactant **AzoTAB** strongly inhibited swarming motility. Furthermore, unlike the carbohydrate-based surfactants, there were no significant changes in the inhibitory activity of **AzoTEG** and **AzoTAB** following *trans*–*cis* photoisomerization.

**Fig. 7:**
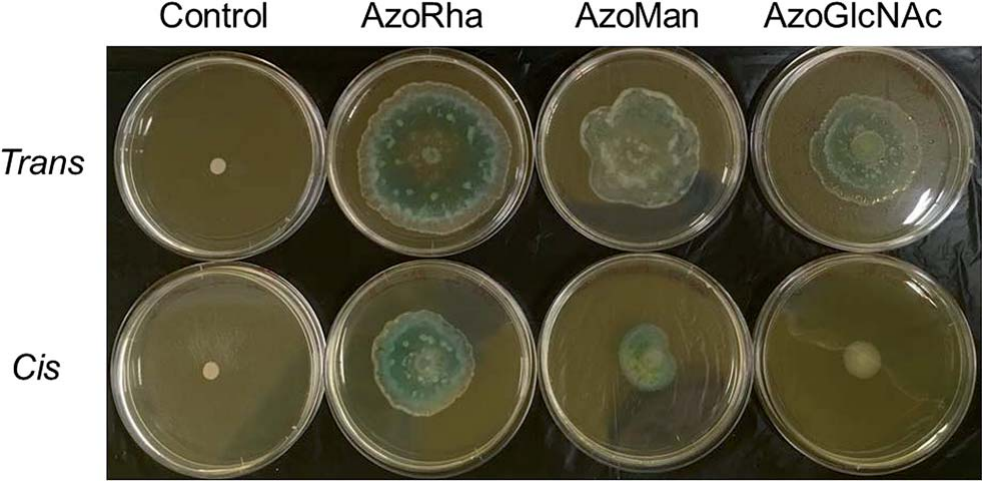
Photograph of agar swarming motility assay demonstrating the influence of carbohydrate-based surfactants (25 μg mL^–1^) in the *cis*- and *trans*-dominated PSS on the swarming motility of *P. aeruginosa* MDR283/1-23, a swarming negative strain.

To validate the strong influence of our carbohydrate surfactants on swarming motility of *P. aeruginosa* MDR283/1-6 (a swarming-positive strain), we further investigated the influence of surfactants on the swarming motility of *P. aeruginosa* MDR283/1-23, a swarming-negative strain (Fig. 7). Remarkably, while untreated bacteria showed no detectable motility, these carbohydrate-based surfactants strongly promoted translocation of the bacterial colony, most likely through enhancement of surfactant-mediated sliding.^33^ Similar to the swarming positive strain of *P. aeruginosa*, the *trans* configured surfactants promoted a higher level of motility relative to their *cis* isomers, particularly for **AzoGlcNAc**, which displays the largest difference in CMC between the respective *trans* and *cis* isomeric state. Taken together, these observations demonstrate the utility of UV light for modulating the swarming motility and biofilm forming ability of *P. aeruginosa* strains, which may potentially be a result of the associated changes in the interfacial activity of the *cis* and *trans* isomers of photoswitchable carbohydrate-based surfactants.

## Conclusions

In summary, we report the modular synthesis and characterisation of a panel of novel carbohydrate-based surfactants and demonstrate unprecedented photo-control over bacterial growth, biofilm formation and motility using photoswitchable surfactants. Using SANS, we revealed the stark difference in the self-assembly properties of these amphiphiles, which is made possible through structural and stereochemical modification of the carbohydrate head group. Photocontrollable surface tension studies also revealed dramatic differences in the interfacial properties, which could be tuned using UV light irradiation. We then probed the impact of *trans*–*cis* azobenzene photoisomerization of these amphiphiles on their ability to modulate the growth and formation of biofilms from pathogenic, drug-resistant strains of *S. aureus* and *P. aeruginosa*. Photoswitchable carbohydrate-based surfactants displayed dose-dependent, as well as bacteria- and photo-isomer specific biological activity, which suggests that these amphiphiles possess a selective mode of action. Interestingly, the photoexcited *cis* forms of these surfactants was shown to be more potent against *S. aureus*, while the native *trans* state showed higher selectivity against *E. coli*. Surprisingly, whilst selective antibacterial activity could be observed against *E. coli* and *S. aureus*, these compounds strongly promoted the growth of Gram-negative *P. aeruginosa* MDR283/1-6, despite the well-documented antimicrobial activity of azobenzene dyes toward *Pseudomonas* spp. and other Gram-negative bacteria. Furthermore, the reduction in inhibitory potency of some surfactants at higher concentrations may suggest an antagonistic affect owing to impaired uptake of surfactant monomers and/or their metabolites due to micellization.^34^ Carbohydrate-based surfactants also showed remarkable bacteria-specific control over biofilm formation, such that they showed anti-biofilm activity against *S. aureus*, whereas they promoted biofilm formation in *P. aeruginosa*, with strong preference for the *cis* isomer. We further investigated the possible mechanisms governing the intriguing biofilm modulatory activity of these photosurfactants by measuring the rates of swimming and swarming motility of *P. aeruginosa.* Whilst the rates of swimming motility were generally insensitive to the *trans* and *cis* isomers of these compounds, surfactants were shown to strongly promote swarming motility in *P. aeruginosa* MDR283/1-6. We also demonstrated the ability of these compounds to promote the motility of the non-motile strain *P. aeruginosa* MDR283/1-23 on semi-solid agar, which could be further tuned using UV light. Through *trans*–*cis* photoisomerization of the azobenzene tail group, a decrease in bacterial motility could be observed relative to the *trans* isomer, presumably through reduction in the interfacial activity of the corresponding *cis* isomer. Overall, these results demonstrate the utility of UV-visible light for modulating the physicochemical properties of surfactants in order to control the physiological behaviour of bacteria. Further work is underway to understand the mechanistic interaction of the *trans*- and *cis*-photoisomeric states bearing different head groups with bacteria and their extracellular matrices, including lectin-specific inhibitors of bacterial adhesion and fluorescent uptake studies, as well as the development of dual visible light near infrared photoswitchable surfactants for *in vivo* applications.
