# Supplementary material for: Photomodulation of bacterial growth and biofilm formation using carbohydrate-based surfactants
Source: Chem Sci. 2016 Aug 17;7(11):6628–34. doi: 10.1039/c6sc03020c (PMC5450525; doi:10.1039/c6sc03020c)
Supplement: Supplementary file 1 [file SC-007-C6SC03020C-s001.pdf]

Electronic Supplementary Material (ESI) for Chemical Science.
This journal is © The Royal Society of Chemistry 2016

Supporting Information

Photomodulation of bacterial growth and biofilm formation using carbohydrate-based surfactants

Yingxue Hu,[a†] Wenyue Zou[b†], Villy Julita[a], Rajesh Ramanathan[b], Rico F. Tabor[a], Reece Nixon-Luke,[c] Gary Bryant,[c] Vipul Bansal[b]* and Brendan L. Wilkinson.[d]*

a) School of Chemistry, Monash University, Victoria 3800, Australia

b) Ian Potter NanoBioSensing Facility, NanoBiotechnology Research Laboratory, School of Sciences, RMIT University, PO Box 2476, Melbourne 3001, Australia

c) Centre for Molecular and Nanoscale Physics, School of Science, RMIT University, Victoria 3000, Australia

d) School of Science and Technology, The University of New England, New South Wales 2351, Australia

Contents

1.0 Materials and Methods **S3**
General S3
UV-Vis stability studies on *cis* isomers S4
Surface tension measurements S11
Small angle neutron scattering (SANS) S13
Antibacterial assays S15
Minimum inhibitory concentration (MIC) test S19
Biofilm assays S19
Differential dynamic microscopy (DDM) S23
Swarming motility assays S25
2.0 Synthesis and analytical data **S27**
2-[2-[2-(4-n-butylazophenyl phenoxy)ethoxy)ethoxy]ethoxy]methyl β-D-xylopyranosyl-1,2,3-triazole (**AzoXyl**) S27
2-[2-[2-(4-n-butylazophenyl phenoxy)ethoxy)ethoxy]ethoxy]methyl α-L-rhamnopyranosyl-1,2,3-triazole (**AzoRha**) S28
2-[2-[2-(4-n-butylazophenyl phenoxy)ethoxy)ethoxy]ethoxy]methyl α-D-mannopyranosyl-1,2,3-triazole (**AzoMan**) S29
2-[2-[2-(4-n-butylazophenyl phenoxy)ethoxy)ethoxy]ethoxy]methyl 2-acetamido-2-deoxy-β-D-glucopyranosyl-1,2,3-triazole (**AzoGlcNAc**) S30
2-[2-[2-(4-n-butylazophenyl phenoxy)ethoxy)ethoxy]ethoxy]methyl α -L-arabinopyranosyl-1,2,3-triazole (**AzoAra**) S30
3.0 References **S32**

1.0 Materials and Methods

General

Analytical thin layer chromatography (TLC) was performed on commercially prepared silica plates (Merck Kieselgel 60 0.25 mm F254). Flash column chromatography was performed using 230-400 mesh Kieselgel 60 silica eluting with distilled solvents as described. Solvents and reagents were purchased from Sigma-Aldrich and Merck and used without further purification. ^{1}H NMR and ^{13}C NMR spectra were recorded on a Bruker Avance 400 NMR spectrometer at frequencies of 400 MHz and 100 MHz respectively. Chemical shift is reported as parts per million (ppm) downfield shift. The data are reported as chemical shift (δ), multiplicity, relative integral, coupling constant (*J* = Hz) and assignment where possible. IR spectra were recorded on a Bruker ATR spectrometer. Optical rotation was measured on an Optical Activity Polaer 2001 (546 nm) polarimeter using a 1 mL cell.

LC-MS was recorded on an Agilent 6120 LC-MS system operating in positive ion mode. Separations were performed on an Agilent Poroshell-120 2.7 µm(3.0 mm x 50 mm) C18 column using a linear gradient of 0.1% formic acid in water (Solvent A) and 0.1% formic acid in acetonitrile (Solvent B) as the mobile phase. Separations were performed using a linear gradient of 20% solvent B to 100% solvent B over 15 minutes, operating at a flow rate of 0.3 mL/min.

Deprotected carbohydrate-based surfactants were purified by reversed-phase (C18) preparative HPLC using an Agilent 1260 preparative HPLC system equipped with an automated fraction collector. Separations were performed on an Agilent Zorbax SB300 5 µm (20 mm x 150 mm) C18 column using a linear gradient of 0.1% formic acid in water (Solvent A) and 0.1% formic acid in acetonitrile (Solvent B) as mobile phase, operating at a flow rate of 10 mL/min. Carbohydrate-based surfactants were purified using a linear gradient of 20% solvent B to 100% solvent B over 40 minutes (monitoring at 280 nm).Purified fractions were subsequently combined and lyophilized.

Nutrient Broth (NB), Brain Heart Infusion (BHI) broth, Tryptic Soy Broth (TSB), tryptone, yeast extract, Mueller- Hinton Broth (MHB) and agar were purchased from Amyl Media PTY LTD (NB); Becton, Dickinson and Company (BHI); Oxoid PTY LTD (TSB, tryptone, yeast extract and MHB) and Sigma-Aldrich (agar). All media components were used as received. *Escherichia coli* (DH5α) and methicillin-resistant *Staphylococcus aureus* (MRSA ATCC 1698) were procured from Southern Biologicals and maintained on Nutrient agar plates, as per standard microbiological protocol. Two multi-drug resistant *Pseudomonas aeruginosa* strains (MDR283/1-6 and MDR283/1-23), previously isolated from the clinical samples were maintained on NA plates and used for biofilm assays and swarming motility assays. Crystal violet used in biofilm assays was purchasedfrom E. Merck (Darmstadt, Germany). Glucose used in swarming motility assays was from Sigma.

Photoswitching experiments were performed using a UV light source which was comprised of four 9 Watt halogen tubes, delivering a total power of 36 W at λ_{max} of 361 nm at the source (Nail curing lamp, eBay). The working distance between the source and the centre of the samples for the photo-modulated biological studies was fixed at 4.5 cm. UV-vis spectra were recorded in 96-well format using a Perkin Elmer EnVision^{TM} 2104 Multilabel Plate Reader (instrument linear operating range 0-4 OD; see antibacterial assay section for more details).

UV-Vis stability studies on *cis* isomers

The native *trans* isomers of carbohydrate surfactants were converted into *cis* states by illumination of their aqueous solutions under ambient conditions using a UV lamp with λ_{max} at 361 nm in a time-dependant manner (1–90 min). One minute of photoexcitation was found sufficient to convert *trans* isomers into *cis* state. All the studies involving bacteria utilised *cis* isomers obtained after 5 min of illumination with λ_{361nm}. Since bacterial experiments were performed at 37 °C, the thermal stability of both the native *trans* isomer as well as the photoinduced *cis* isomer was studied at 37 °C using UV-visible spectroscopy. Azobenzene *trans*-*cis* photoisomerisation was found to be complete within 1 minute of UV irradiation. The native *trans* isomers remained stable under ambient lighting conditions at least up to 24 h, as evident from insignificant changes in their UV-vis absorbance spectra over this period. The thermal relaxation

studies were performed at 37 °C for 24 h in deionised water as well as in the growth media employed for the antibacterial studies against *E. coli* (NB) and *S. aureus* (BHI). The photoinduced *cis* isomers were found to thermally relax back to their native configuration within 24 h at 37 °C in deionized water, NB and BHI media and the data is summarized in Table S1. The half-lives of the *cis* isomers in water and two bacterial media were estimated from thermal relaxation studies by plotting the ratios of peak area under 350 nm peak and that under 440 nm peak over a period of 24 h. These peaks were chosen as *cis-trans* relaxation results in increase in peak intensity at ~350 nm, with a corresponding decrease in peak intensity at 440 nm.

Table S1. Half-lives (h) of *cis* isomers of surfactants (0.1 mM) in water, NB and BHI at 37 °C.

Surfactant	***Water***	***NB***	***BHI***
**AzoGlc**	11.49	10.63	11.02
**AzoXyl**	12.58	12.18	11.40
**AzoRha**	11.16	10.42	10.64
**AzoMan**	12.02	10.88	11.01
**AzoGlcNAc**	10.31	10.89	10.95
**AzoAra**	10.01	10.54	11.18

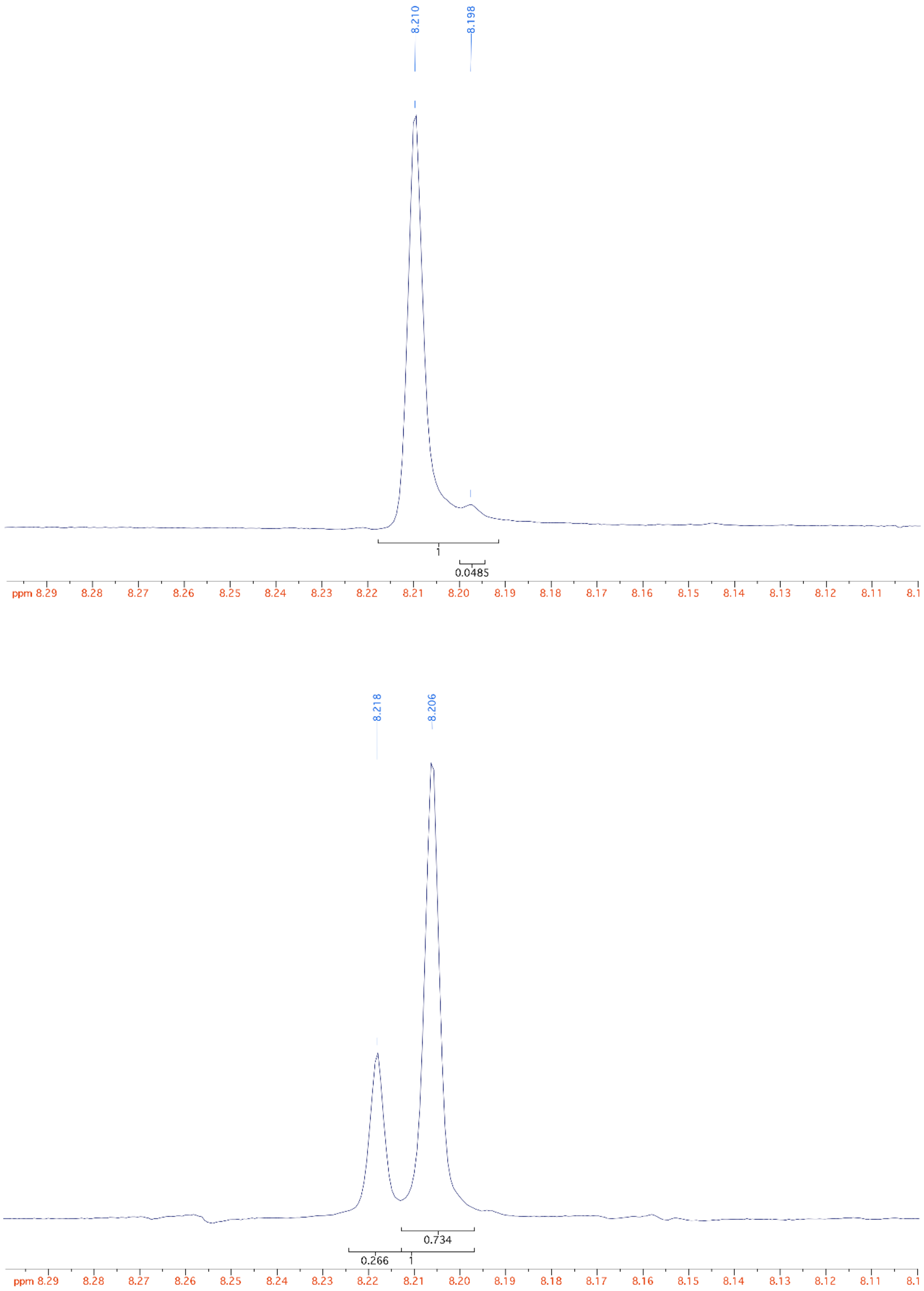

Figure S1. ^{1}H NMR spectra of previously described **AzoGlc** surfactant (triazole proton)[1] in the *trans*-dominated PSS (top), and B) the *cis*-dominated PSS (bottom).

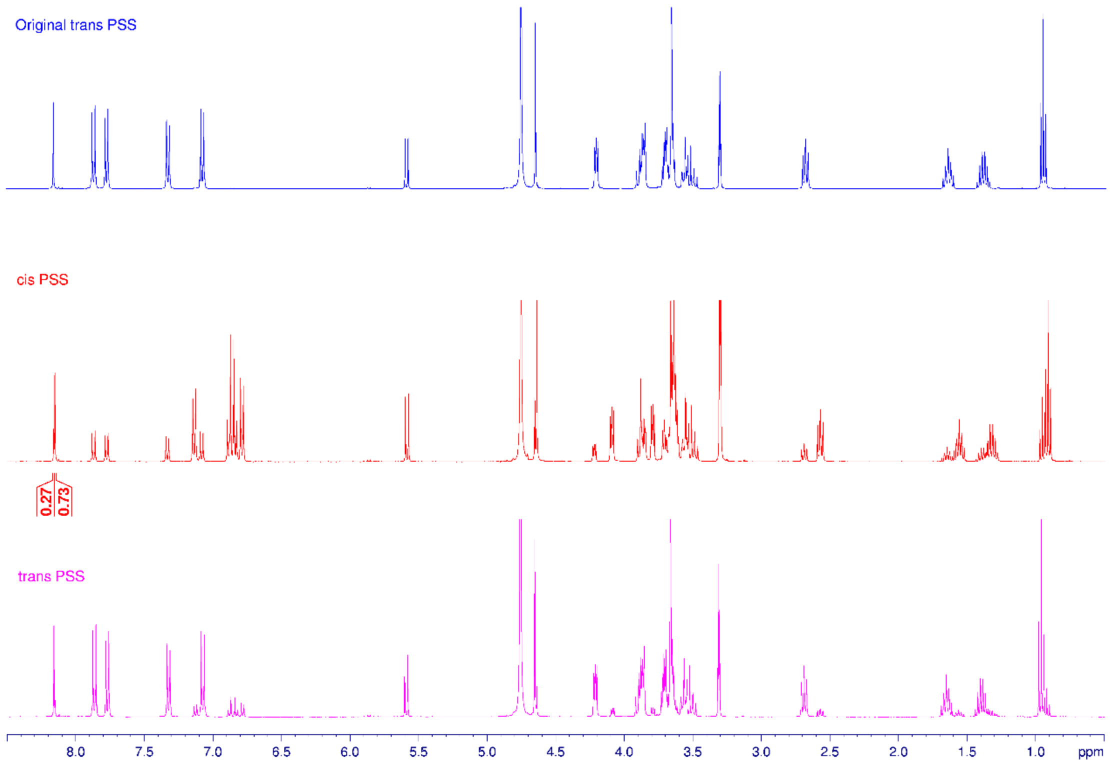

Figure S2. ^{1}H NMR spectrum of **AzoGlc** before UV photoirradiation (top), after UV photoirradiation (middle), and after visible (blue) light photoirradiation (bottom). Spectra were recorded in CD_3OD at 20 mM concentration. The original *trans* dominated PPS of **1** was irradiated with UV light (365 nm, 6.5 W) for 30 min. The resulting *cis* dominated PSS of **1** was the irradiated with blue light (440 nm, 3W) for 30 min in order to photoswitch back to the *trans*-dominated PSS. Approximately 90% of the *cis* isomer was still present, although no photodegradation by-product was observed.

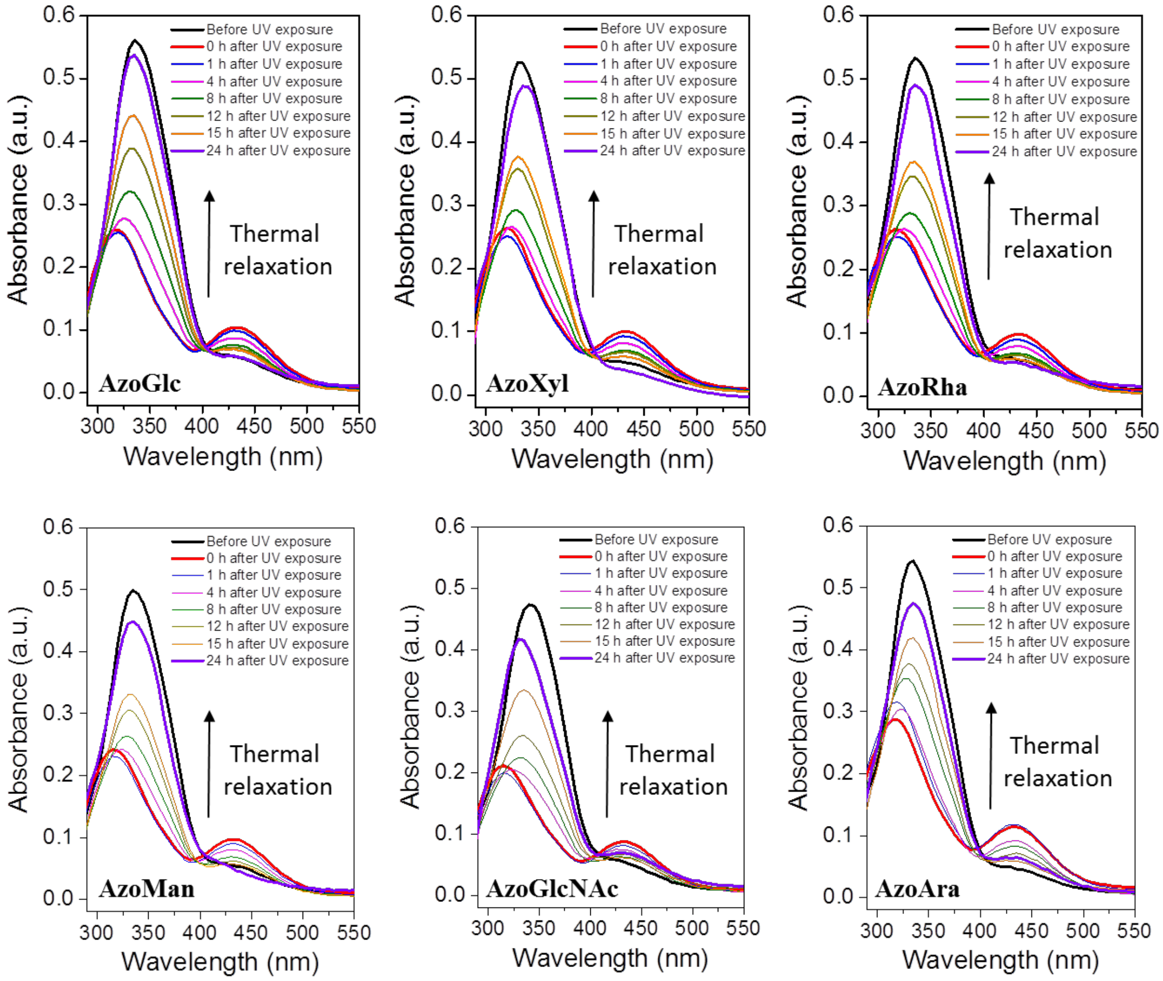

Figure S3. Time-dependent relaxation of the photoinduced *cis* isomers of surfactants to their native *trans* isomers at 37 °C in water (0.1 mM).

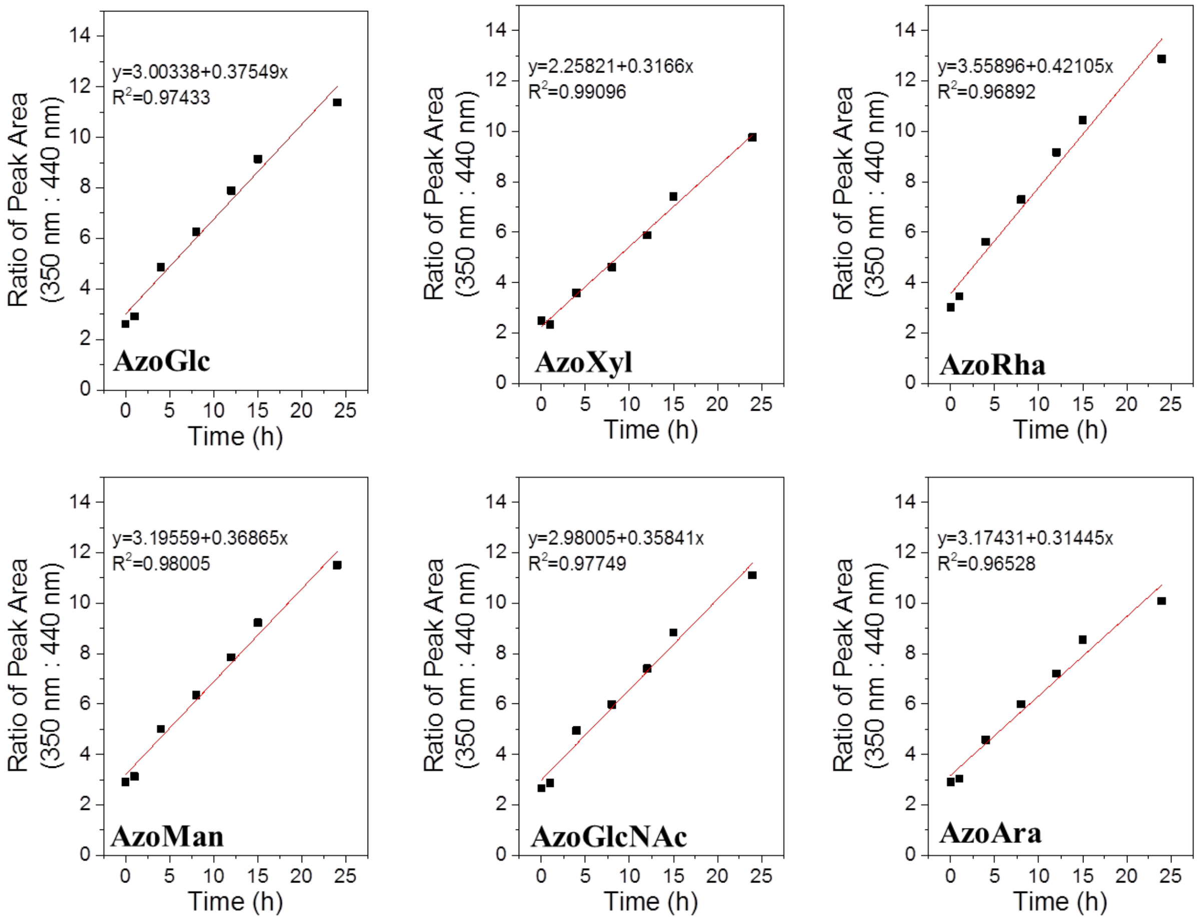

Figure S4. Ratios of absorbance of surfactants (0.1 mM) at 350 and 440 nm during time-dependent thermal relaxation of photoinduced *cis* isomers to their native *trans* isomers at 37 °C in nutrient broth (NB). The half-lives of *cis* isomers calculated from these graphs in NB at 37 °C are indicated in Table S1.

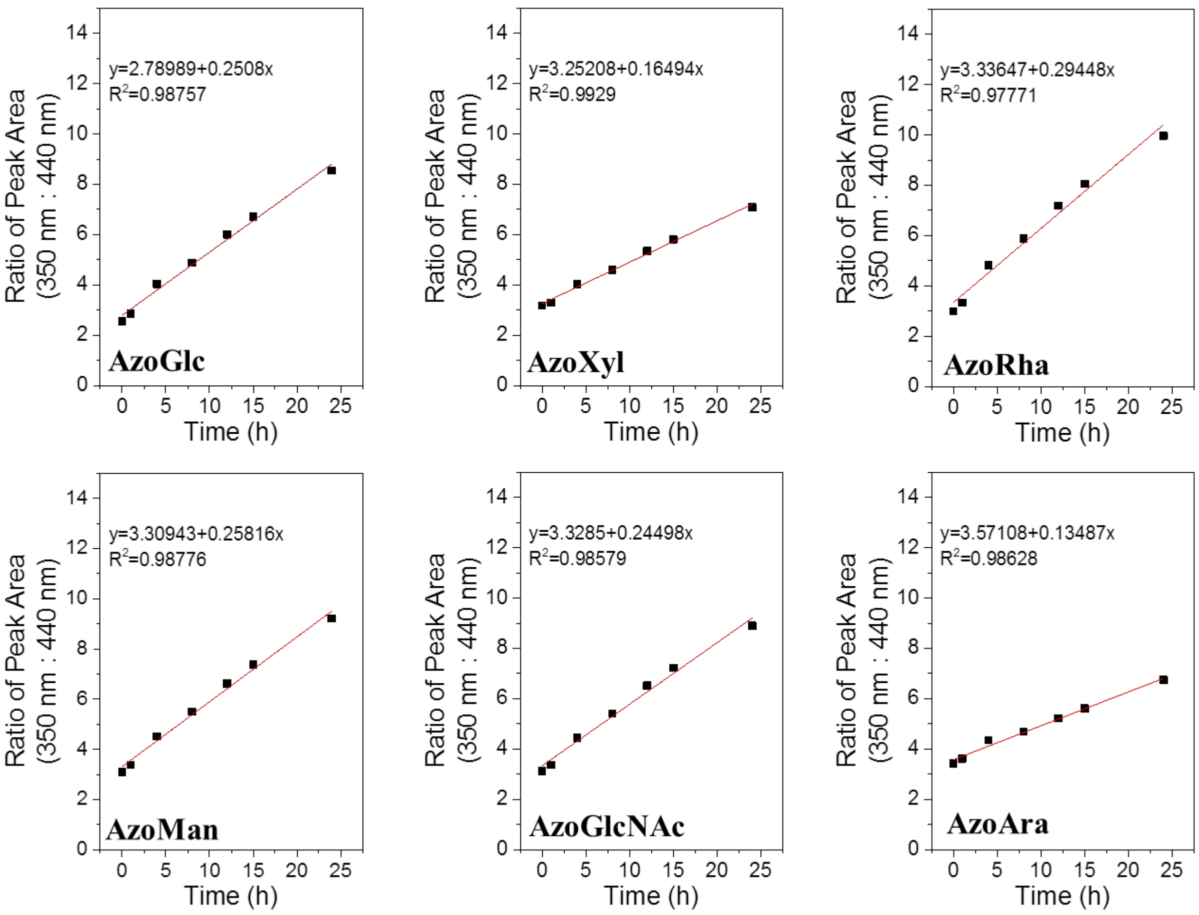

Figure S5. Ratios of absorbance of surfactants (0.1 mM) at 350 and 440 nm during time-dependent thermal relaxation of photoexcited *cis* isomers to their native *trans* isomers at 37 °C in brain heart infusion broth (BHI). The half-lives of *cis* isomers calculated from these graphs in BHI at 37 °C are indicated in Table S1.

Surface tension measurements

Surface tension measurements were made on a custom-designed pendant drop instrument.[1] A time series was taken (that is, surface tension as a function of time) and values were noted for 100 s to ensure full equilibration of interfacial adsorption. Once a stable surface tension had been attained, this was recorded. Drop volumes were measured throughout and changes of <5% throughout the course of a measurement were a requirement for the data shown. Critical micelle concentration (CMC) values were obtained from the intersection of lines extrapolated from surface tension values in the near pre- and post-CMC regions. Surface tension data for surfactants not shown in the main paper is provided in Figure S6.

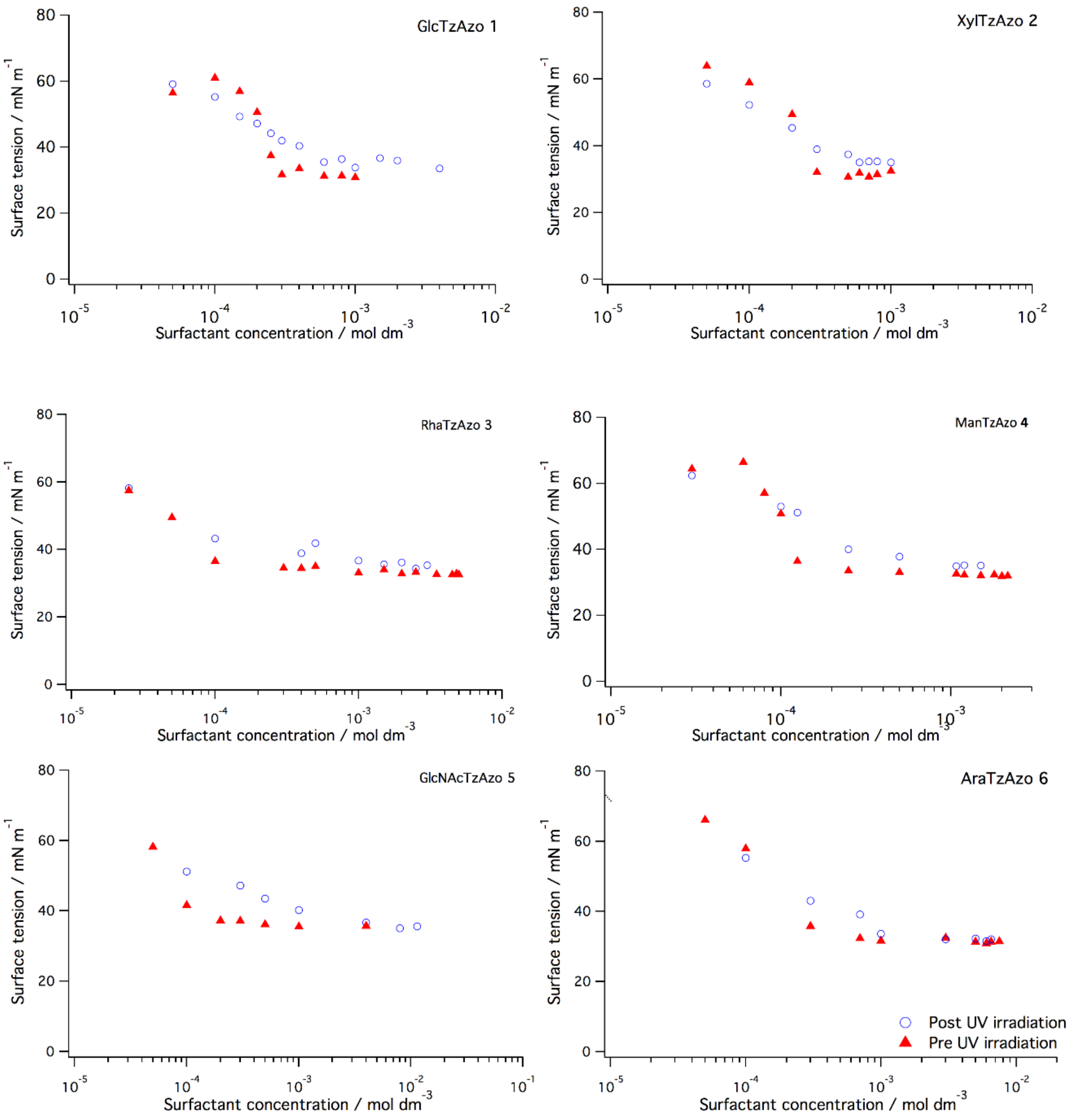

Figure S6. Photocontrollable surface tension data for photosurfactants. The surface tension data for **AzoGlc** has been published previously.[1]

Small angle neutron scattering (SANS)

SANS measurements were made on the Quokka instrument at the Bragg Institute, ANSTO, Lucas Heights NSW, Australia. For all samples, raw scattering counts were collected on a 128 x 128 element area detector, where the sample- detector distances used were 2 m and 14 m, with no detector off-set. An incident neutron wavelength of 5 Å was used with a typical spread of 10%, thus giving an effective q-range of 0.004 – 0.4 $Å^{-1}$. Samples were prepared in circular 12.5 mm Hellma quartz cells with a path-length of 2 mm, and a thermostatically-controlled automatic sample changer ensured that a temperature of 25 +/– 0.05°C was maintained. Data were converted from raw counts at the detector into 1D scattering spectra by first subtracting the scattering from an empty cell and then radially averaging the resulting spectrum, normalising for the measured sample transmission. A D_2O background was then subtracted from the final 1D sample data to ensure that the scattering signals seen are from the surfactant only. All samples were run at a fixed concentration of 4 mM, well above the CMC for each. Fitting was performed using standard equations for ellipsoidal and cylindrical form factors as described previously.[1,2] Experimental and fitted SANS spectra are shown in Table S2.

Table S2. Photocontrollable surface and aggregation properties of photoswitchable carbohydrate-based surfactants.[a]

Surfactant	Geometry	Radius/R_a nm	Radius (length)/R_b nm	N_{agg}	A_{hg/nm^2}
**AzoXyl**	C*	2.8457	822.8	NA	NA
**AzoRha**	C	2.5827	27.62	622.3375	0.7873
**AzoMan**	E	2.2787	6.032	140.8755	0.8442
**AzoGlcNAc**	E	8.2609	46.40	177.6041	1.0166
**AzoAra**	E	1.9942	4.767	88.81305	1.1225

[a]Surfacants were prepared at 4 mM concentration in D_2O. Aggregation number (N_{agg}), area per head group (A_{hg}) [nm^2] and critical micelle concentration (CMC) in ambient and UV-irradiated conditions [mm]. Other samples were analyzed at neutral pH. [b] E = ellipsoid, C = cylindrical.
*Flexible cylinder (kuhn length 36.364nm).

Antibacterial assays

The antibacterial activity of these compounds was tested against Gram-negative *E. coli,* Gram-positive *S. aureus* and Gram-negative *P. aeruginosa* using an OD_{600} method.[3] A fixed concentration (OD_{600} – 0.1: log phase 10^8 CFU/mL) of freshly grown bacterial cultures were used for antibacterial assay, after determining the bacterial concentration using a GENESYS 10S UV-Vis Spectrophotometer. 100 µL of the bacterial suspension (*E. coli* in NB and *S. aureus* in BHI broth) was incubated with 100 µL of photosurfactants at 37 °C in dark for 24 h. Different concentrations of photosurfactants (0, 1, 10, 25, 50, 100, 250 and 500 µg/mL) were used to determine the concentration-dependent influence on bacterial growth. Appropriate controls were chosen wherein one of the controls contained no surfactants while the other contained a mixture of different concentrations of photosurfactants with the bacteria-free growth media. The bacterial growth curves were monitored at OD_{600} after 24 h in 96-well format using a Perkin Elmer EnVision™ 2104 Multilabel Plate Reader (instrument linear operating range 0-4 OD). All antibacterial assays were performed in triplicates; tests were repeated independently three times; each well was read five times. The average of 45 readings (3 x 3 x 5) in each case was calculated and plotted along with standard deviation. The data was background corrected by subtracting the OD_{600} value obtained from the control containing a mixture of photosurfactants and the bacteria-free media growth from all other samples (Figure S7-S9).

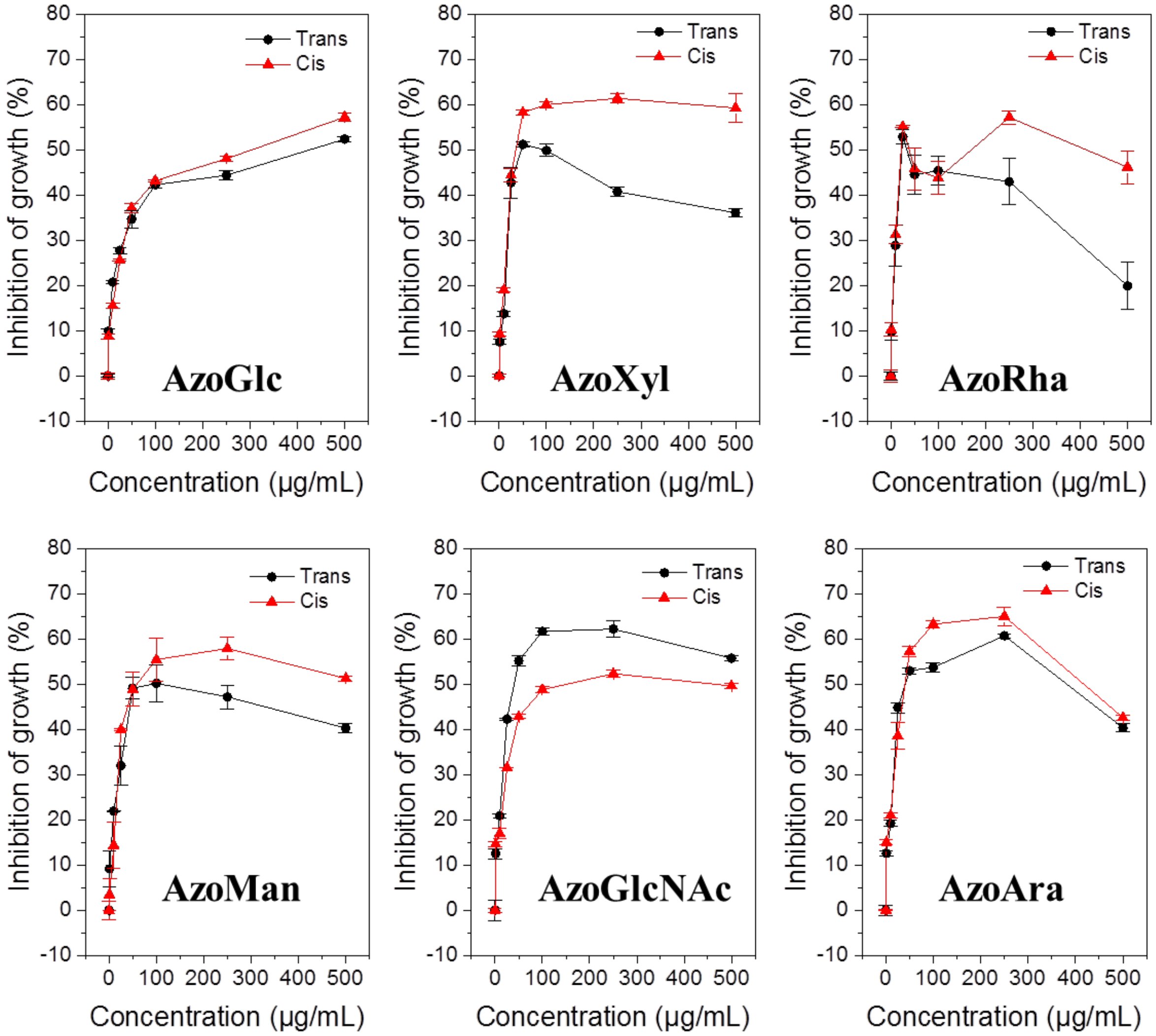

Figure S7. Concentration-dependent antibacterial profile of *trans* and cis isomers of photosurfactants against *S. aureus* at 37 °C in brain heart infusion broth (BHI) after 24 h of incubation with the respective carbohydrate surfactants.

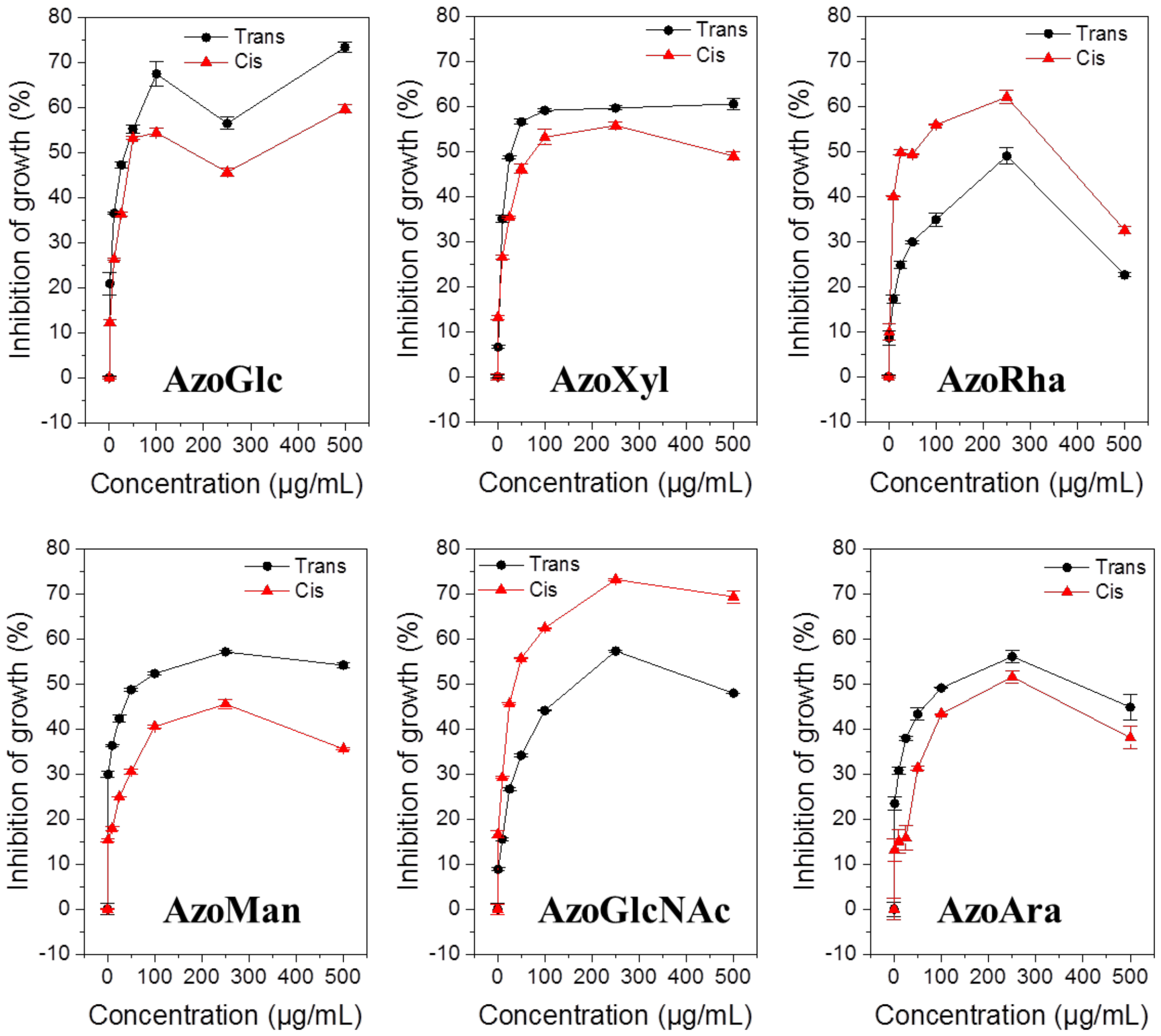

Figure S8. Concentration-dependent antibacterial profile of *trans* and cis isomers of photosurfactants against *E. coli* at 37 °C in nutrient broth (NB) after 24 h of incubation with the respective carbohydrate surfactants.

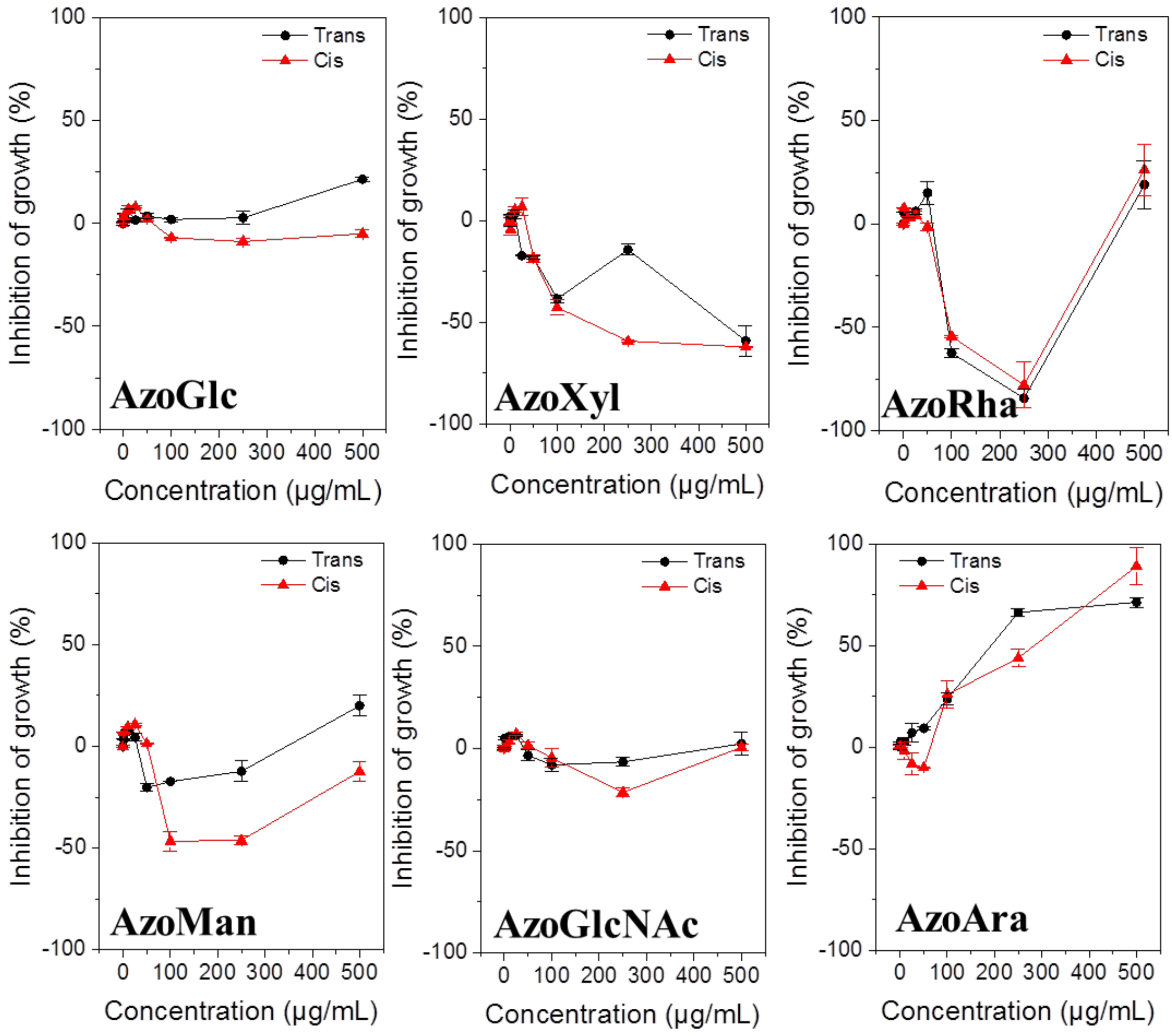

Figure S9. Concentration-dependent antibacterial profile of *trans* and cis isomers of photosurfactants against *P. aeruginosa* at 37 °C in nutrient broth (NB) after 24 h of incubation with the respective carbohydrate surfactants.

Minimum inhibitory concentration (MIC) test

The antibacterial activity of **AzoTAB** and non-surface active **AzoTEG** were tested against Gram-negative *E. coli* and *P. aeruginosa* andGram-positive *S. aureus* using standard MIC test.[4] A fixed concentration of the compound in Mueller-Hinton broth was dispensed in volumes of 100 µL per well in round bottom 96-well microdilution plates. Inocula were prepared by suspending *E. coli* or *P. aeruginosa* grown on NA plate or *S. aureus* grown on BHI agar plate for 18 h in saline (0.85% w/v NaCl in water) to match the turbidity of a 0.5 McFarland standard. This suspension was further diluted to provide a final inoculum density of 5×10^5 CFU/mL in the wells of the 96-well plates. Appropriate controls were chosen wherein one of the controls contained only bacteria without surfactants (positive control) while the other contained only media without surfactants or bacteria (negative control). After incubation at 37 °C for 24 h in dark, plates were studied for visual turbidity, and MIC was defined as the lowest concentration at which bacterial growth (turbidity) could not be visibly observed by the naked eye. All MIC tests were performed in triplicates and repeated independently three times.

Biofilm assays

The photoswitchable surfactants and control compounds were screened for their influence on biofilm forming ability of a Gram-positive methicillin-resistant *S. aureus* and a multi-drug resistant Gram-negative *P. aeruginosa*. The amount of biofilm formation in 96-well plates was determined by a microtiter dish method which allows for the formation of a biofilm on the wall and/or bottom of a microtiter dish.[5] A fixed concentration of overnight grown bacterial cultures were used for biofilm assays after 1:100 dilution of the overnight-grown bacteria culture in a in a fresh broth (BHI broth for *S. aureus* and TSB for *P. aeruginosa*) containing different concentrations of the photosurfactants. Appropriate controls were chosen wherein one of the controls contained only bacteria without surfactants (positive control) while the other contained neither bacteria nor surfactant (blank). The samples (100 µL) were incubated in 96-well tissue culture plates in dark at 37 °C for 24 h to allow biofilm formation, following which the plates were gently washed 3 times with water to remove unattached bacterial cells and media components. The plates were then kept at 55 °C for 1 h to rigidly fix the biofilms to the plates, followed by staining the biofilms with 125 µL crystal violet (0.1% w/v in water) at room temperature for 10 min. The plates were then exposed to running tap water until free crystal violet stops releasing from the biofilms. The biofilms were then dried overnight in air, followed

by addition of 125 µL of acetic acid (30% v/v in water) to each well to solubilize the crystal violet taken up by the bacterial biofilms. The solubilized crystal violet was monitored at OD_{550} using a Perkin Elmer EnVision™ 2104 Multilabel Plate Reader. All biofilm assays were performed in triplicates; tests were repeated independently three times; each well was read five times. The average of 45 readings (3 x 3 x 5) in each case was calculated and plotted along with standard deviation. The data was background corrected by subtracting the OD_{550} value obtained from the blank control from all other samples (Figure S10 and S11).

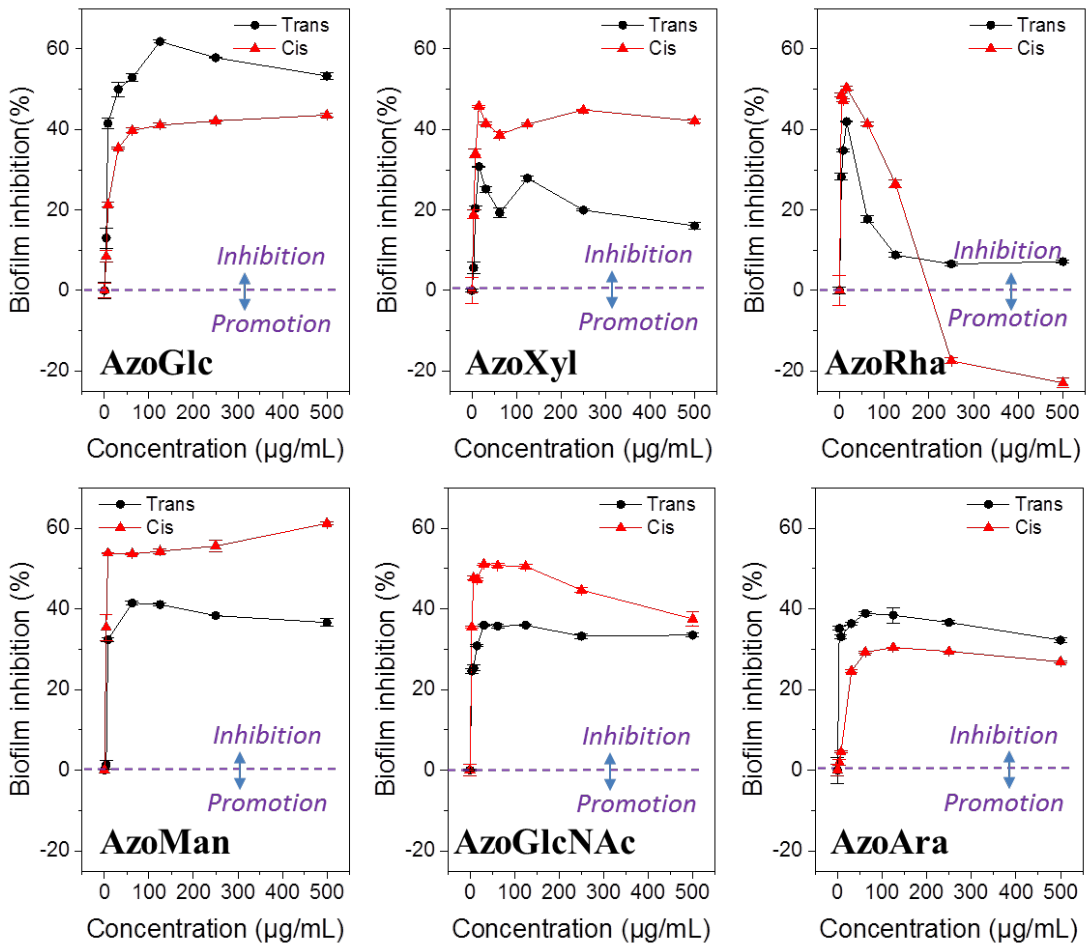

Figure S10. Concentration-dependent influence of *trans* and cis photoisomers of surfactants on biofilm forming ability of *S. aureus* at 37 °C after 24 h of exposure.

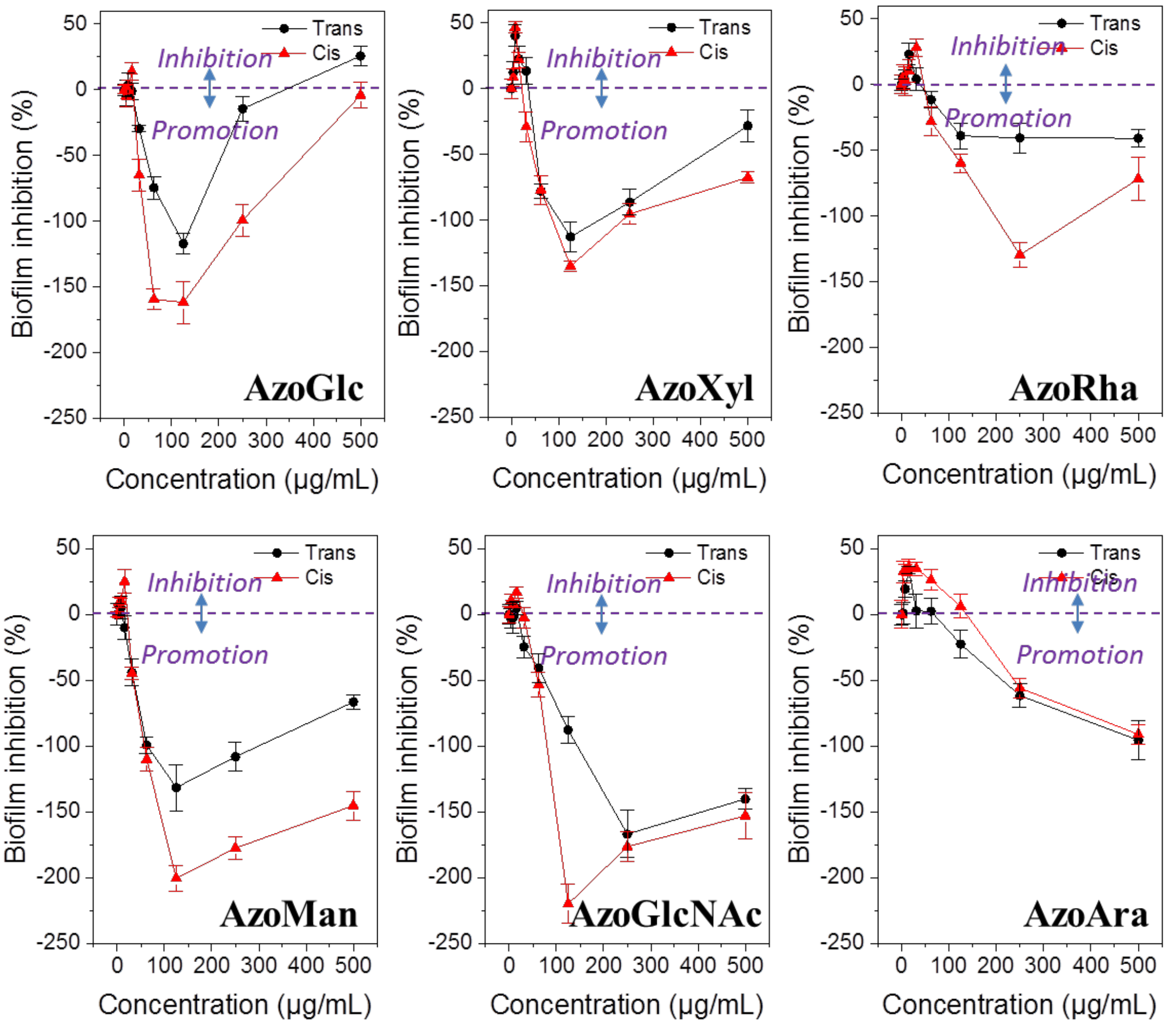

Figure S11. Concentration-dependent influence of *trans* and cis photoisomers of surfactants on biofilm forming ability of *P. aeruginosa* at 37 °C after 24 h of exposure.

Bacteria preparation for differential dynamic microscopy (DDM)

Overnight cultures of *P. aeruginosa* were grown in Luria-Bertani (LB) broth (10% tryptone, 5% yeast extract and 5% NaCl) using a shaking incubator at 37 °C and shaking speed of 120 rpm. A fresh culture was inoculated as 1:100 dilution of overnight grown cells in tryptone broth (TB) and grown for further 4 h to obtain actively growing late exponential phase bacteria. Bacterial suspensions were gently mixed with the surfactant solutions before use.

Differential dynamic microscopy (DDM)

To characterise the effect of different surfactants on the swimming motility of *P. aeruginosa*, we used differential dynamic microscopy (DDM).[7] Experiments were performed on an Olympus IX-71 inverted optical microscope equipped with a mikrotron MC1362 CMOS camera and Euresys Grablink full frame grabber. The bacteria were transferred into a rectangular capillary tube (Vitrotube W2540 8 mm width, 0.4 mm internal diameter height) and sealed with Vaseline. Samples were left for one minute after loading, and imaged with a 10X phase contrast objective under Kohler illumination. Videos were recorded with a frame size of 1024 x 1024 pixels at 100 frames per second for 60 seconds. A custom written LabVIEW (National Instruments) program was used to extract the differential intensity correlation functions $g(q,t)$ (DICF) from the videos using the following equation:

$$g(q,t) = A(q)[1 - f(q,\tau)] + B(q)$$

where $A(q)$ is related to the scattering properties of the sample and microscope, and $B(q)$ is a camera noise background.$f(q,\tau)$ is the intermediate scattering function, which is modelled using:

$$f(q,\tau) = (1-\alpha)e^{-Dq^2\tau} + \alpha e^{-Dq^2\tau}\left[\left(\frac{Z+1}{Zq\bar{v}\tau}\right)\frac{\sin(Z\tan^{-1}\Delta)}{(1+\Delta^2)^{\frac{Z}{2}}}\right]$$

Where $\Delta = (q\bar{v}\tau)/\,(Z+1)$ and $\sigma = \bar{v}(Z+1)^{-1/2}$

The motile parameters obtained are the fraction of motile swimmers α, mean velocity $\bar{v}$, velocity distribution σ, and diffusion coefficient of non-motile cells D.

Each video is transformed into 511 data sets of $g(q,t)$ vs. q and fitted with Eq. 1 using Igor pro (Wavemetrics) to yield six parameters: $A(q)$, $B(q)$, $\bar{v}$, D, α, σ. The parameters are averaged over the range where the fitted data is constant ($q{\sim}0.5\ to\ 2\ um^{-1}$).

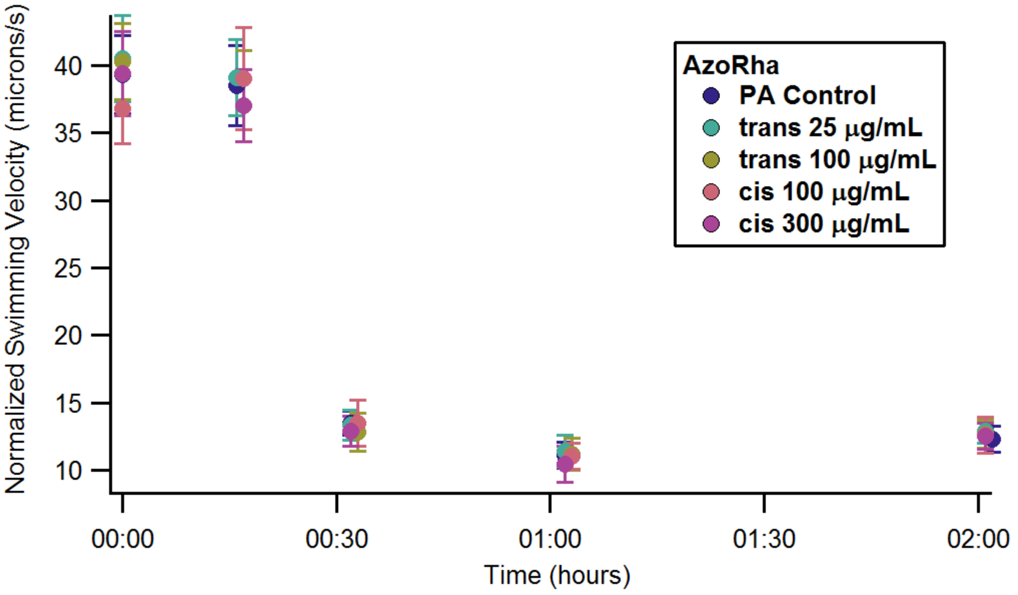

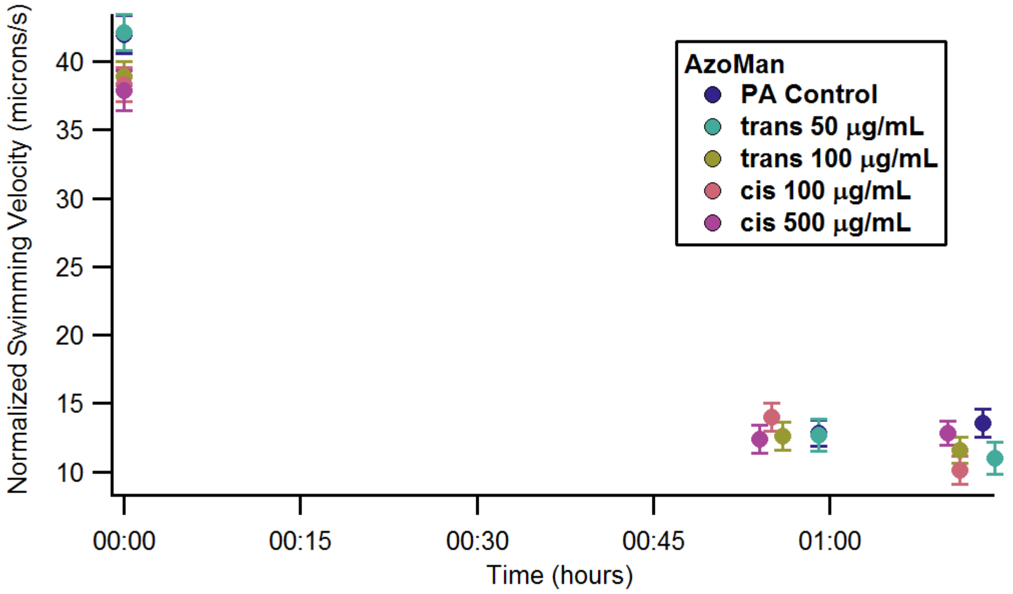

Figure S12. Influence of *cis*- and *trans*-dominated PSS of **AzoRha** and **AzoMan** on swimming motility of *P. aeruginosa* MDR283/1-6.

Swarming motility assays

Compounds were tested for their influence on swarming motility of Gram-negative *P. aeruginosa*. Swarm plate (0.8% nutrient broth, 0.5% glucose, 0.5% agar) was air dried for 5-10 min before use. The compounds were dissolved in MilliQ deionised water and then added to each agar medium at a final concentration of 1 to 100 µg/mL. An equal amount of deionised water without any compound was added to the agar medium to serve as a control. A fixed concentration of overnight grown bacterial culture (2 µL) was inoculated at the centre of the agar surface, and the plates were incubated at 30 °C for 22 h.

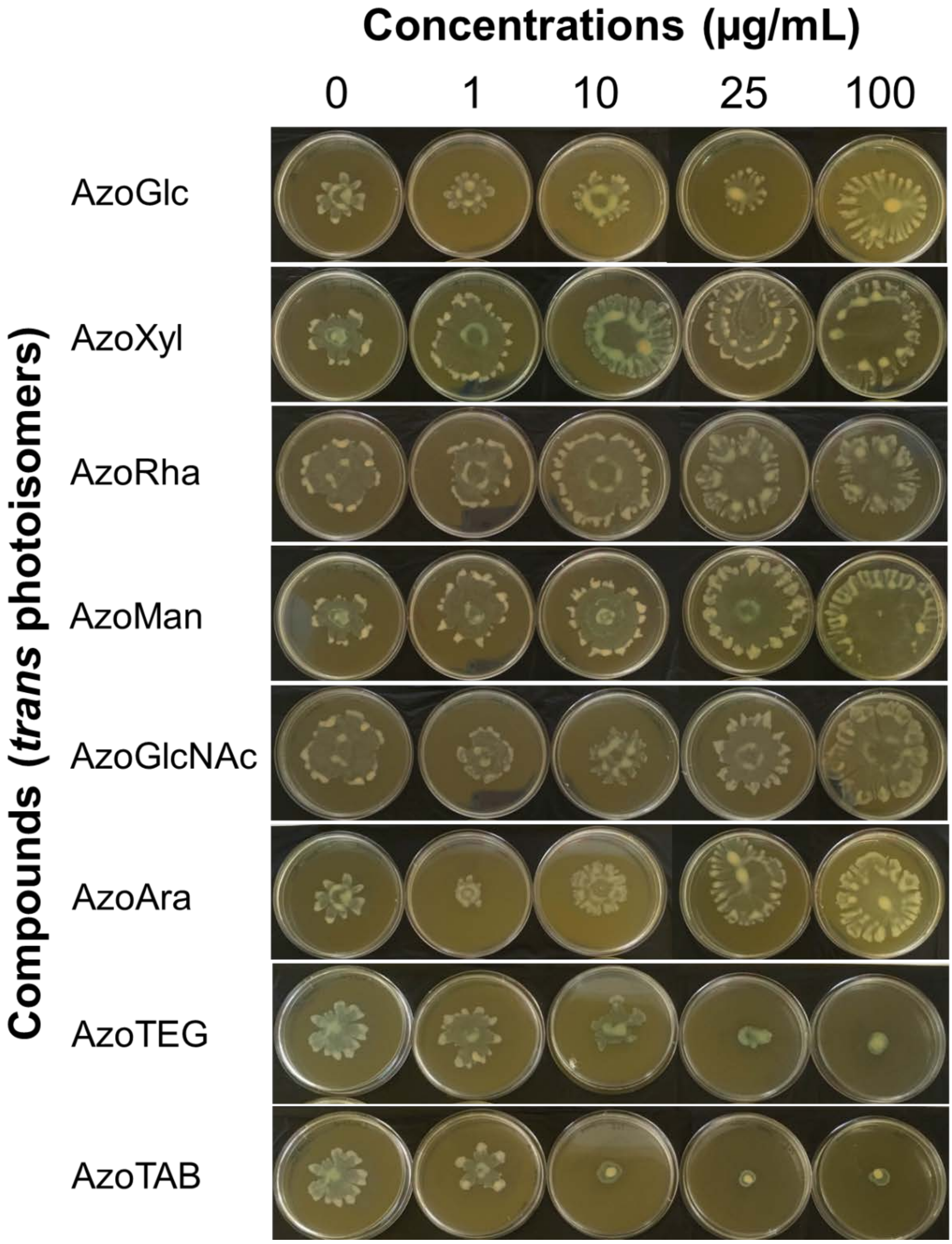

Figure S13. Influence of the increasing concentration of *trans* photo-isomer of photosurfactants and controls on swarming motility of *P. aeruginosa* MDR283/1-6, a swarming-positive strain.

2.0 Synthesis and analytical data

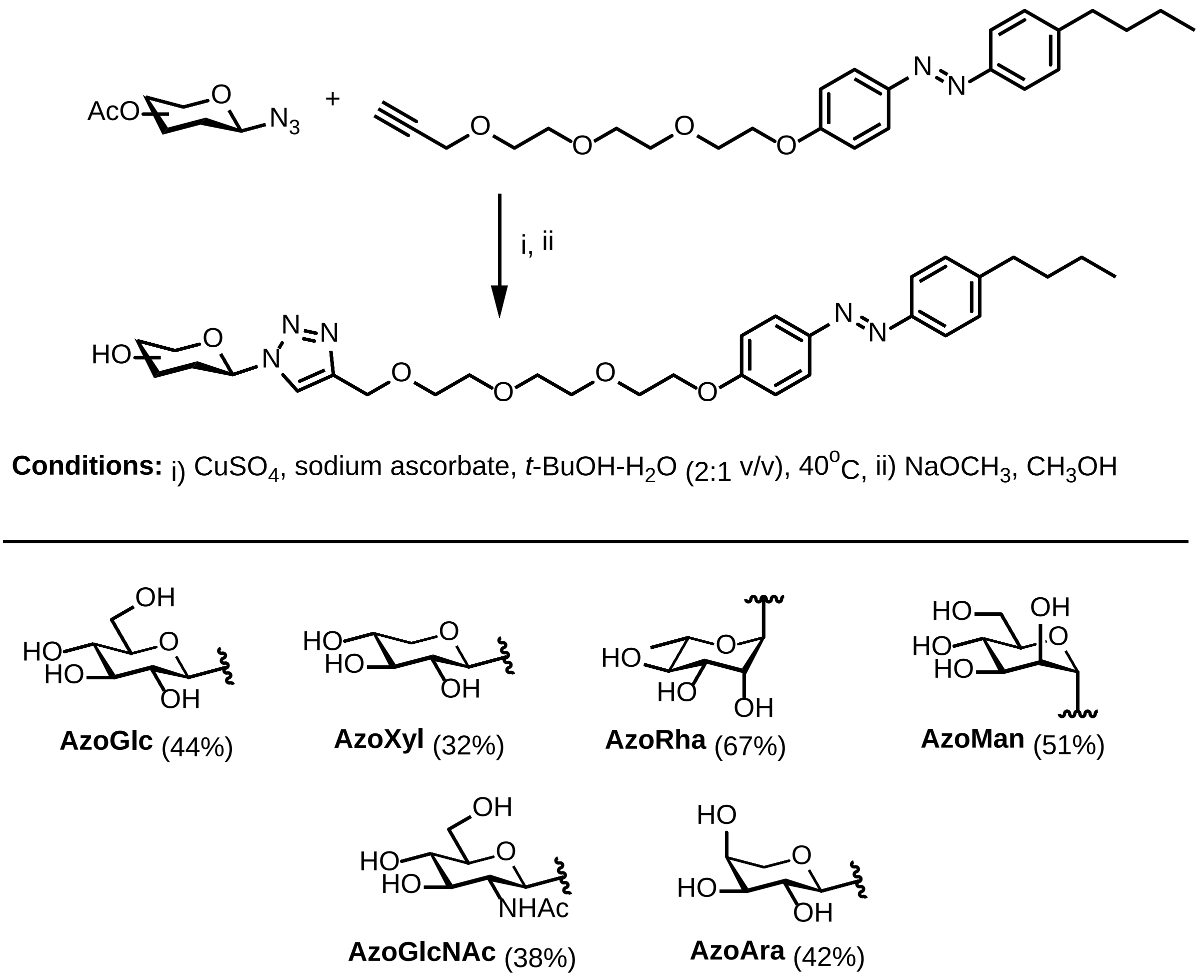

Scheme S1. Synthesis of carbohydrate-based photosurfactants. The synthesis of **AzoGlc** has been reported previously.[1]

Carbohydrate surfactants **1-6** were synthesized as previously described (Scheme S1).[1-3] A mixture of the corresponding D-glycopyranosyl azide (1.0 equiv)[6] and azobenzenene propargyl ether[1] (1.0 equiv.) was suspended in *tert*-BuOH/H_2O (2:1 v/v, 0.5 mM). Sodium ascorbate (0.4 equiv.) and $CuSO_4.5H_2O$ (0.2 equiv.) was added and the deep yellow suspension was stirred vigorously for 2h at 40 °C. TLC analysis (ethyl acetate/petroleum ether 40-60, 4:1 v/v) showed complete consumption of the azide and alkyne, with formation of a polar product (R_f ca. 0.3). The reaction mixture was extracted into ethyl acetate (10 mL), and washed with water (10 mL) and brine (10 mL). The organic layer was dried (Na_2SO_4), filtered and evaporated to dryness. The resulting yellow oil was re-dissolved in dry methanol. A 0.5M solution of sodium methoxide in methanol (0.1 equiv.) was then added and the reaction stirred at room temperature for one hour, at which time LC-MS analysis (20 to 100% B over 15 min) showed reaction

completion. The reaction was neutralized with Amberlite IR-120 acidic ion exchange resin, filtered and concentrated under reduced pressure. Surfactants **1-6** were subsequently purified by reversed-phase preparative HPLC and isolated as deep yellow solids following lyophilization (21-67% yields).

2-[2-[2-(4-n-butylazophenyl phenoxy)ethoxy)ethoxy]ethoxy]methyl β-D-xylopyranosyl-1,2,3-triazole (AzoXyl)

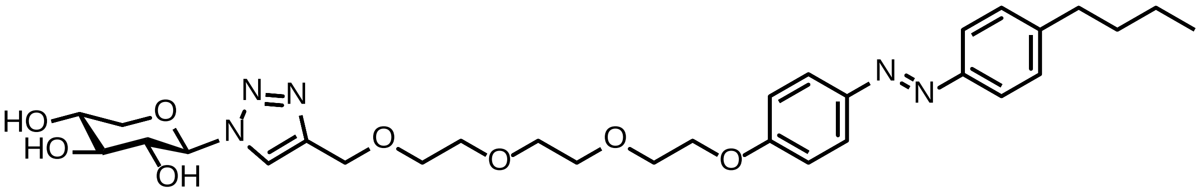

AzoXyl

Yield: 32% Mp = 156.5 °C; $[\alpha]_D^{20}$ 3.1 (c, 2.65 in CH_3OH); FT-IR: ν_{max}/cm^{-1} 3338, 2940, 2876, 1628, 1449, 1375, 1252, 1099, 1021, 823; ^{1}H NMR (600 MHz, CD_3OD) δ 8.13 (s, 1H, triazole H), 7.86 (d, *J* = 9.0 Hz, 2H, Ar*H*), 7.77 (d, *J* = 8.3 Hz, 2H, Ar*H*), 7.3 (d, *J* = 8.3 Hz, 2H, Ar*H*), 7.06 (d, *J* = 9.0 Hz, 2H, Ar*H*), 5.50 (d, *J* = 9.14 Hz, 1H, H-1), 4.65 (s, 2H, C*H*$_2$), 4.20-4.19 (m, 2H, H-3), 4.00-3.98 (m, 1H), 3.90-3.84 (m, 2H, *H*-2, *H*-4), 3.74-3.56 (m, 10H), 3.51-3.43 (m, 2H, H-5), 2.68 (t, *J* = 7.7 Hz, 2H, CH_2), 1.67-1.62 (m, 2H, CH_2), 1.42-1.36 (m, 2H, CH_2), 0.96 (t, *J* = 7.4 Hz, 3H, CH_3); ^{13}C NMR (125 MHz, d_6-DMSO) δ 161.5, 150.7, 146.7, 146.2, 144.4, 129.7, 124.9, 123.5, 122.8, 115.5, 88.6, 77.6, 72.5, 70.4, 70.3, 70.2, 69.6, 69.5, 68.8, 68.1, 63.9, 35.1, 33.4, 22.2, 14.2. LC-MS: m/z 599.8 [M + H]+, HRMS (ESI-ToF) calculated for $C_{30}H_{41}N_5NaO_8$ 622.2853 $[M + Na]^+$, found 622.2850 $[M + Na]^+$.

2-[2-[2-(4-n-butylazophenyl phenoxy)ethoxy)ethoxy]ethoxy]methyl α-L-rhamnopyranosyl-1,2,3-triazole (AzoRha)

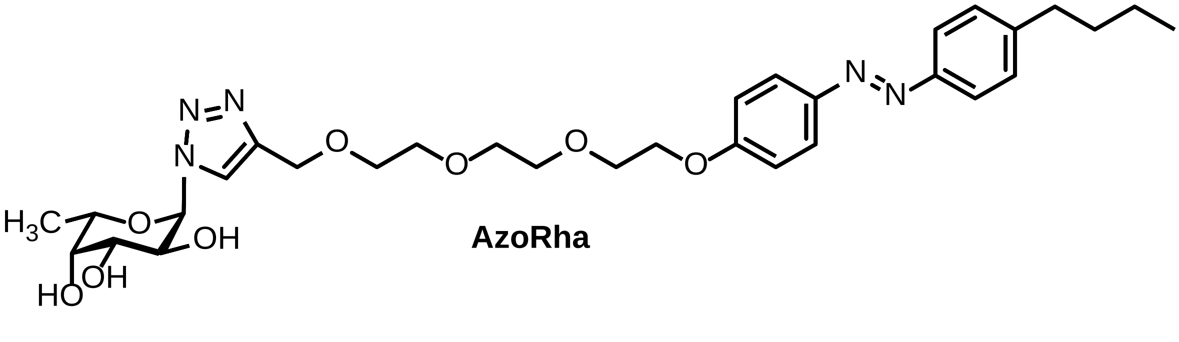

AzoRha

Yield 67% Mp = 64.9 ºC; $[\alpha]_D^{20}$ 11.0 (c, 3.68 in CH_3OH); FT-IR: ν_{max}/cm^{-1} 3357, 2928, 2873, 1598, 1453, 1248, 1080, 1024, 842; ^1H NMR (600 MHz, CD_3OD) δ 8.15 (s, 1H, triazole), 7.86 (d, *J* = 9.0 Hz, 2H, Ar*H*), 7.8 (d, *J* = 8.4 Hz, 2H, Ar*H*), 7.32 (d, *J* = 8.4 Hz, 2H, Ar*H*), 7.06 (d, *J* = 9.0 Hz, 2H, Ar*H*), 6.0 (d, *J* = 1.1 Hz, 1H, H-1), 4.6 (s, 2H, CH_2), 4.21-4.19 (m, 2H, H-4, H-5), 3.87-3.86 (m, 2H, H-2, H-3), 3.71-3.63 (m, 10H, CH_2), 3.55-3.47 (m, 2H, CH_2), 2.69 (t, *J* = 7.6 Hz, 2H, CH_2), 1.67-1.62 (m, 2H, CH_2), 1.41-1.36 (m, 5H, H-6 CH_3, CH_2), 1.0 (t, *J* = 7.4 Hz, 3H, CH_3); ^{13}C NMR (125 MHz, d_6-DMSO) δ 161.5, 150.8, 146.7, 146.2, 143.9, 129.7, 124.9, 123.9, 122.8, 115.5, 86.3, 75.4, 73.4, 71.7, 71.1, 70.4, 70.3, 70.2, 69.5, 69.3, 68.1, 63.9, 35.1, 33.4, 22.2, 18.3, 14.2. LC-MS: m/z 613.8 [M + H]+, HRMS (ESI-ToF) calculated for $C_{31}H_{43}N_5NaO_8$ 636.3009 $[M + Na]^+$, found 636.3004 $[M + Na]^+$

2-[2-[2-(4-n-butylazophenyl phenoxy)ethoxy)ethoxy]ethoxy]methyl α-D-mannopyranosyl-1,2,3-triazole (AzoMan)

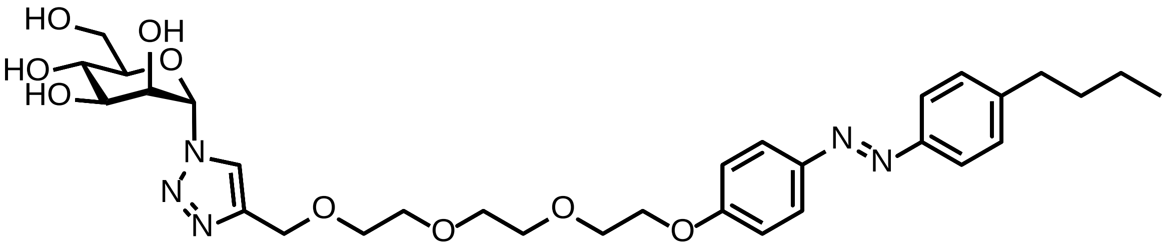

AzoMan

Yield: 51% Mp = 164.6 ºC; $[\alpha]_D^{20}$ 70.3 (c, 0.72 in CH3OH); FT-IR: ν_{max}/cm^{-1} 3297, 2928, 2873, 1598, 1442, 1248, 1088, 1032, 838; ^1H NMR (600 MHz, CD_3OD) δ 8.14 (s, 1H, triazole), 7.86 (d, *J* = 9.1 Hz, 2H, Ar*H*), 7.77 (d, *J* = 8.3 Hz, 2H, Ar*H*), 7.32 (d, *J* = 8.4 Hz, 2H, Ar*H*), 7.07 (d, *J* = 9.1 Hz, 2H, Ar*H*), 6.0 (d, *J* = 2.6 Hz, 1H, H-1), 4.69-4.64 (m, 3H, CH_2, H-2), 4.21-4.19 (m, 2H, H-6), 4.07-4.05 (m, 1H, H-5), 3.87-3.86 (m, 2H, H-3, H-4), 3.82-3.65 (m, 12H, CH_2), 2.68 (t, *J* = 7.7Hz, 2H, CH_2), 1.66-1.63 (m, 2H, CH_2), 1.41-1.37 (m, 2H, CH_2), 1.0 (t, *J* = 7.4 Hz, 3H, CH_3); ^{13}C NMR (125 MHz, d_6-DMSO) δ 161.5, 150.8, 146.7, 146.2, 144.4, 129.7, 124.9, 124.1, 122.8, 115.5, 86.3, 78.7, 71.7, 70.4, 70.3, 70.2, 69.6, 69.3, 68.7, 68.1, 68.0, 63.9, 61.3, 35.1, 33.4, 22.2, 14.2; LC-MS: *m/z* 629.8 [M + H]+, HRMS (ESI-ToF) calculated for $C_{31}H_{43}N_5NaO_9$ 652.2959 $[M + Na]^+$, found 652.2951 $[M + Na]^+$

2-[2-[2-(4-n-butylazophenyl phenoxy)ethoxy)ethoxy]ethoxy]methyl 2-acetamido-2-deoxy-β-D-glucopyranosyl-1,2,3-triazole (AzoGlcNAc)

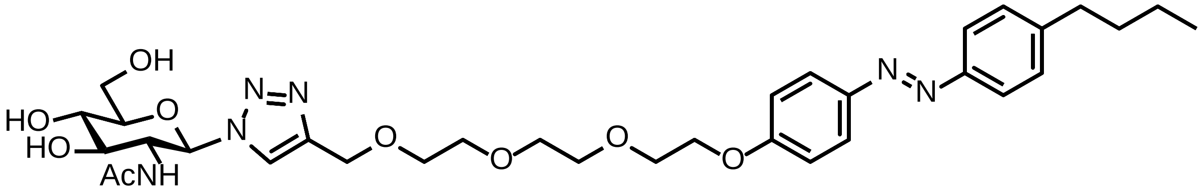

AzoGlcNAc

Yield 38% Mp = 173.2 °C; $[\alpha]_D^{20}$ -6.1 (c, 2.68 in CH_3OH); FT-IR: ν_{max}/cm^{-1} 3350, 2947, 2824, 1628, 1606, 1457, 1378, 1099, 1021, 823; ^1H NMR (600 MHz, CD_3OD) δ 8.1 (s, 1H, triazole), 7.9 (d, *J* = 9.0 Hz, 2H, Ar*H*), 7.8 (d, *J* = 8.4 Hz, 2H, Ar*H*), 7.33 (d, *J* = 8.4 Hz, 2H, Ar*H*), 7.1 (d, *J* = 9.0 Hz, 2H, Ar*H*), 5.79 (d, *J* = 9.7 Hz, 1H, H-1), 4.63-4.60 (m, 2H), 4.23-4.19 (m, 3H, CH_2, H-2), 3.90-3.87 (m, 2H), 3.76-3.53 (m, 12H, CH_2, H-3, H-4, H-5, H-6), 2.69 (t, *J* = 7.8 Hz, 2H, CH_2), 1.8 (s, 3H, NHAc CH_3), 1.68-1.63 (m, 2H, CH_2), 1.43-1.37 (m, 2H, CH_2), 1.0 (t, *J* = 7.4 Hz, 3H, CH_3); ^{13}C NMR (125 MHz, d_6-DMSO) δ 169.6, 161.5, 150.8, 146.7, 146.2, 144.2, 129.7, 124.9, 123.0, 122.8, 115.5, 86.5, 80.6, 74.4, 70.4, 70.3, 70.2, 69.3, 68.1, 63.8, 61.2, 55.0, 35.1, 33.4, 23.2, 22.2, 14.2. LC-MS: *m/z* 692.7 [M + Na]+, HRMS (ESI-ToF) calculated for $C_{33}H_{46}N_6NaO_9$ 693.3224 $[M + Na]^+$, found 693.3218 $[M + Na]^+$.

2-[2-[2-(4-n-butylazophenyl phenoxy)ethoxy)ethoxy]ethoxy]methyl α -L-arabinopyranosyl-1,2,3-triazole (AzoAra)

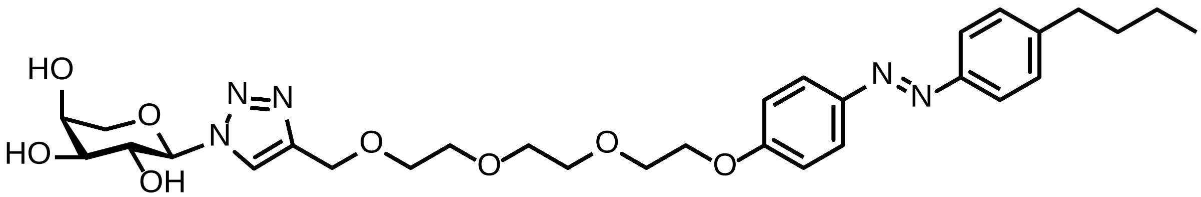

AzoAra

Yield: 42% Mp = 86.2 °C; $[\alpha]_D^{20}$ –15.8 (c, 3.36 in CH_3OH); FT-IR: ν_{max}/cm^{-1} ; 3372, 2932, 1598, 1449, 1378, 1252, 1088, 1024, 879, 823; ^1H NMR (400 MHz, d_6-DMSO) δ 8.2 (s, 1H, triazole), 7.9 (d, *J* = 9.0 Hz, 2H, Ar*H*), 7.8 (d, *J* = 8.2 Hz, 2H, Ar*H*), 7.4 (d, *J* = 8.4 Hz, 2H, Ar*H*), 7.1 (d, *J* = 9.0 Hz, 2H, Ar*H*), 5.4 (d, *J* = 9.2 Hz, 1H, H-1), 5.3 (d, *J* = 5.9 Hz, 1H, O*H*), 5.04 (d, *J* = 4.2 Hz, 1H, O*H*), 4.81 (d, *J* = 4.1 Hz, 1H, O*H*), 4.6 (s, 2H, CH_2), 4.21-4.19 (m, 2H, H-5), 4.09-4.03 (m, 1H, H-2), 3.85-3.75 (m, 5H, CH_2, H-4), 3.62-3.54 (m, 9H, CH_2 H-3), 2.65 (t, *J* = 7.6 Hz, 2H, CH_2), 1.62-1.55 (m, 2H, CH_2), 1.37-1.28 (m, 2H, CH_2), 0.9 (t, *J* = 7.3 Hz,

3H, CH_3); ^{13}C NMR (100MHz, d_6-DMSO) δ 161.5, 150.7, 146.7, 146.2, 144.4, 129.7, 124.9, 123.2, 122.8, 115.5, 88.8, 73.7, 70.4, 70.2, 69.8, 69.5, 69.3, 68.9, 68.1, 63.9, 35.1, 33.4, 22.2, 14.2. LC-MS: m/z 599.8 [M + H]+, HRMS (ESI-ToF) calculated for $C_{30}H_{41}N_5NaO_8$ 622.2852 $[M + Na]^+$, found 622.2916 $[M + Na]^+$.

3.0 References

1) R. F. Tabor, D. D. Tan, S. S. Han. S. A. Young, Z. L. E. Seeger, M. J. Pottage, C. J. Garvey, B. L. Wilkinson, *Chem. Eur. J.* 2014, **20**,13881-13884.
2) R. F. Tabor, M. J. Pottage, C. J. Garvey, B. L. Wilkinson, *Chem. Commun.* 2015, **51**, 5509-5512.
3) Y. Hu, J. B. Marlow, R. Ramanathan, W. Zou, H. G. Tiew, M. J. Pottage, V. Bansal, R. F. Tabor, B. L. Wilkinson, *Aust. J. Chem.* 2015, **68**, 1880-1884.
4) J. H. Jorgensen, G. V. Doern, L. A. Maher, A. W. Howell, J. S. Redding, *Antimicrob. Agents Chemother.* 1990, **34**, 2075-2080.
5) G. A. O'Toole, *J. Vis. Exp.* 2011, **47**, 2347.
6) T. Li, L. Guo, Y. Zhang, J. Wang, Z. Li, L. Lin, Z. Zhang, L. Li, J. Lin. W. Zhao, J. Li, P. G. Wang, *Carbohydr. Res.* 2011, **346**, 1083-1092.
7) a) L. G. Wilson, V. A. Martinez, J. Schwarz-Linek, J. Tailleur, G. Bryant, P. N. Pusey, and W. C. K. Poon, *Phys. Rev. Lett.* 2011, **106**, 18101-18104; b) V. A. Martinez, R. Besseling, O. A. Croze, J. Tailleur, M. Reufer, J. Scwarz-Linek, L. G. Wilson, M. A. Bees, and W. C. K. Poon, *Biophys. J.* 2012, **103**, 1637-1647.

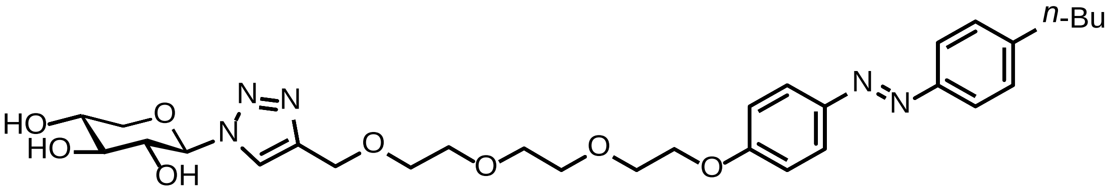
HO
HO
OH
O
N
N
N
O
O
O
O
N
N
n-Bu

AzoXyl

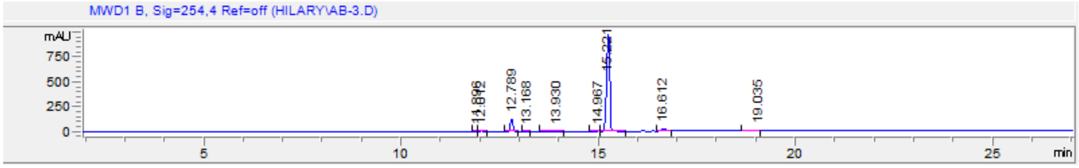
MWD1 B, Sig=254,4 Ref=off (HILARY\AB-3.D)
mAU
12.789
13.168
13.930
14.967
16.612
19.035
min

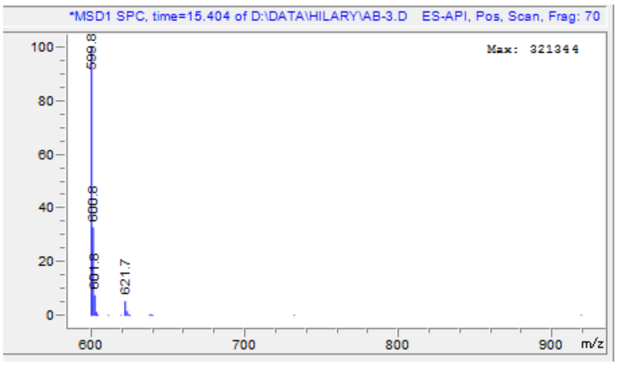
*MSD1 SPC, time=15.404 of D:\DATA\HILARY\AB-3.D ES-API, Pos, Scan, Frag: 70
Max: 321344
599.8
600.8
601.8
621.7
m/z

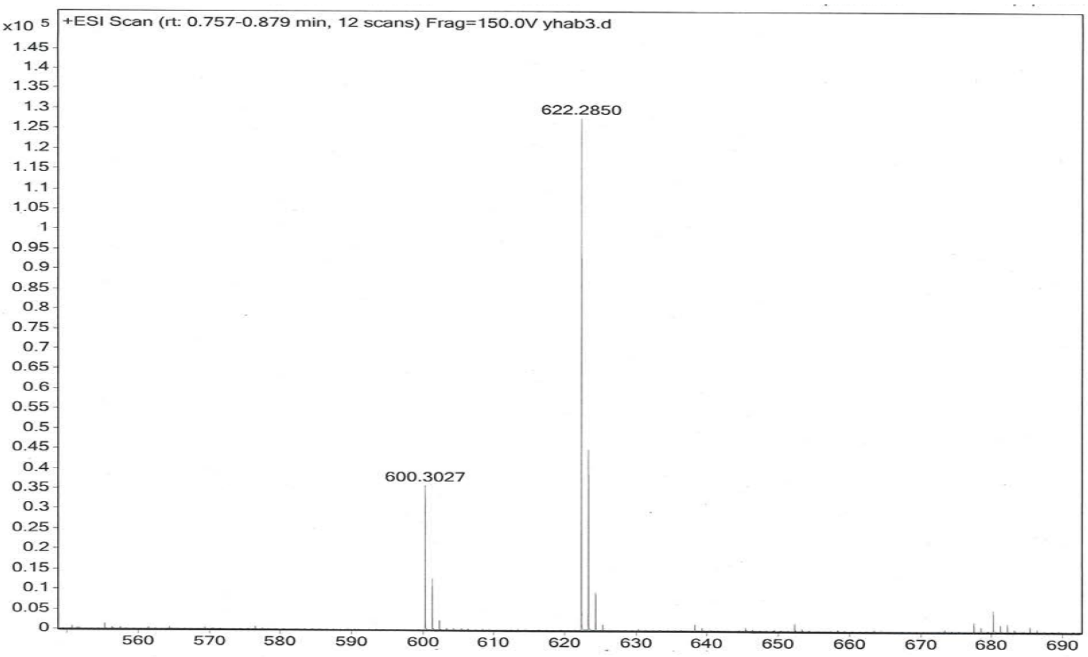
x10 5
+ESI Scan (rt: 0.757-0.879 min, 12 scans) Frag=150.0V yhab3.d
622.2850
600.3027

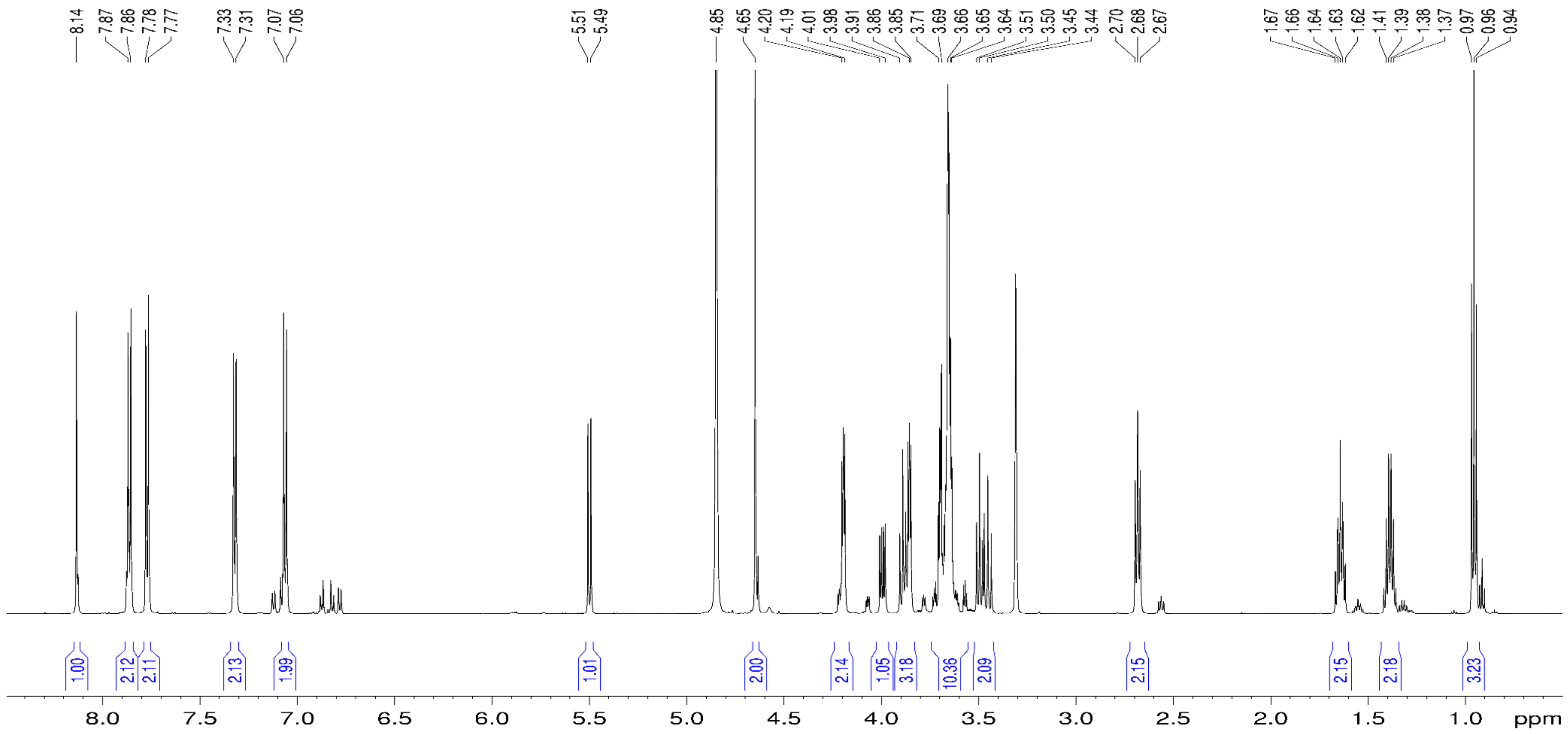
8.14
7.87
7.86
7.78
7.77
7.33
7.31
7.07
7.06
5.51
5.49
4.85
4.65
4.20
4.19
4.01
3.98
3.91
3.86
3.85
3.71
3.69
3.66
3.65
3.64
3.51
3.50
3.45
3.44
2.70
2.68
2.67
1.67
1.66
1.64
1.63
1.62
1.41
1.39
1.38
1.37
0.97
0.96
0.94
1.00
2.12
2.11
2.13
1.99
1.01
2.00
2.14
1.05
3.18
10.36
2.09
2.15
2.15
2.18
3.23
8.0
7.5
7.0
6.5
6.0
5.5
5.0
4.5
4.0
3.5
3.0
2.5
2.0
1.5
1.0
ppm

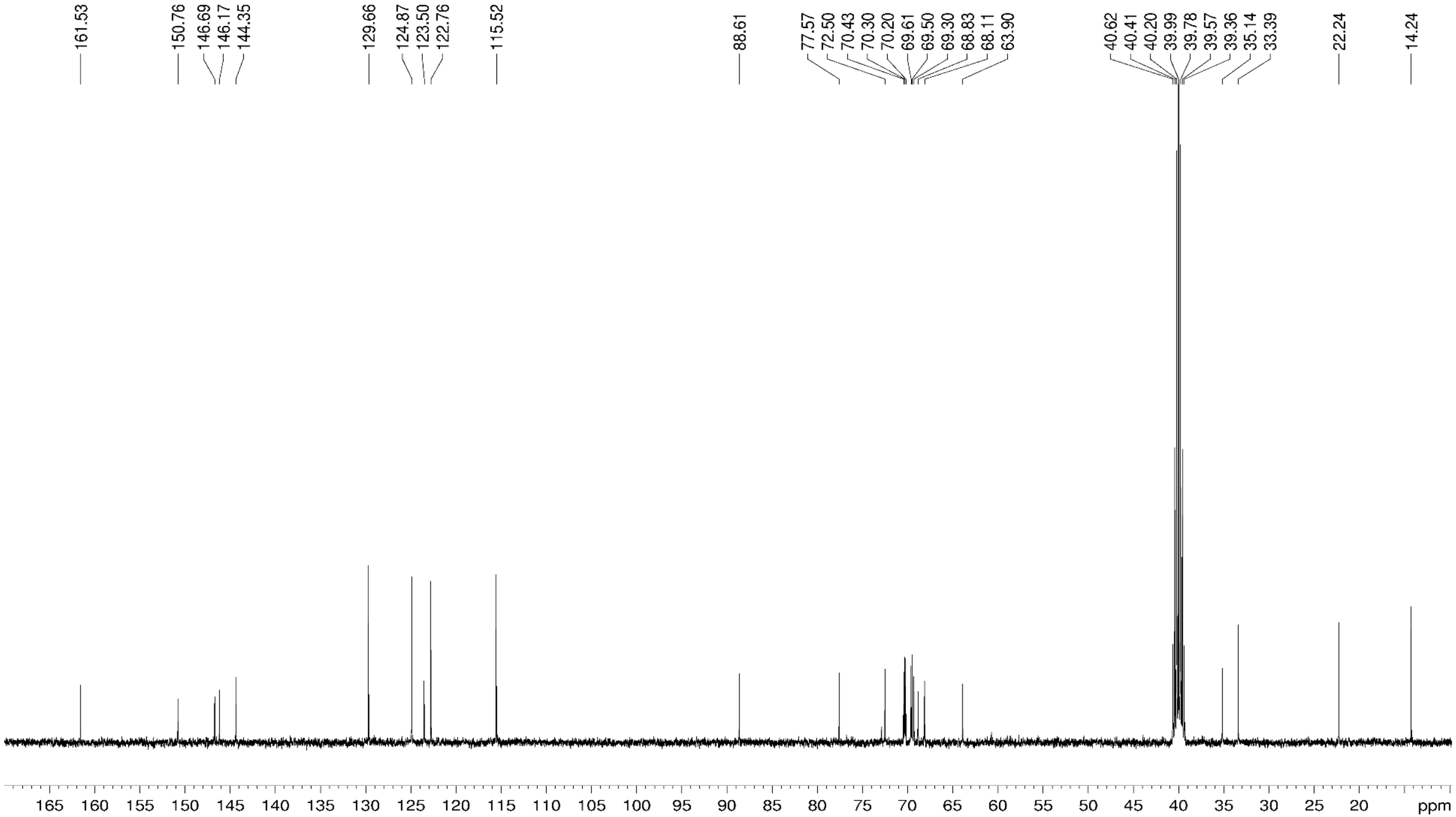

161.53
150.76
146.69
146.17
144.35
129.66
124.87
123.50
122.76
115.52
88.61
77.57
72.50
70.43
70.30
70.20
69.61
69.50
69.30
68.83
68.11
63.90
40.62
40.41
40.20
39.99
39.78
39.57
39.36
35.14
33.39
22.24
14.24
165 160 155 150 145 140 135 130 125 120 115 110 105 100 95 90 85 80 75 70 65 60 55 50 45 40 35 30 25 20
ppm

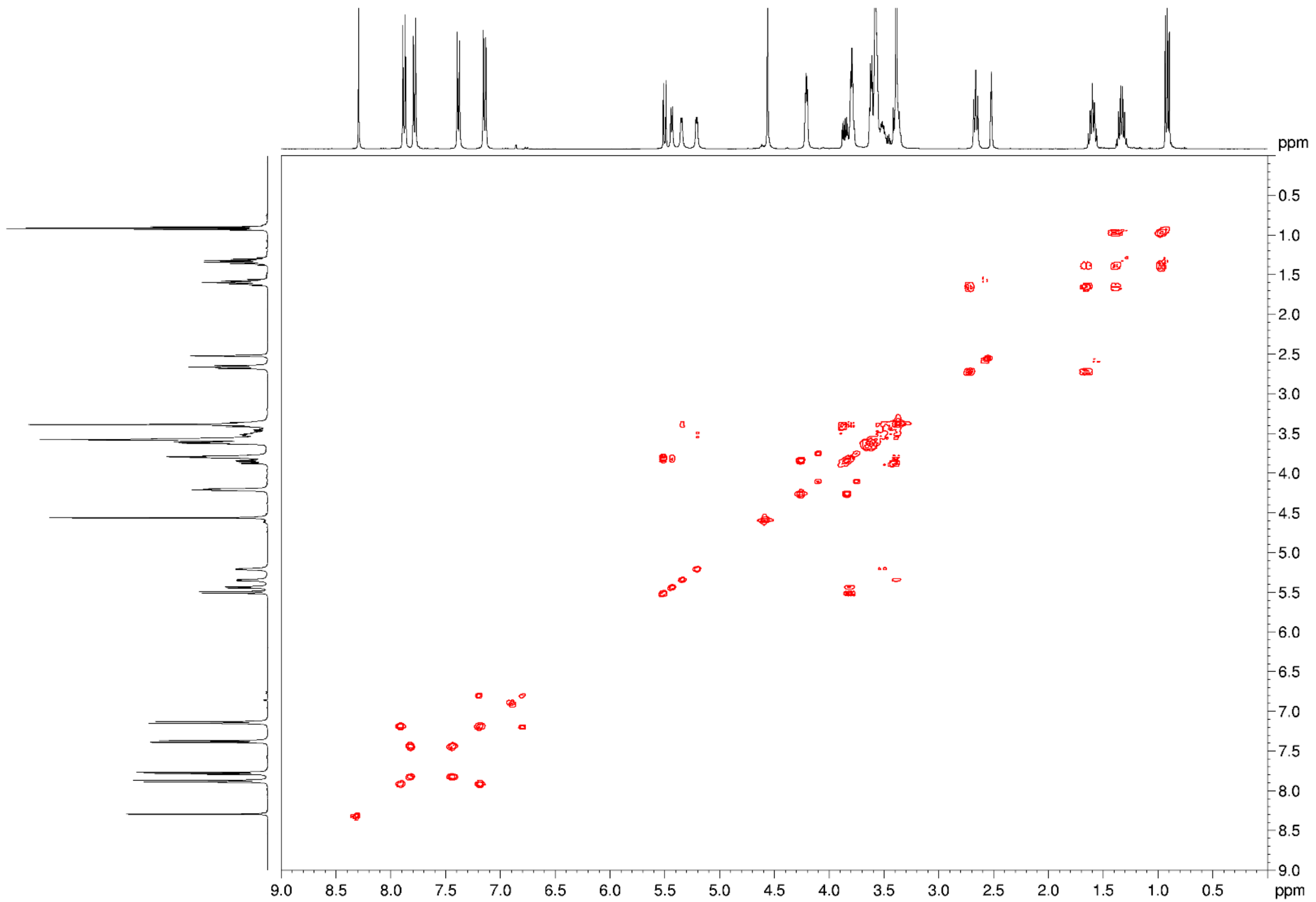
ppm
0.5
1.0
1.5
2.0
2.5
3.0
3.5
4.0
4.5
5.0
5.5
6.0
6.5
7.0
7.5
8.0
8.5
9.0
9.0
8.5
8.0
7.5
7.0
6.5
6.0
5.5
5.0
4.5
4.0
3.5
3.0
2.5
2.0
1.5
1.0
0.5
ppm

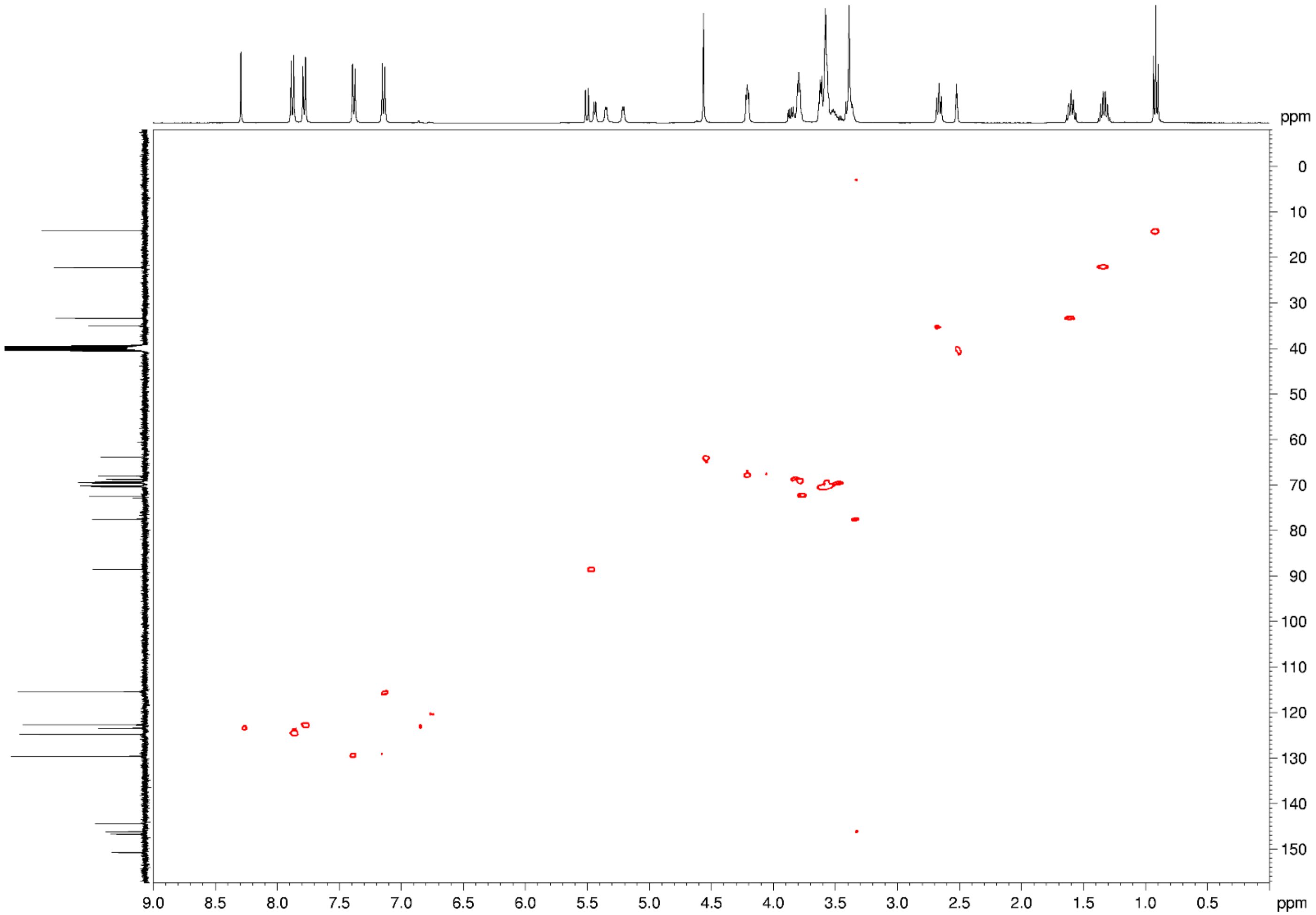

ppm
0
10
20
30
40
50
60
70
80
90
100
110
120
130
140
150
9.0
8.5
8.0
7.5
7.0
6.5
6.0
5.5
5.0
4.5
4.0
3.5
3.0
2.5
2.0
1.5
1.0
0.5
ppm

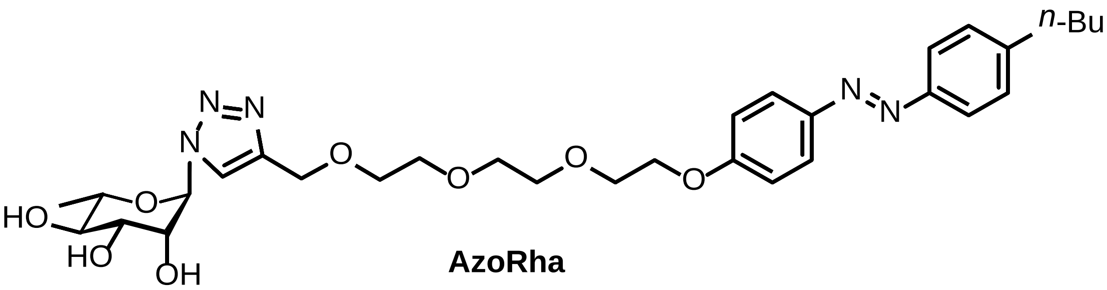

N
N
N
O
O
O
O
HO
HO
OH
N
N
n-Bu

AzoRha

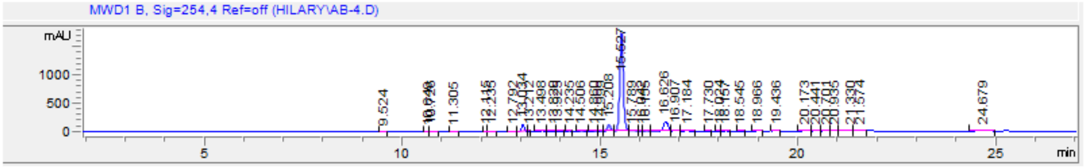

MWD1 B, Sig=254,4 Ref=off (HILARY\AB-4.D)
mAU
1000
500
0
5
10
15
20
25
min
9.524
11.305
15.627
16.626
24.679

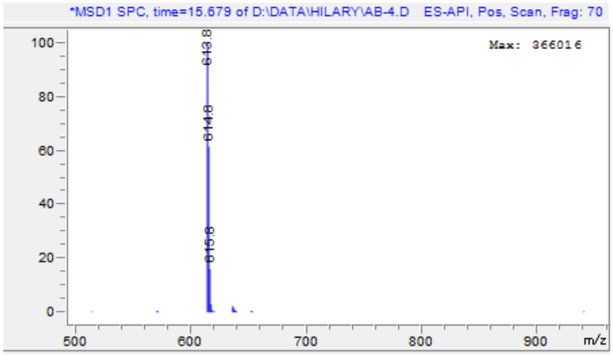

*MSD1 SPC, time=15.679 of D:\DATA\HILARY\AB-4.D ES-API, Pos, Scan, Frag: 70
Max: 366016
613.8
614.8
615.8
100
80
60
40
20
0
500
600
700
800
900
m/z

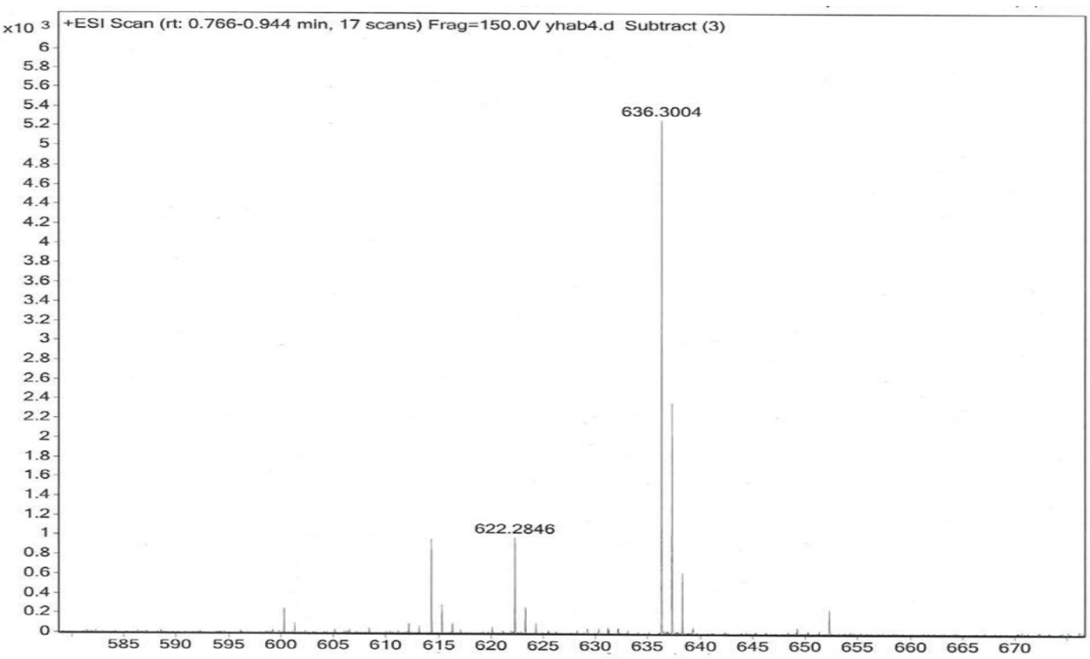

x10 3
+ESI Scan (rt: 0.766-0.944 min, 17 scans) Frag=150.0V yhab4.d Subtract (3)
636.3004
622.2846
585 590 595 600 605 610 615 620 625 630 635 640 645 650 655 660 665 670

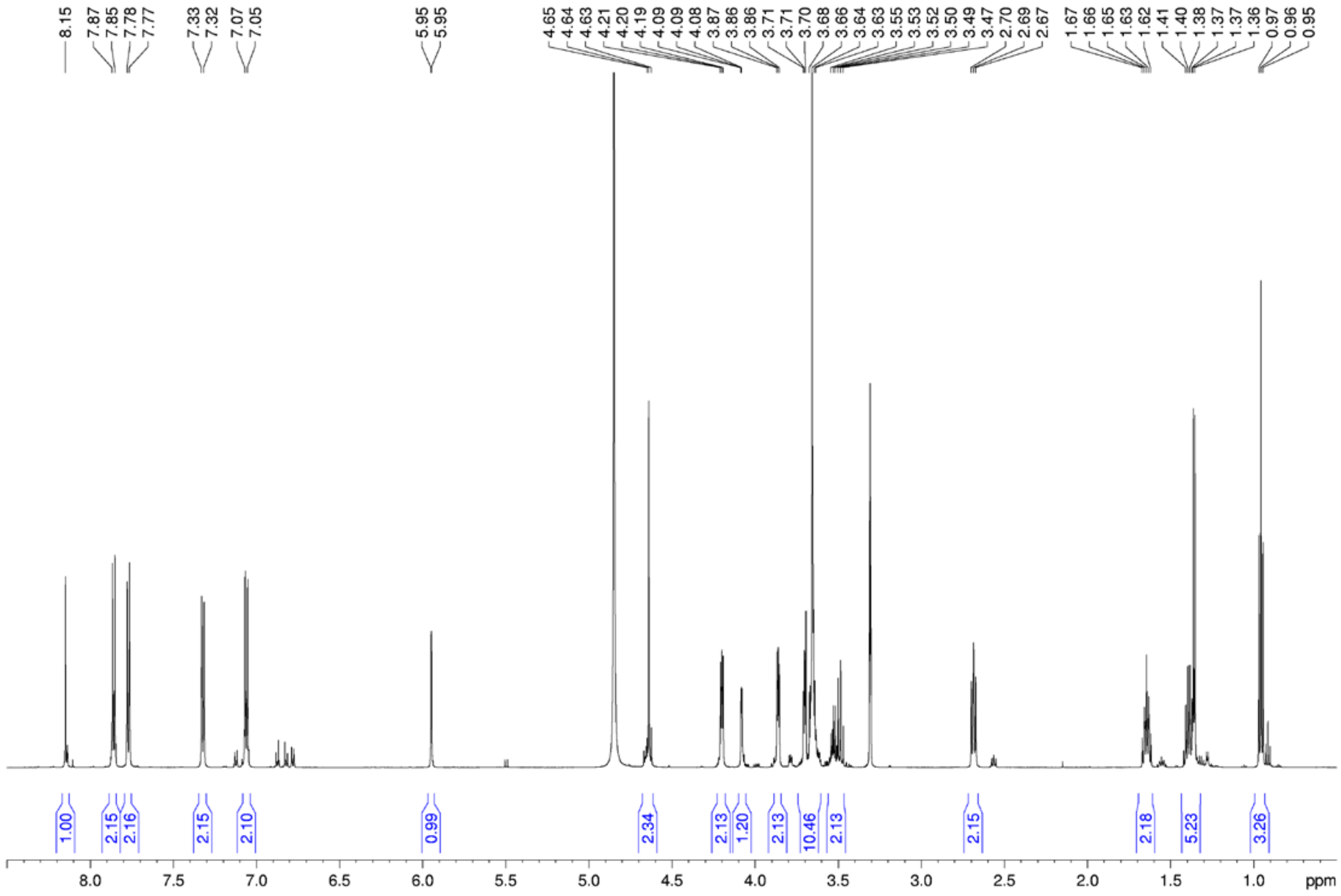
8.15
7.87
7.85
7.78
7.77
7.33
7.32
7.07
7.05
5.95
5.95
4.65
4.64
4.63
4.21
4.20
4.19
4.09
4.09
4.08
3.87
3.86
3.86
3.71
3.71
3.70
3.68
3.66
3.64
3.63
3.55
3.53
3.52
3.50
3.49
3.47
2.70
2.69
2.67
1.67
1.66
1.65
1.63
1.62
1.41
1.40
1.38
1.37
1.37
1.36
0.97
0.96
0.95
1.00
2.15
2.16
2.15
2.10
0.99
2.34
2.13
1.20
2.13
10.46
2.13
2.15
2.18
5.23
3.26
8.0
7.5
7.0
6.5
6.0
5.5
5.0
4.5
4.0
3.5
3.0
2.5
2.0
1.5
1.0
ppm

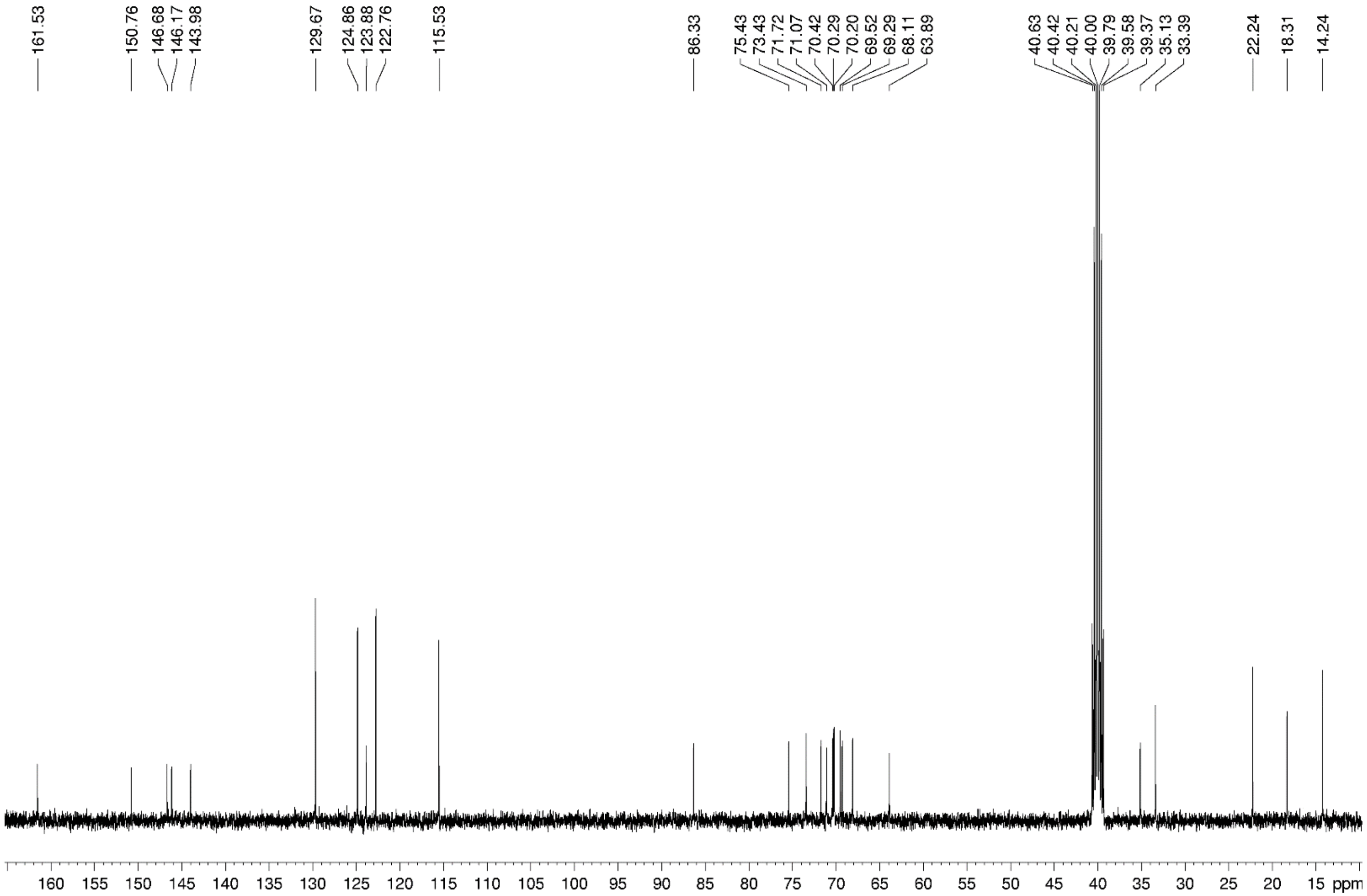
161.53
150.76
146.68
146.17
143.98
129.67
124.86
123.88
122.76
115.53
86.33
75.43
73.43
71.72
71.07
70.42
70.29
70.20
69.52
69.29
68.11
63.89
40.63
40.42
40.21
40.00
39.79
39.58
39.37
35.13
33.39
22.24
18.31
14.24
160 155 150 145 140 135 130 125 120 115 110 105 100 95 90 85 80 75 70 65 60 55 50 45 40 35 30 25 20 15 ppm

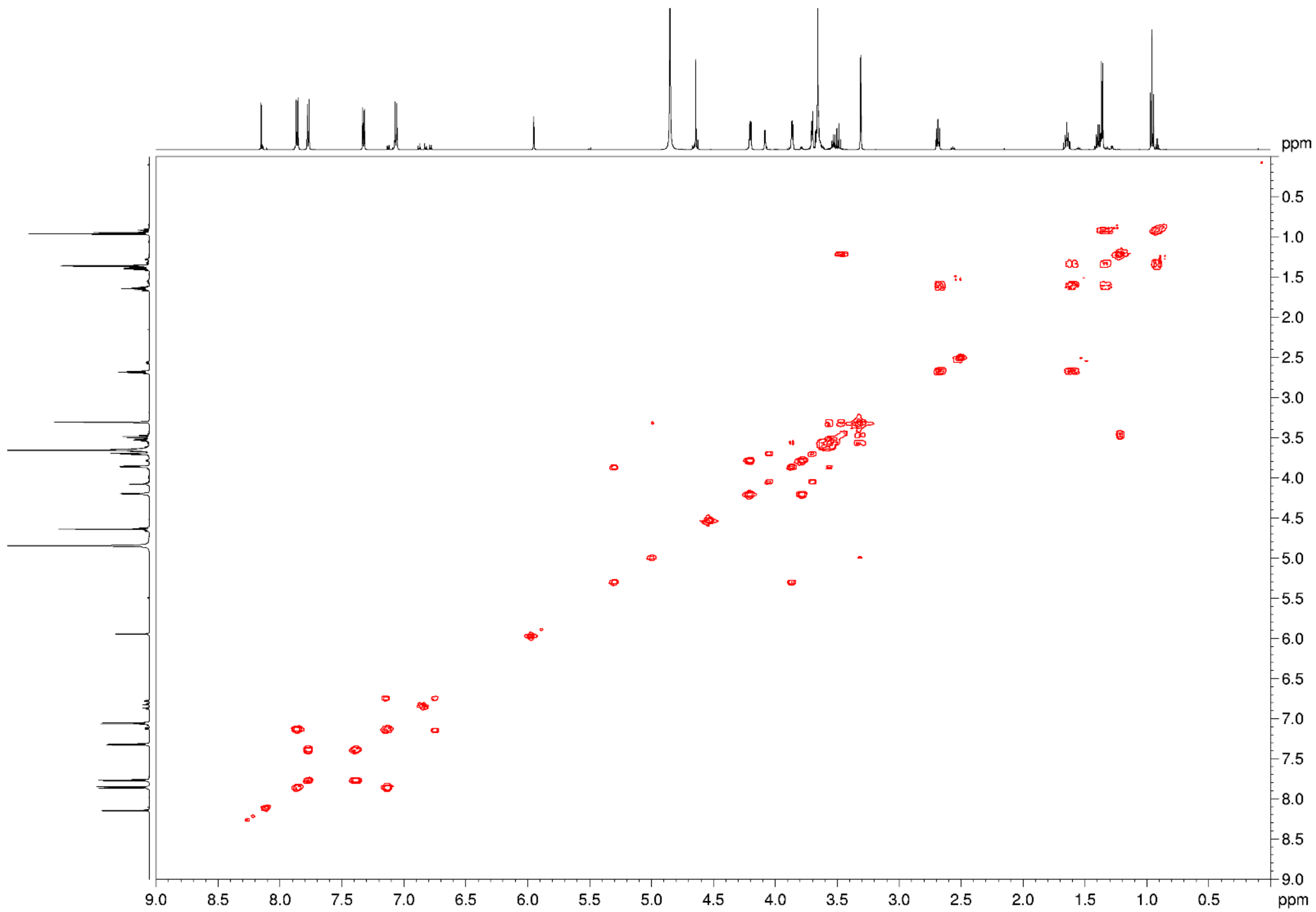
ppm
0.5
1.0
1.5
2.0
2.5
3.0
3.5
4.0
4.5
5.0
5.5
6.0
6.5
7.0
7.5
8.0
8.5
9.0
9.0
8.5
8.0
7.5
7.0
6.5
6.0
5.5
5.0
4.5
4.0
3.5
3.0
2.5
2.0
1.5
1.0
0.5
ppm

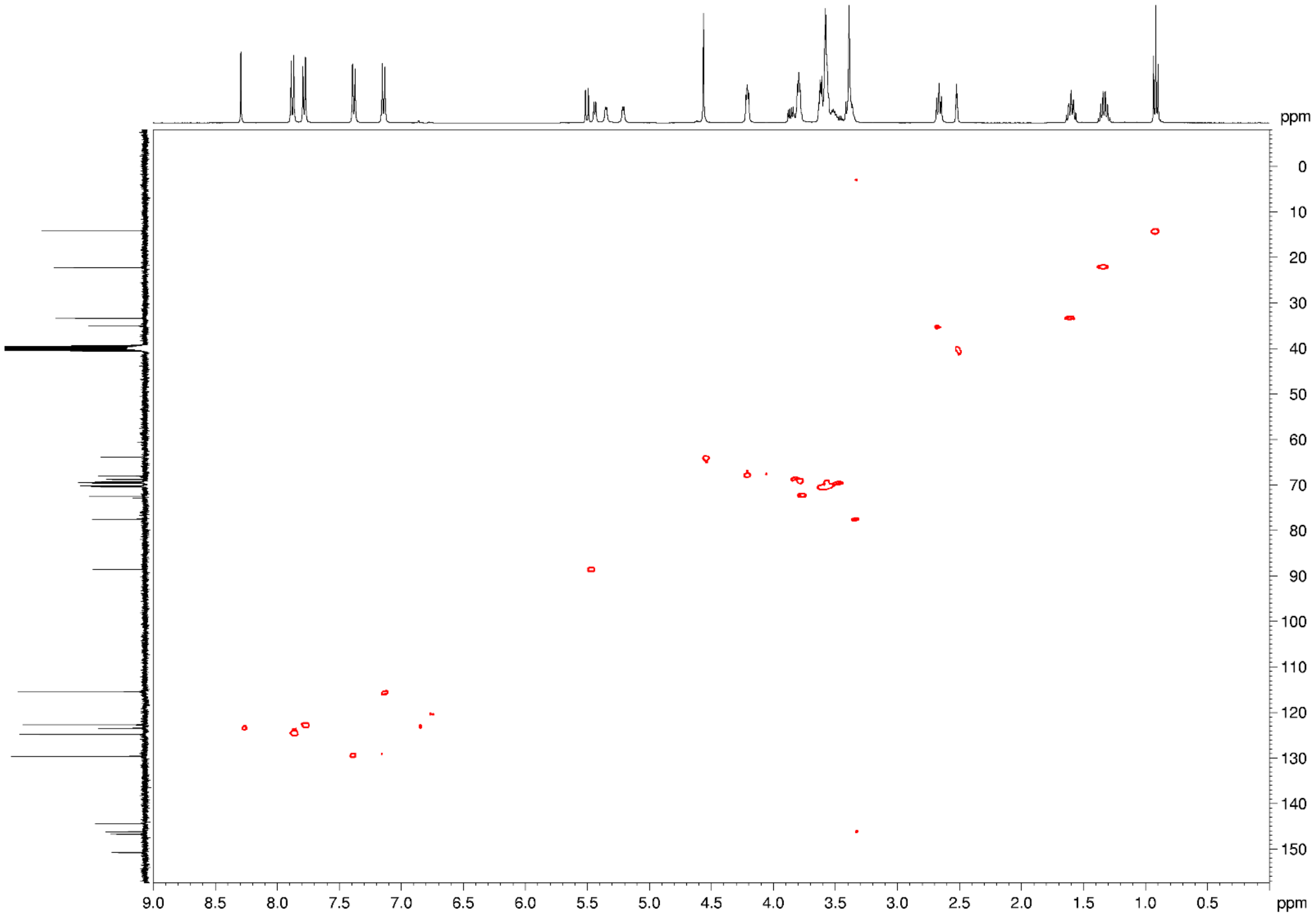
ppm
0
10
20
30
40
50
60
70
80
90
100
110
120
130
140
150
9.0 8.5 8.0 7.5 7.0 6.5 6.0 5.5 5.0 4.5 4.0 3.5 3.0 2.5 2.0 1.5 1.0 0.5
ppm

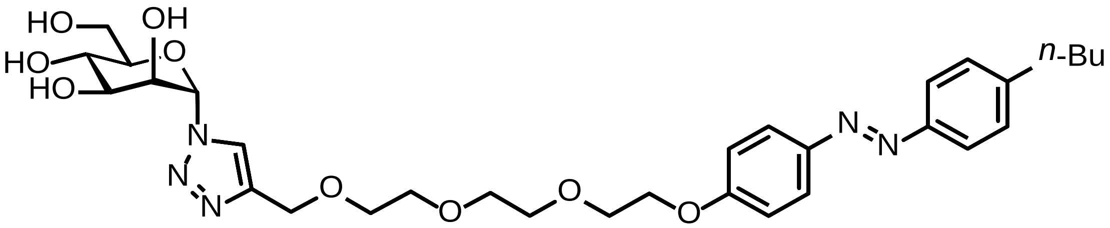
HO
OH
O
HO
HO
N
N
N
O
O
O
O
N
N
n-Bu

AzoMan

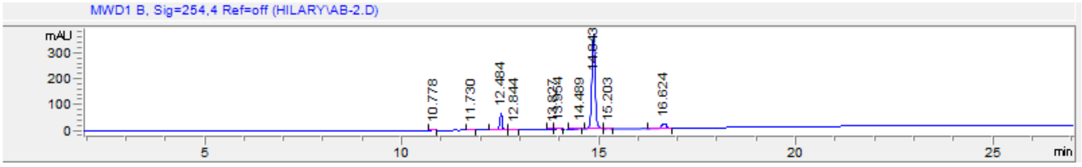
MWD1 B, Sig=254,4 Ref=off (HILARY\AB-2.D)
mAU
300
200
100
0
10.778
11.730
12.484
12.844
13.827
13.954
14.489
14.843
15.203
16.624
5
10
15
20
25
min

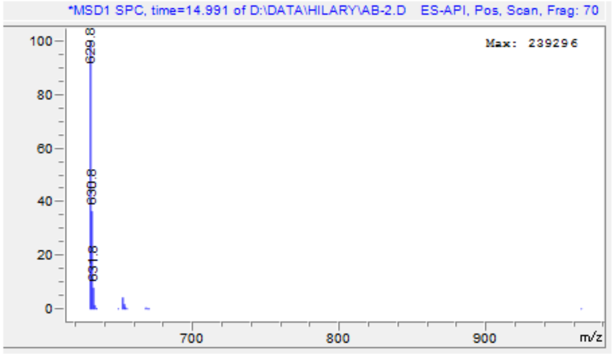
*MSD1 SPC, time=14.991 of D:\DATA\HILARY\AB-2.D ES-API, Pos, Scan, Frag: 70
Max: 239296
100
80
60
40
20
0
629.8
630.8
631.8
700
800
900
m/z

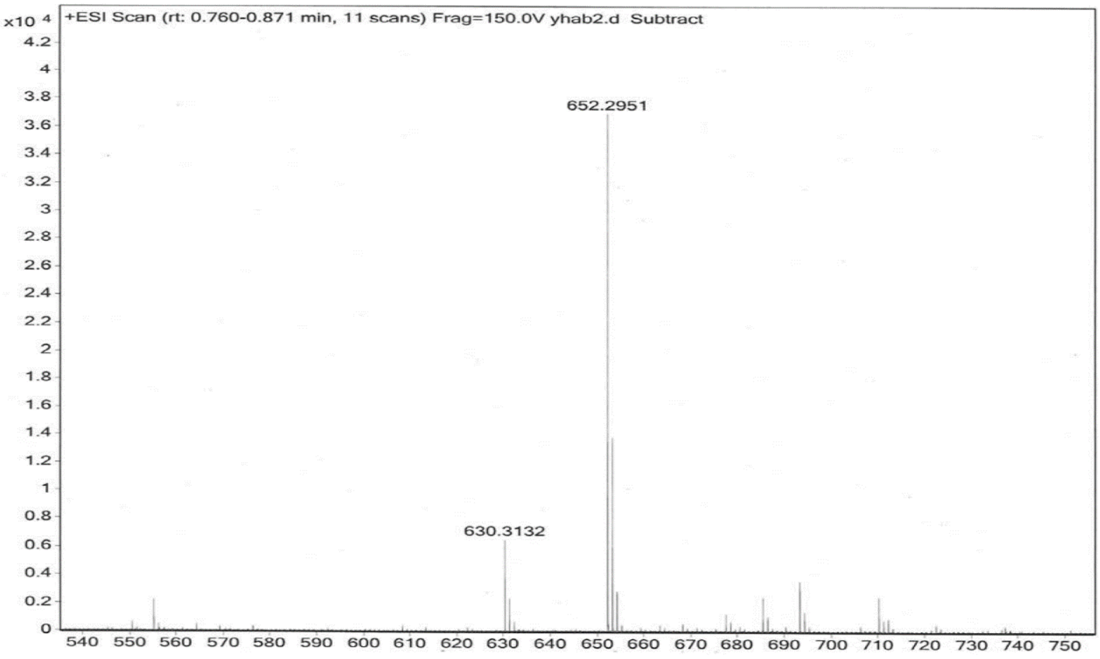
x10 4
+ESI Scan (rt: 0.760-0.871 min, 11 scans) Frag=150.0V yhab2.d Subtract
652.2951
630.3132
4.2
4
3.8
3.6
3.4
3.2
3
2.8
2.6
2.4
2.2
2
1.8
1.6
1.4
1.2
1
0.8
0.6
0.4
0.2
0
540 550 560 570 580 590 600 610 620 630 640 650 660 670 680 690 700 710 720 730 740 750

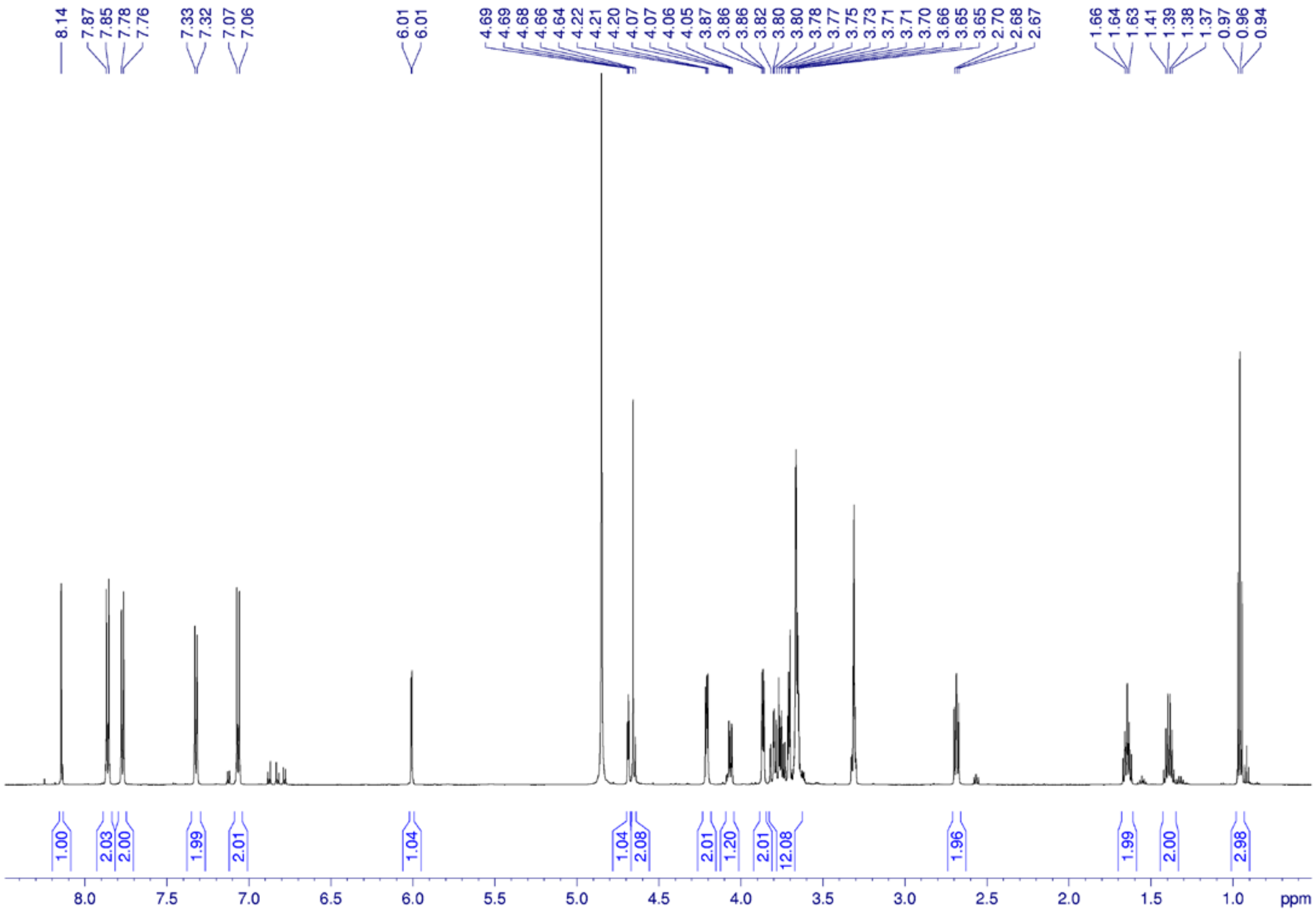
8.14
7.87
7.85
7.78
7.76
7.33
7.32
7.07
7.06
6.01
6.01
4.69
4.69
4.68
4.66
4.64
4.22
4.21
4.20
4.07
4.07
4.06
4.05
3.87
3.86
3.82
3.80
3.80
3.78
3.77
3.75
3.73
3.71
3.70
3.66
3.65
2.70
2.68
2.67
1.66
1.64
1.63
1.41
1.39
1.38
1.37
0.97
0.96
0.94
1.00
2.03
2.00
1.99
2.01
1.04
1.04
2.08
2.01
1.20
2.01
12.08
1.96
1.99
2.00
2.98
8.0
7.5
7.0
6.5
6.0
5.5
5.0
4.5
4.0
3.5
3.0
2.5
2.0
1.5
1.0
ppm

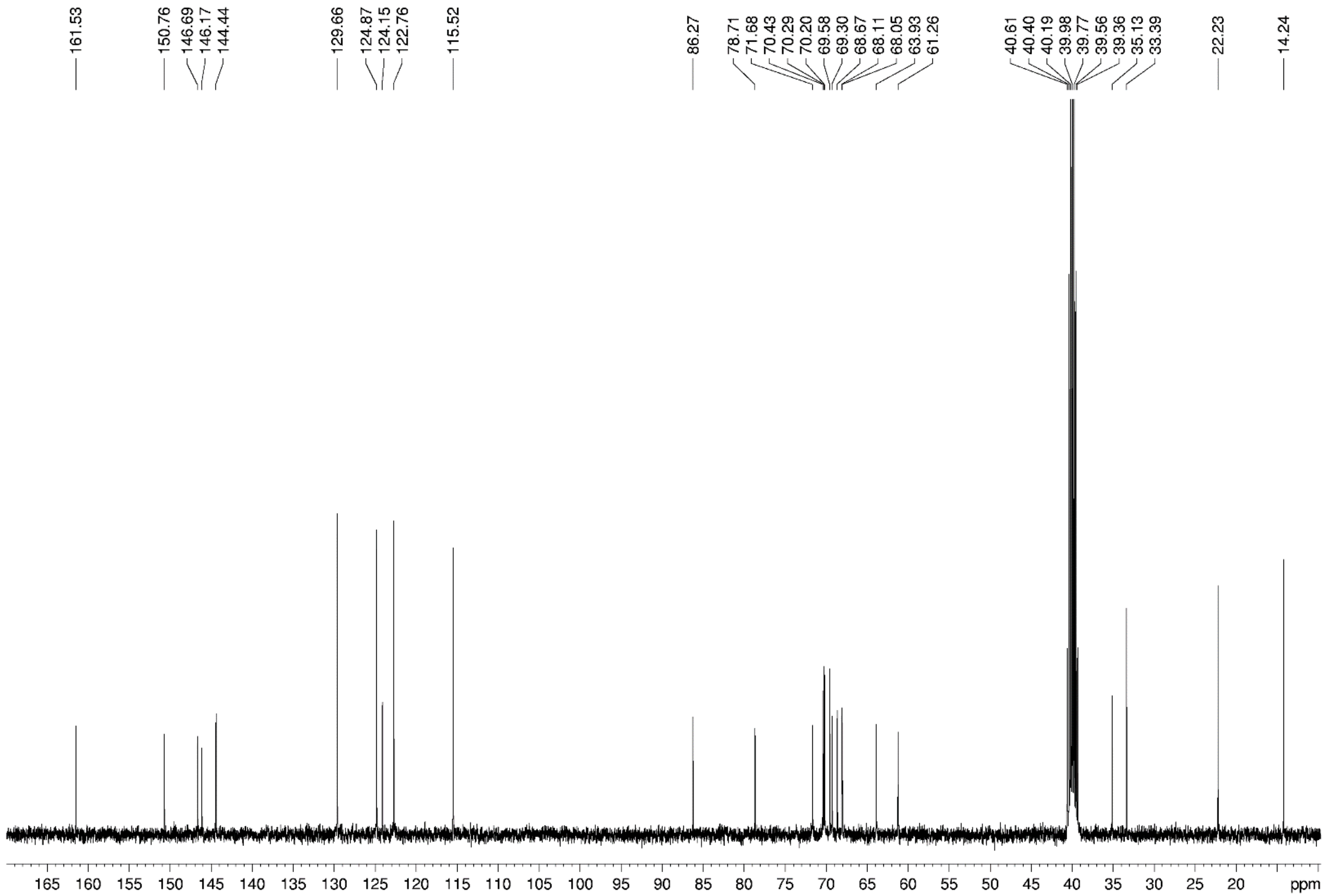

161.53
150.76
146.69
146.17
144.44
129.66
124.87
124.15
122.76
115.52
86.27
78.71
71.68
70.43
70.29
70.20
69.58
69.30
68.67
68.11
68.05
63.93
61.26
40.61
40.40
40.19
39.98
39.77
39.56
39.36
35.13
33.39
22.23
14.24
165 160 155 150 145 140 135 130 125 120 115 110 105 100 95 90 85 80 75 70 65 60 55 50 45 40 35 30 25 20 ppm

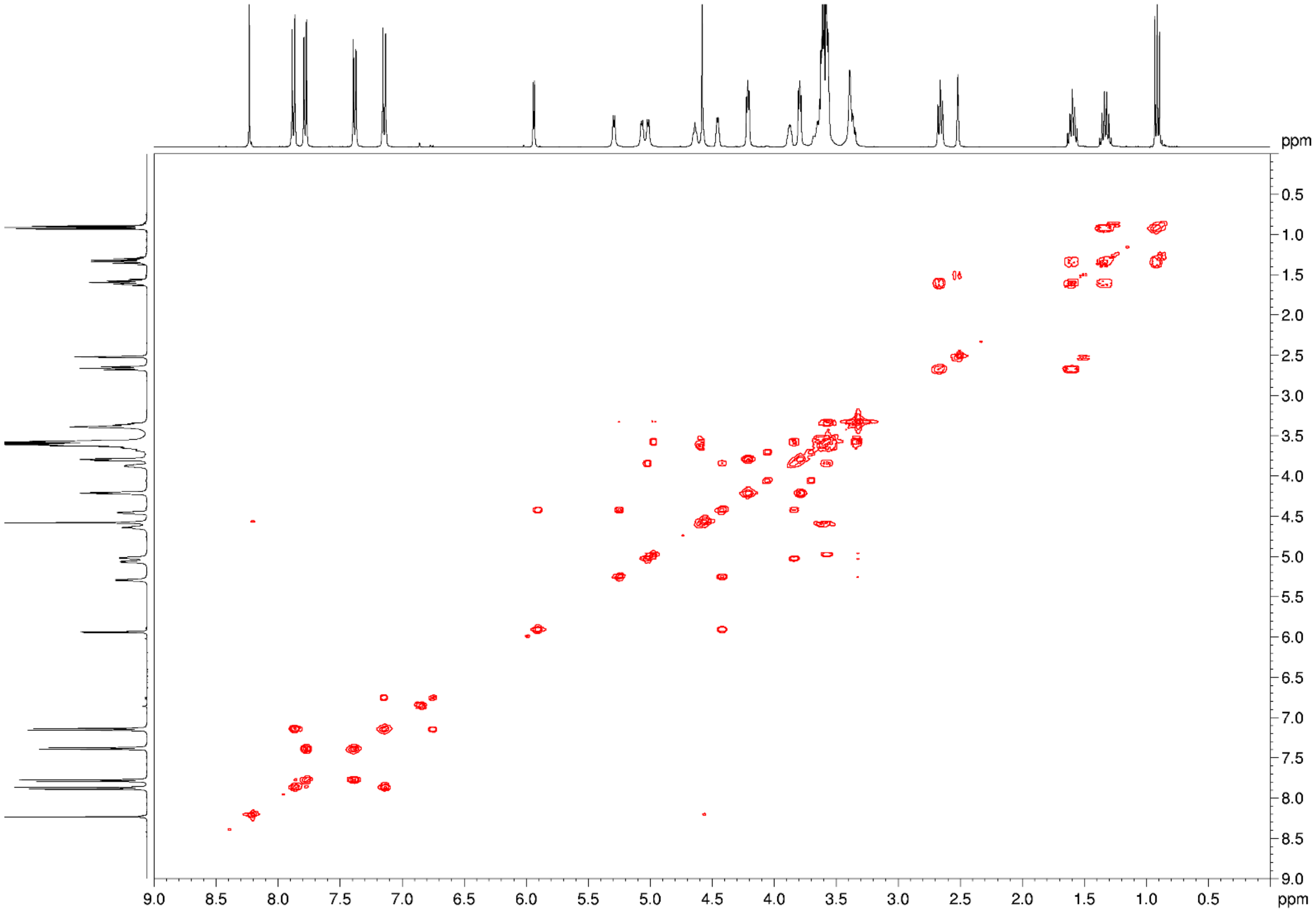
ppm
0.5
1.0
1.5
2.0
2.5
3.0
3.5
4.0
4.5
5.0
5.5
6.0
6.5
7.0
7.5
8.0
8.5
9.0
9.0
8.5
8.0
7.5
7.0
6.5
6.0
5.5
5.0
4.5
4.0
3.5
3.0
2.5
2.0
1.5
1.0
0.5
ppm

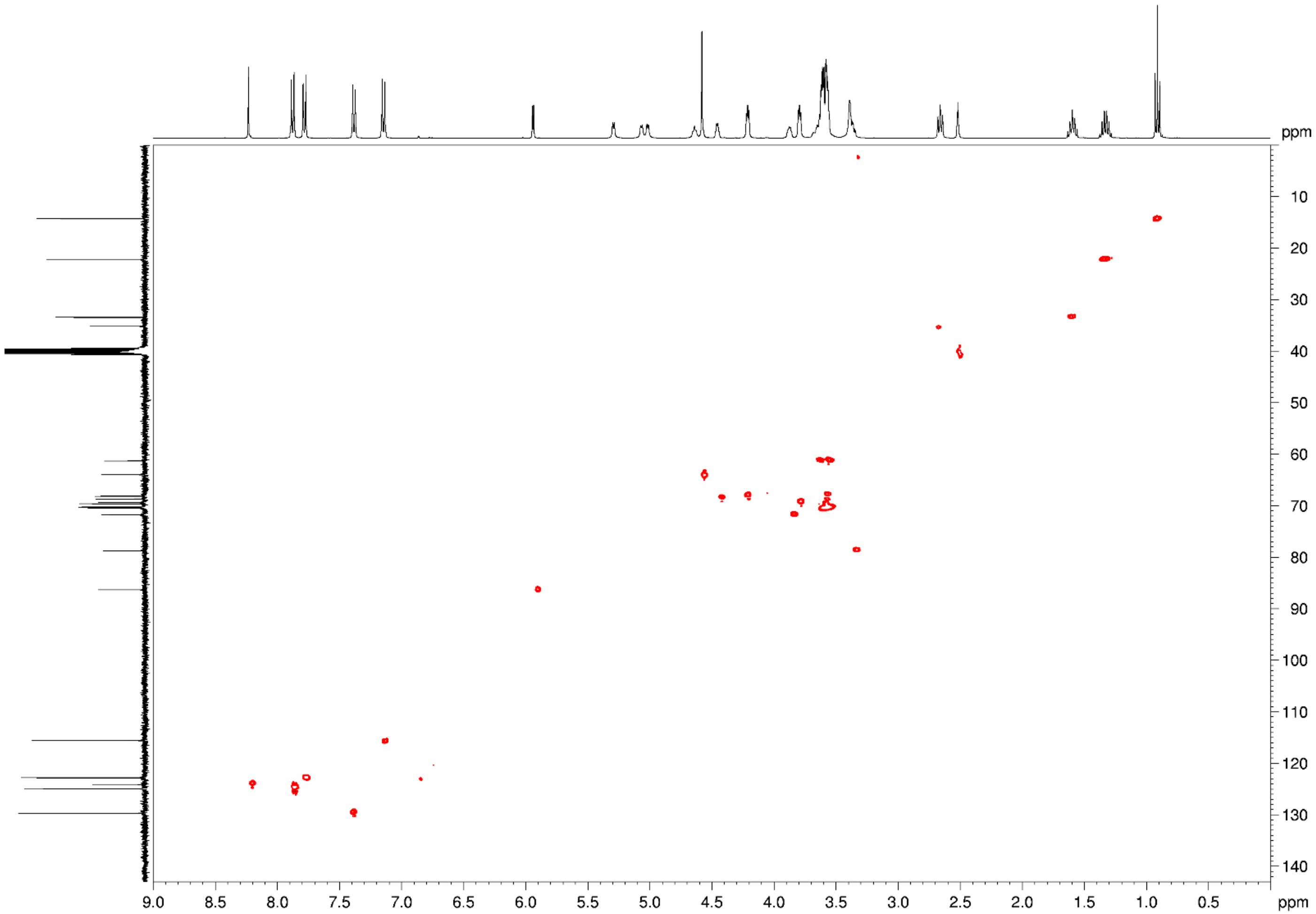
ppm
10
20
30
40
50
60
70
80
90
100
110
120
130
140
9.0
8.5
8.0
7.5
7.0
6.5
6.0
5.5
5.0
4.5
4.0
3.5
3.0
2.5
2.0
1.5
1.0
0.5
ppm

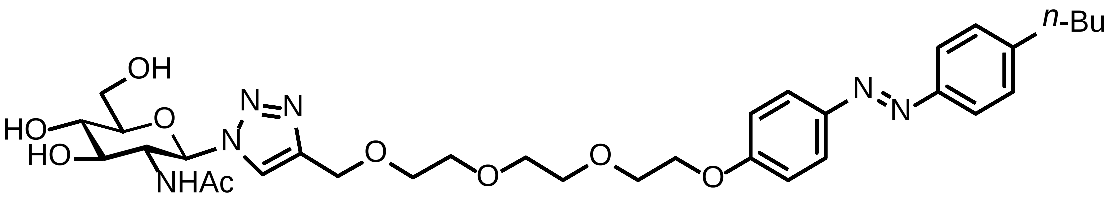
OH
HO
HO
O
NHAc
N
N=N
O
O
O
O
N
N
n-Bu

AzoGlcNAc

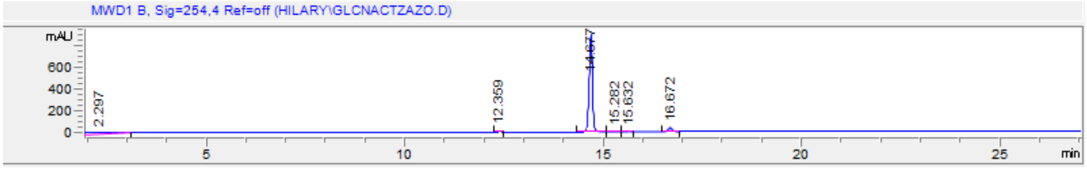
MWD1 B, Sig=254,4 Ref=off (HILARY\GLCNACTZAZO.D)
mAU
600
400
200
0
2.297
12.359
14.677
15.282
15.632
16.672
5
10
15
20
25
min

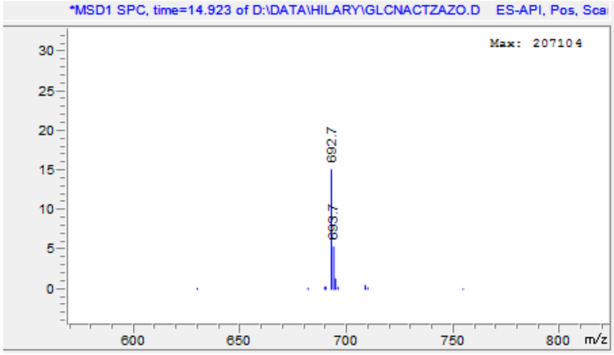
*MSD1 SPC, time=14.923 of D:\DATA\HILARY\GLCNACTZAZO.D ES-API, Pos, Sca
Max: 207104
30
25
20
15
10
5
0
692.7
693.7
600
650
700
750
800
m/z

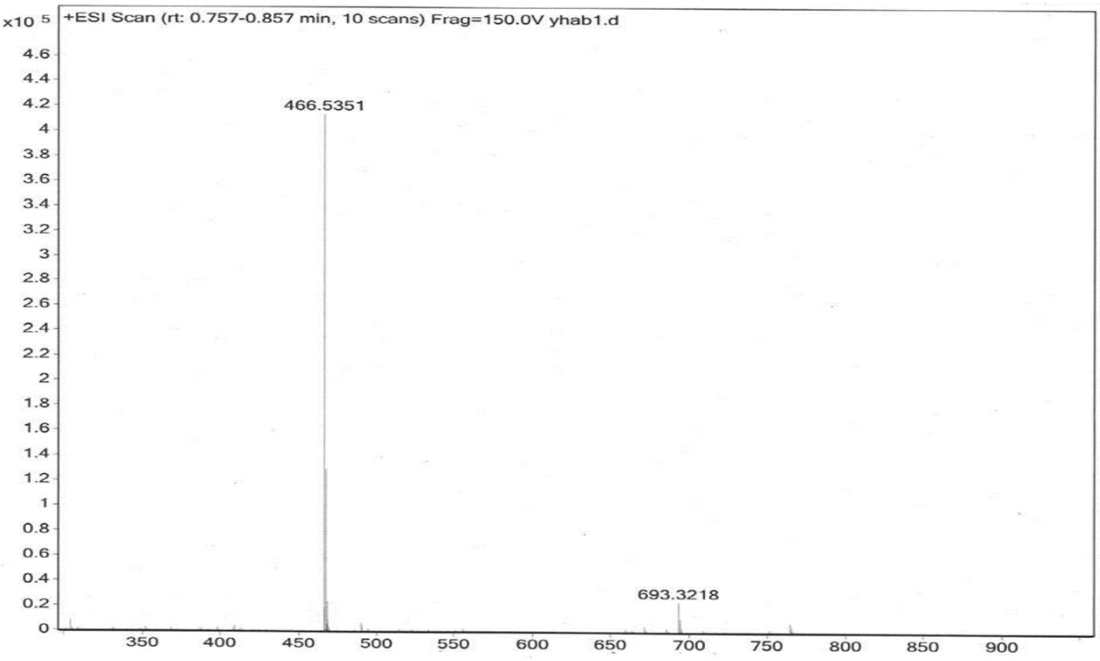
x10 5
+ESI Scan (rt: 0.757-0.857 min, 10 scans) Frag=150.0V yhab1.d
466.5351
693.3218
350
400
450
500
550
600
650
700
750
800
850
900

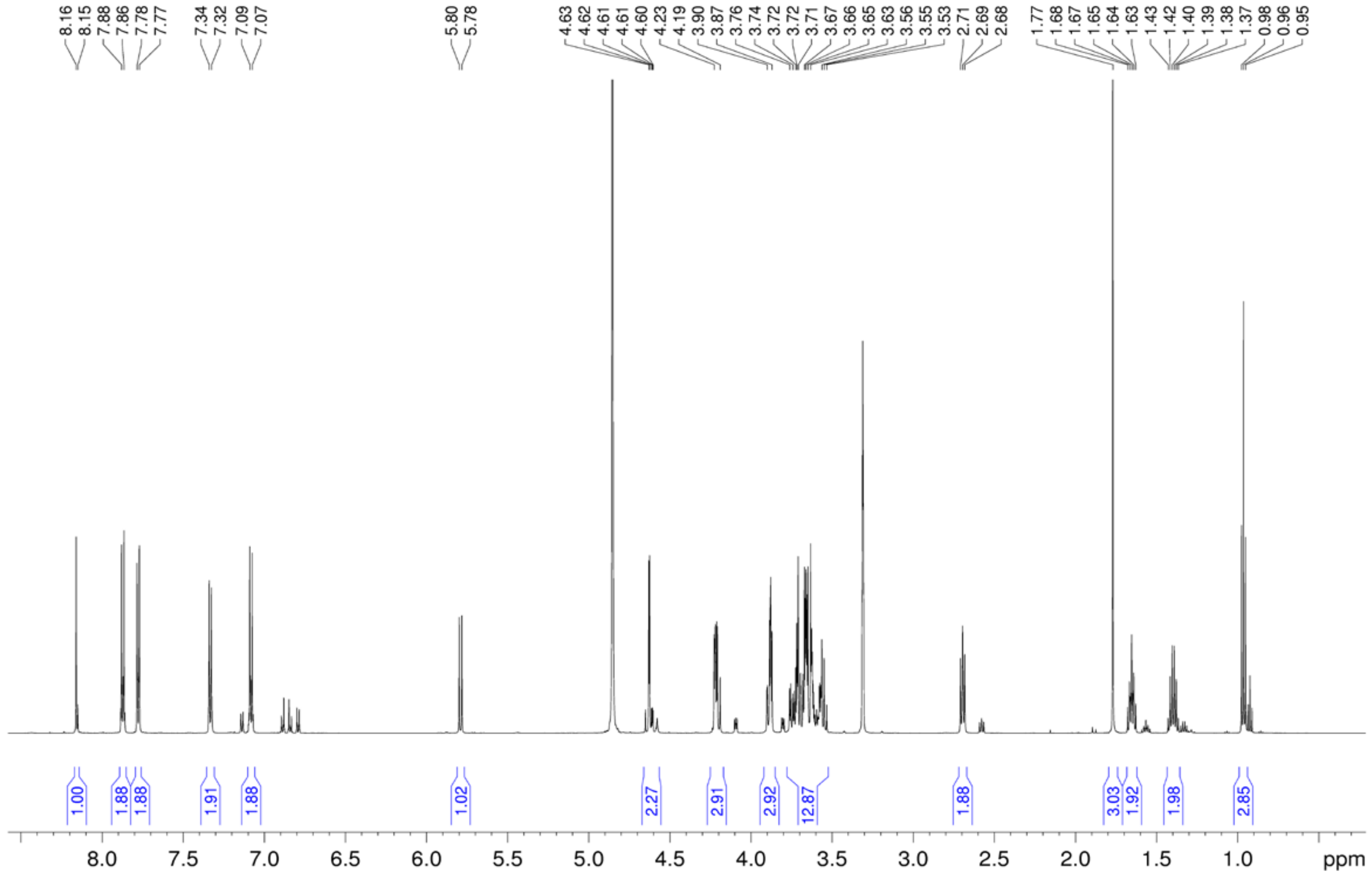
8.16 8.15 7.88 7.86 7.78 7.77 7.34 7.32 7.09 7.07
5.80 5.78
4.63 4.62 4.61 4.61 4.60 4.23 4.19 3.90 3.87 3.76 3.74 3.72 3.72 3.71 3.67 3.66 3.65 3.63 3.56 3.55 3.53 2.71 2.69 2.68
1.77 1.68 1.67 1.65 1.64 1.63 1.43 1.42 1.40 1.39 1.38 1.37 0.98 0.96 0.95
1.00 1.88 1.88 1.91 1.88 1.02 2.27 2.91 2.92 12.87 1.88 3.03 1.92 1.98 2.85
8.0 7.5 7.0 6.5 6.0 5.5 5.0 4.5 4.0 3.5 3.0 2.5 2.0 1.5 1.0 ppm

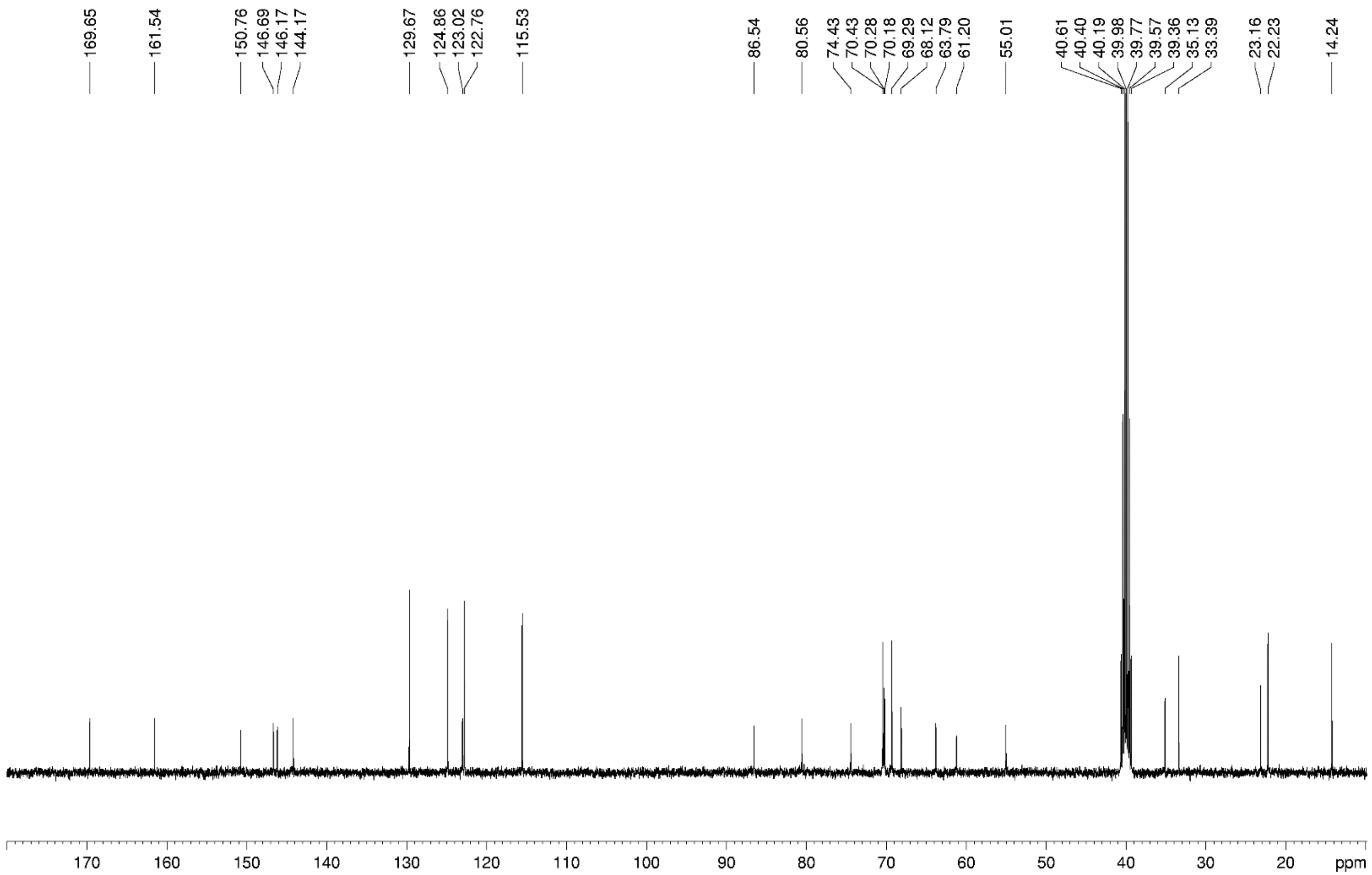

169.65
161.54
150.76
146.69
146.17
144.17
129.67
124.86
123.02
122.76
115.53
86.54
80.56
74.43
70.43
70.28
70.18
69.29
68.12
63.79
61.20
55.01
40.61
40.40
40.19
39.98
39.77
39.57
39.36
35.13
33.39
23.16
22.23
14.24
170 160 150 140 130 120 110 100 90 80 70 60 50 40 30 20 ppm

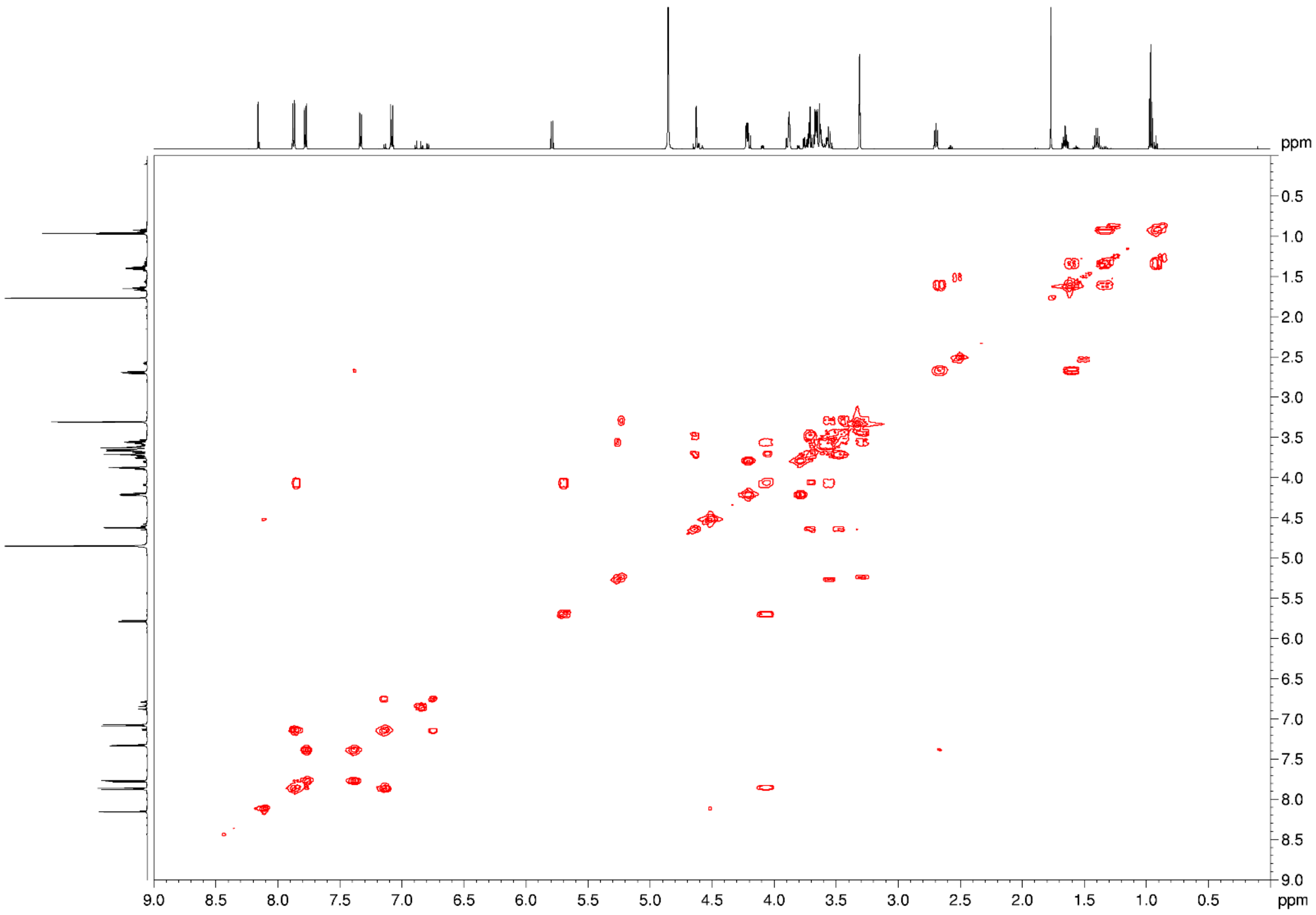

ppm
0.5
1.0
1.5
2.0
2.5
3.0
3.5
4.0
4.5
5.0
5.5
6.0
6.5
7.0
7.5
8.0
8.5
9.0
ppm
9.0
8.5
8.0
7.5
7.0
6.5
6.0
5.5
5.0
4.5
4.0
3.5
3.0
2.5
2.0
1.5
1.0
0.5
ppm

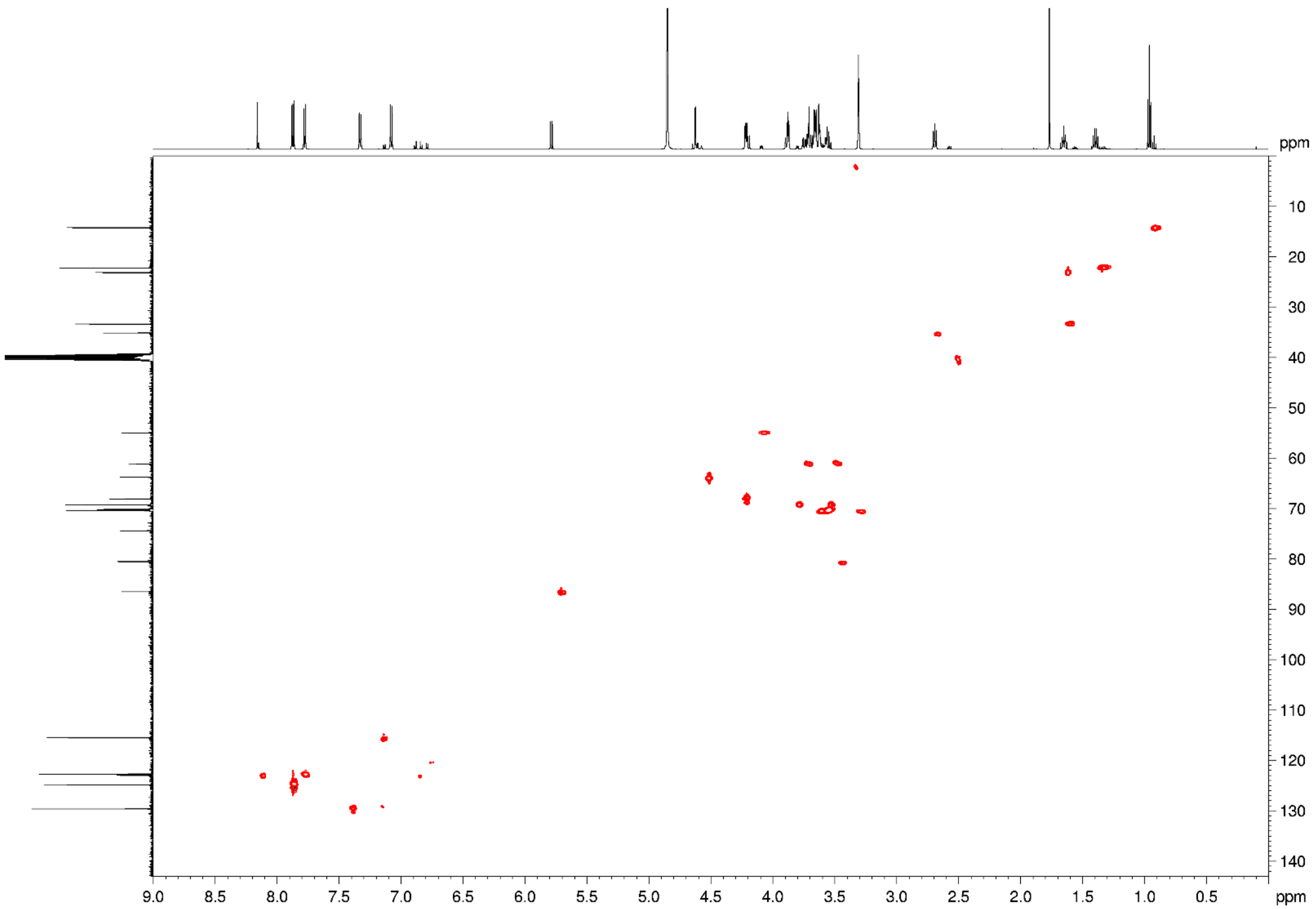
ppm
10
20
30
40
50
60
70
80
90
100
110
120
130
140
9.0
8.5
8.0
7.5
7.0
6.5
6.0
5.5
5.0
4.5
4.0
3.5
3.0
2.5
2.0
1.5
1.0
0.5
ppm

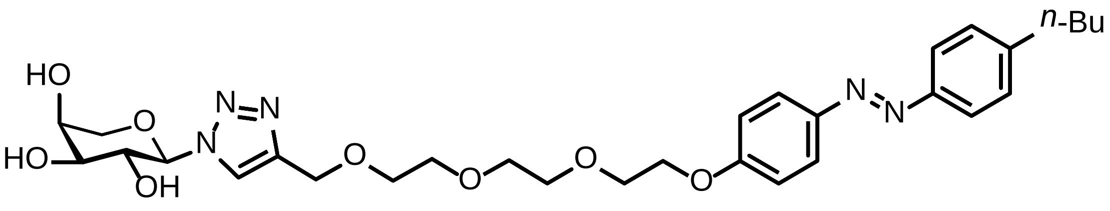
HO
O
HO
OH
N
N
N
O
O
O
O
N
N
n-Bu

AzoAra

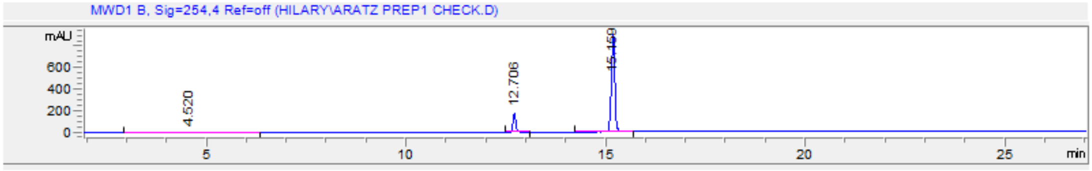
MWD1 B, Sig=254,4 Ref=off (HILARY\ARATZ PREP1 CHECK.D)
mAU
600
400
200
0
4.520
12.706
15.159
5
10
15
20
25
min

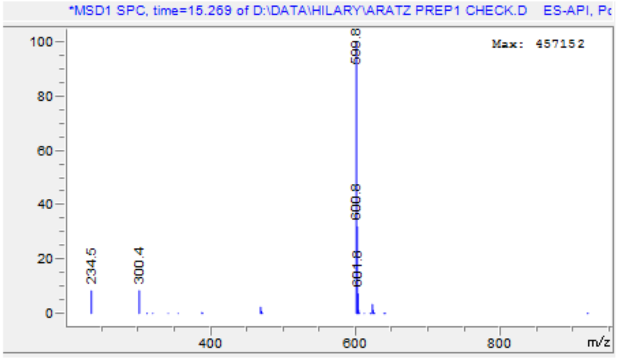
*MSD1 SPC, time=15.269 of D:\DATA\HILARY\ARATZ PREP1 CHECK.D ES-API, Pc
Max: 457152
100
80
60
40
20
0
234.5
300.4
599.8
600.8
601.8
400
600
800
m/z

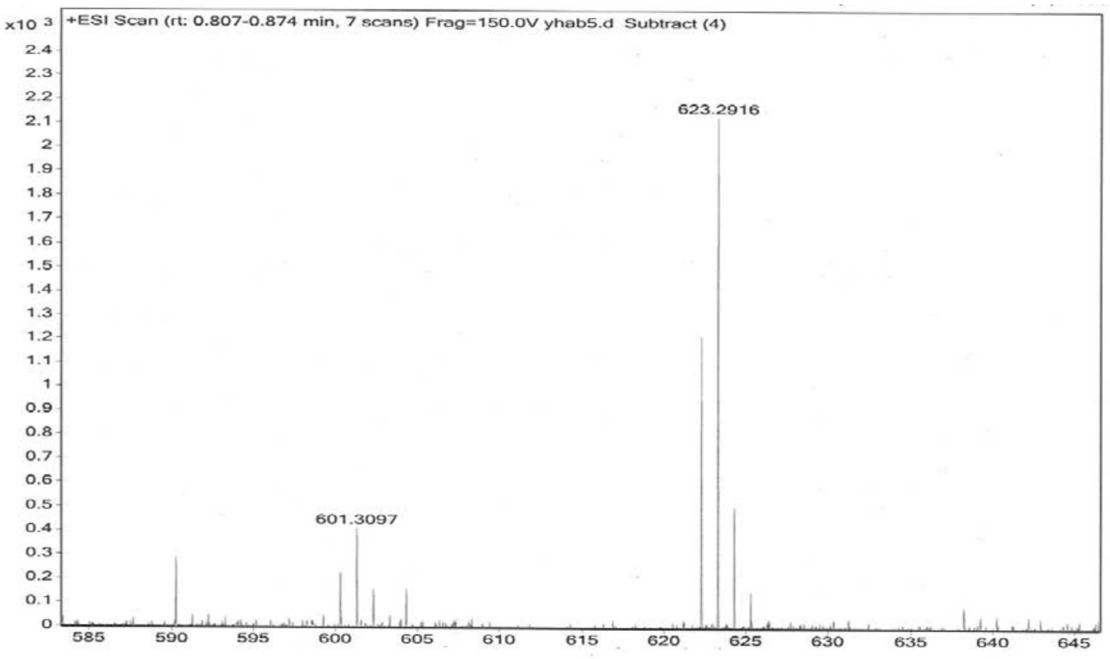
x10 3
+ESI Scan (rt: 0.807-0.874 min, 7 scans) Frag=150.0V yhab5.d Subtract (4)
623.2916
601.3097
585
590
595
600
605
610
615
620
625
630
635
640
645

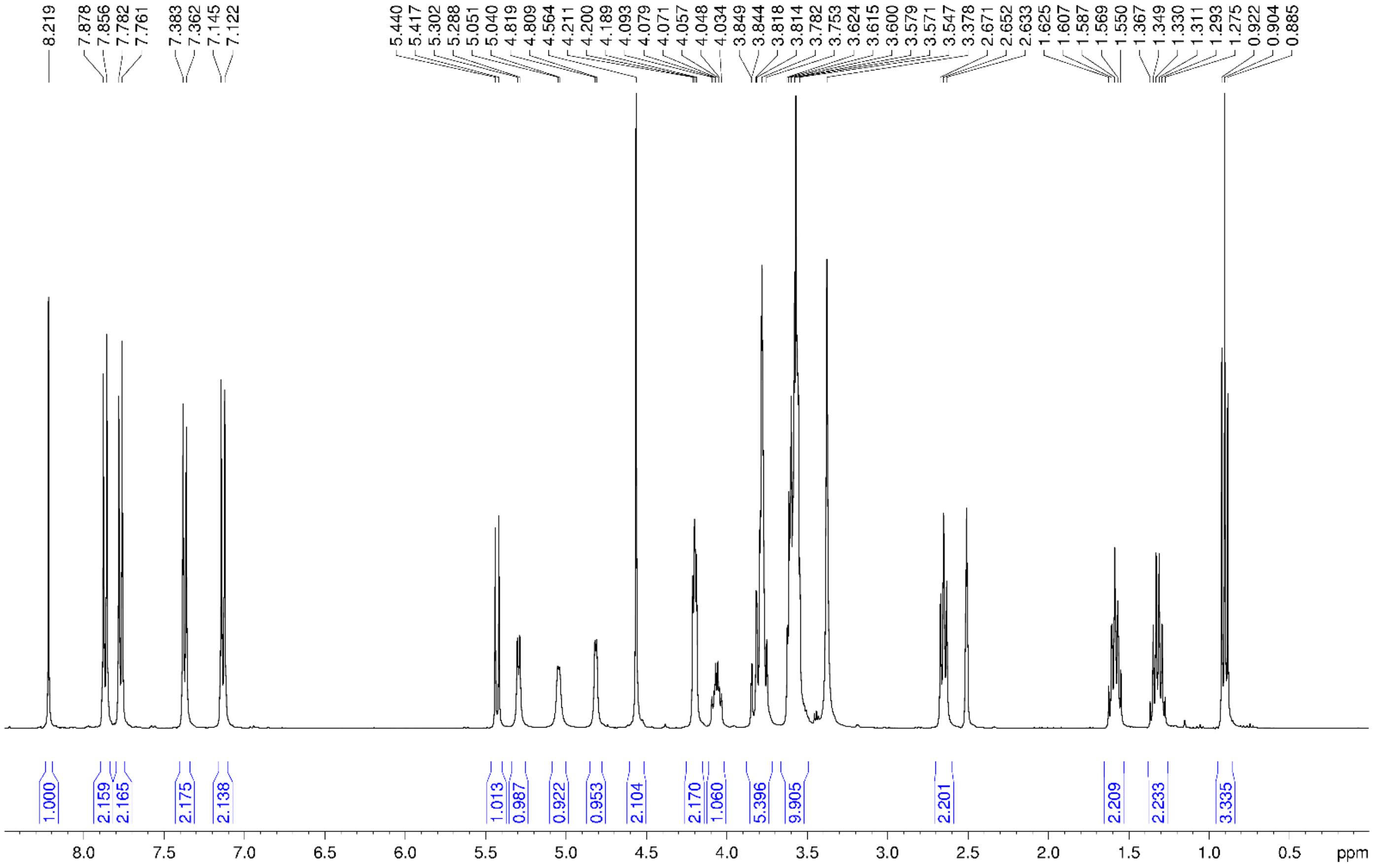

8.219
7.878
7.856
7.782
7.761
7.383
7.362
7.145
7.122
5.440
5.417
5.302
5.288
5.051
5.040
4.819
4.809
4.564
4.211
4.200
4.189
4.093
4.079
4.071
4.057
4.048
4.034
3.849
3.844
3.818
3.814
3.782
3.753
3.624
3.615
3.600
3.579
3.571
3.547
3.378
2.671
2.652
2.633
1.625
1.607
1.587
1.569
1.550
1.367
1.349
1.330
1.311
1.293
1.275
0.922
0.904
0.885
1.000
2.159
2.165
2.175
2.138
1.013
0.987
0.922
0.953
2.104
2.170
1.060
5.396
9.905
2.201
2.209
2.233
3.335
8.0
7.5
7.0
6.5
6.0
5.5
5.0
4.5
4.0
3.5
3.0
2.5
2.0
1.5
1.0
0.5
ppm

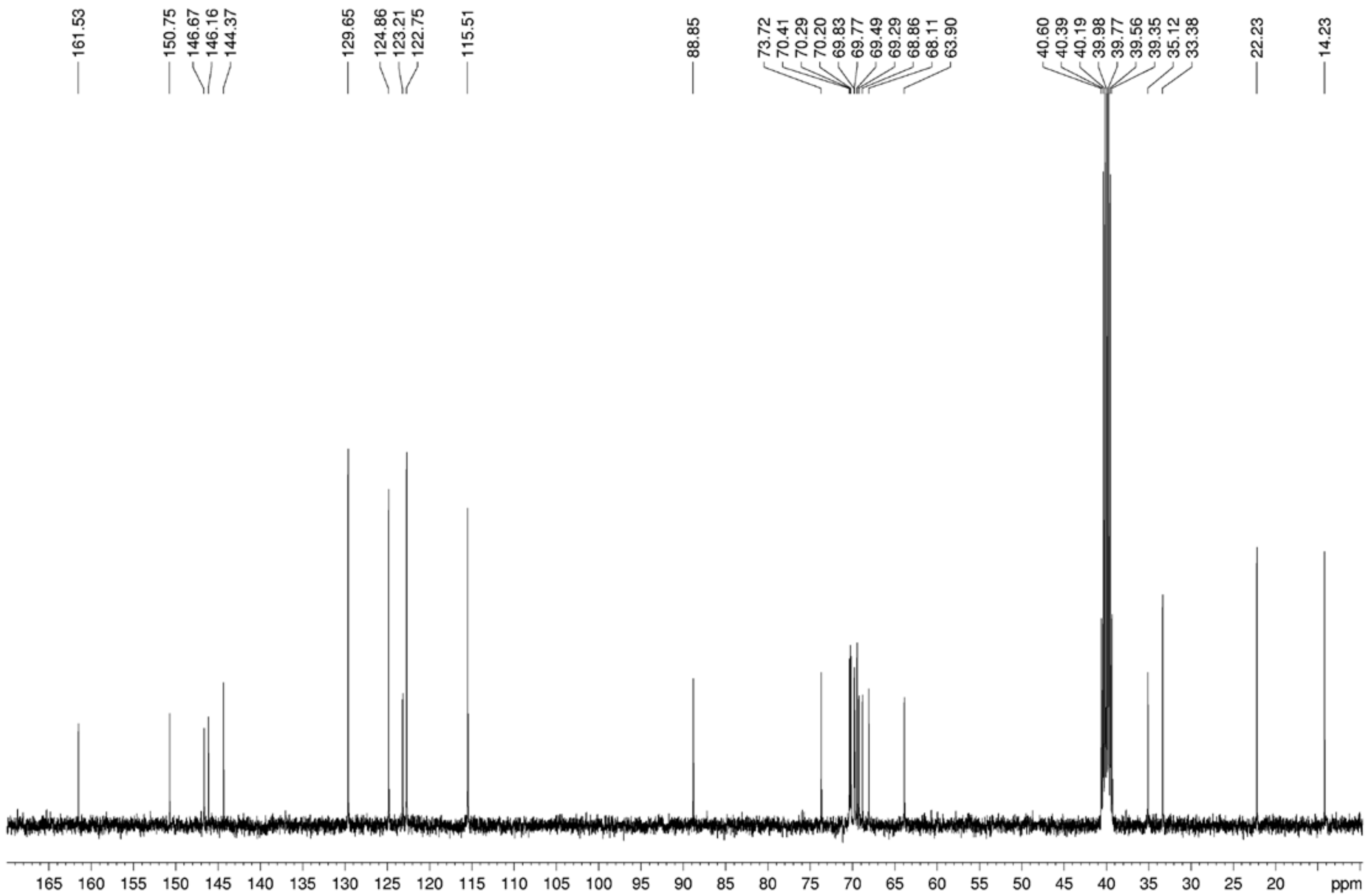

161.53
150.75
146.67
146.16
144.37
129.65
124.86
123.21
122.75
115.51
88.85
73.72
70.41
70.29
70.20
69.83
69.77
69.49
69.29
68.86
68.11
63.90
40.60
40.39
40.19
39.98
39.77
39.56
39.35
35.12
33.38
22.23
14.23
165 160 155 150 145 140 135 130 125 120 115 110 105 100 95 90 85 80 75 70 65 60 55 50 45 40 35 30 25 20 ppm

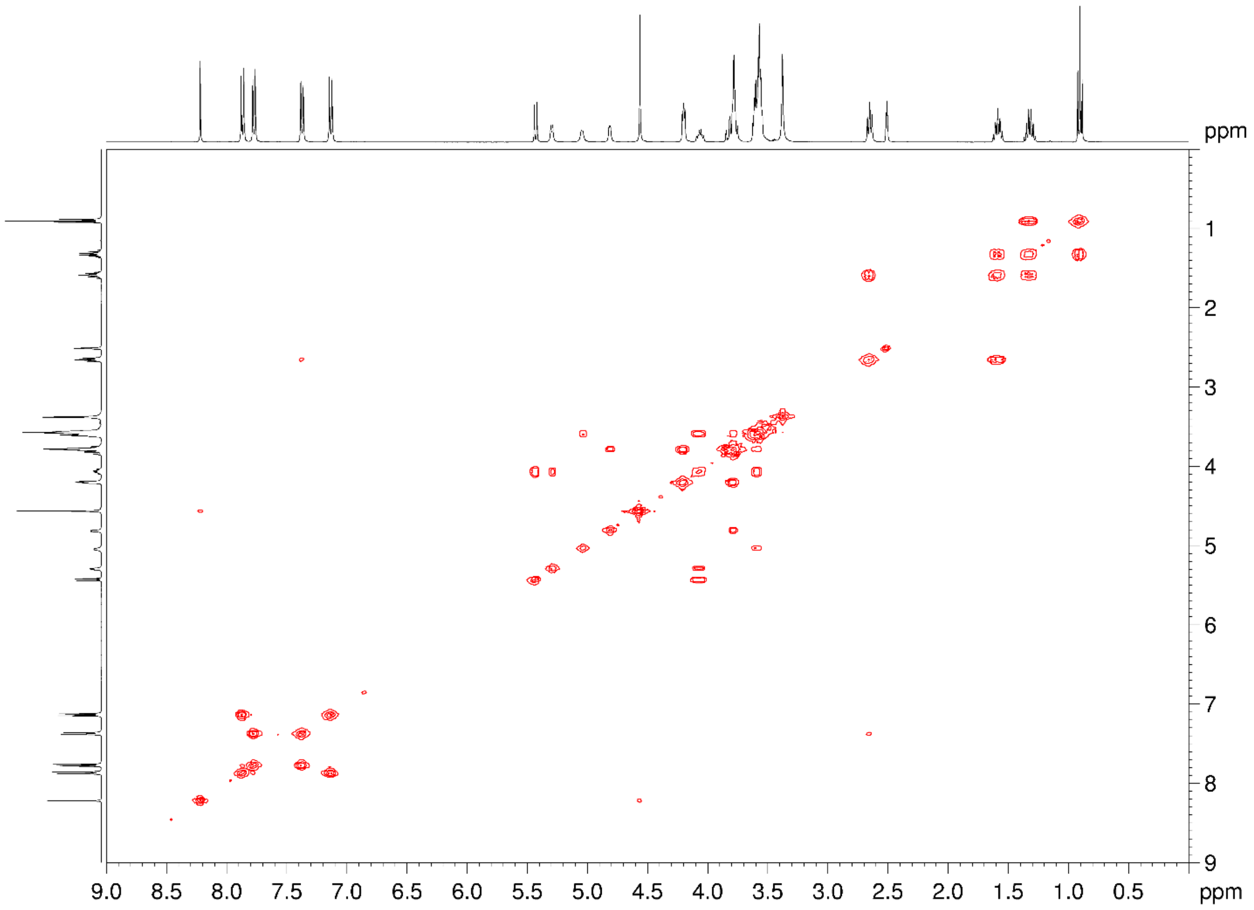

ppm
9.0 8.5 8.0 7.5 7.0 6.5 6.0 5.5 5.0 4.5 4.0 3.5 3.0 2.5 2.0 1.5 1.0 0.5
ppm
1 2 3 4 5 6 7 8 9

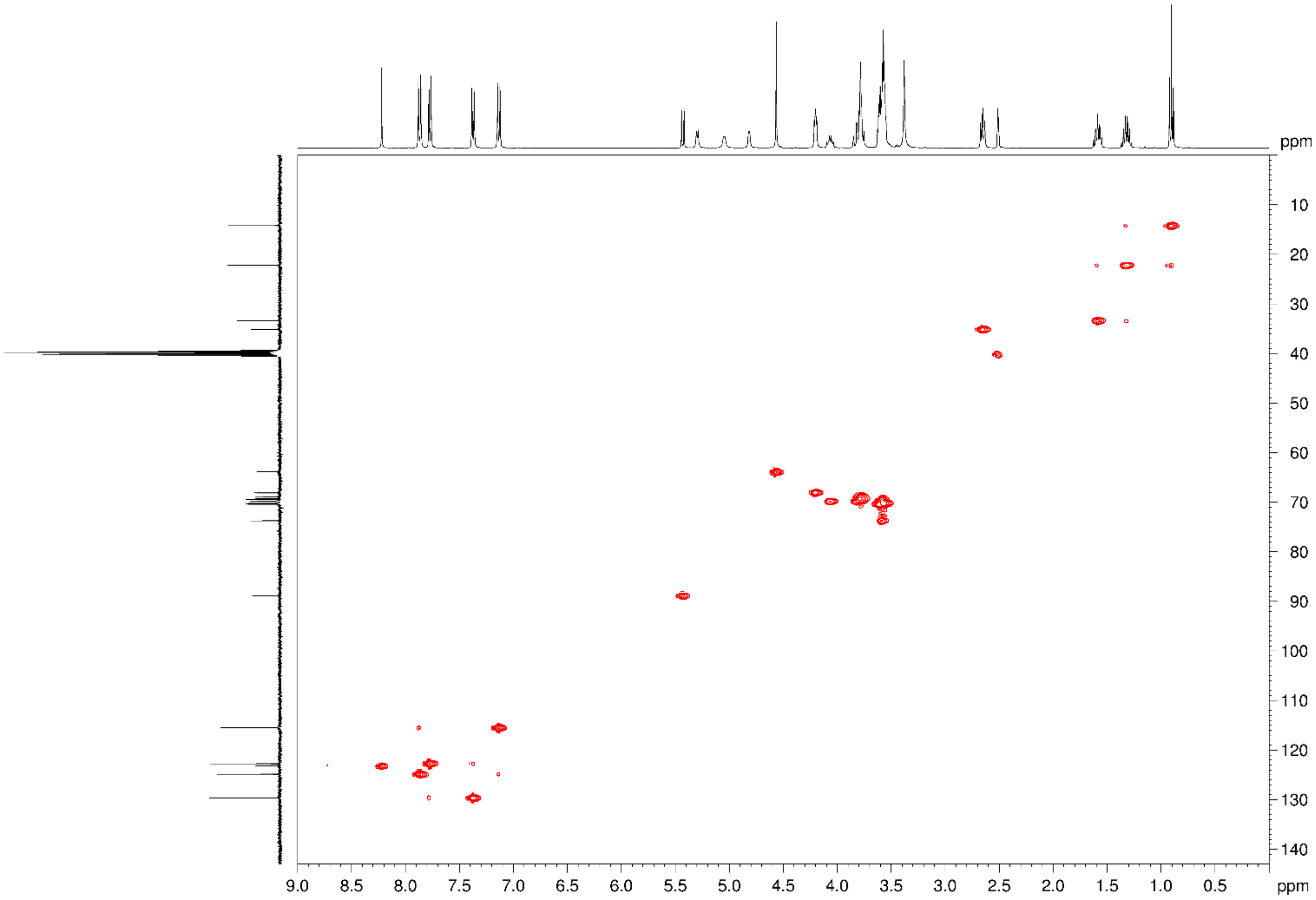
ppm
10
20
30
40
50
60
70
80
90
100
110
120
130
140
9.0 8.5 8.0 7.5 7.0 6.5 6.0 5.5 5.0 4.5 4.0 3.5 3.0 2.5 2.0 1.5 1.0 0.5 ppm
